# IPr*^diNHC^: Sterically Adaptable Dinuclear N-Heterocyclic Carbenes

**DOI:** 10.1021/acs.inorgchem.5c01013

**Published:** 2025-04-15

**Authors:** Katarzyna Halikowska-Tarasek, Wioletta Ochędzan-Siodłak, Błażej Dziuk, Roman Szostak, Michal Szostak, Elwira Bisz

**Affiliations:** † Department of Chemistry and Pharmacy, Opole University, 48 Oleska Street, Opole 45-052, Poland; ‡ Department of Chemistry, Wroclaw University of Science and Technology, Norwida 4/6, Wroclaw 50-373, Poland; § Department of Chemistry, Wroclaw University, F. Joliot-Curie 14, Wroclaw 50-383, Poland; ∥ Department of Chemistry, Rutgers University, 73 Warren Street, Newark, New Jersey 07102, United States

## Abstract

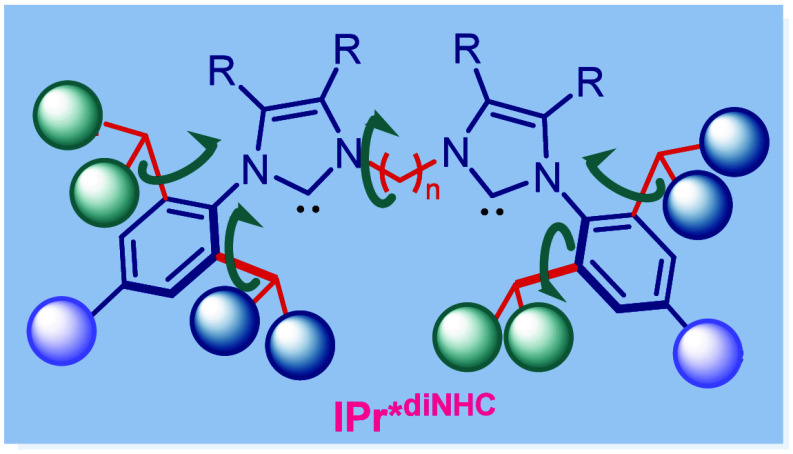

N-Heterocyclic carbenes (NHCs) have been established as the predominant ligand class in inorganic and organometallic chemistry. Simultaneously, there has been a major interest in dinuclear ligands, where cooperative effects between the metal centers have been widely exploited across numerous research avenues. Herein, we report the synthesis of dinuclear sterically hindered and adaptable N-heterocyclic ligands based on the modular IPr* framework. These ligands are distinguished by (1) the bridging group between the N-heterocyclic carbene donors and (2) the steric and electronic characteristics of the N-aromatic wingtips. The net effect is a remarkably broadly ranging steric environment around the metal center (%V_bur_ of 33.9 up to 60.4%) as well as versatile conformation ranging from the perfectly linear (3.2°) to almost fully perpendicular (79.3°) between the carbene donors. The ligands have been evaluated in the gold(I)-catalyzed hydration and carboxylation of alkynes, where they show enhanced catalytic reactivity over mononuclear gold(I) complexes. Considering the importance of dinuclear N-heterocyclic carbenes across different research fields, we expect that this ligand class will be of broad interest in inorganic and organometallic chemistry.

## Abstract 2

N-Heterocyclic carbenes (NHCs) have been established as the predominant ligand class in inorganic and organometallic chemistry. Simultaneously, there has been a major interest in dinuclear ligands, where cooperative effects between the metal centers have been widely exploited across numerous research avenues. Herein, we report the synthesis of dinuclear sterically hindered and adaptable N-heterocyclic ligands based on the modular IPr* framework. These ligands are distinguished by (1) the bridging group between the N-heterocyclic carbene donors and (2) the steric and electronic characteristics of the N-aromatic wingtips.

Since the seminal studies by Arduengo in 1991,^1a^ N-heterocyclic carbenes have been established as the major class of ligands in chemistry.^1b,1c^ In particular, the unique combination of their strong σ-donation engendered by the N-heterocyclic carbene center together with the tightly controlled steric environment fashioned by the N-wingtips have provided significant advantages over traditional ligand systems in stabilization of reactive metals.^2^ This singular ability of N-heterocyclic carbenes has been broadly utilized across various research fields, including inorganic and organometallic chemistry, catalysis, medicinal chemistry, and materials science.^3^ The capacity of NHC ligands to stabilize metals at various oxidation states has enabled remarkable advances in the isolation of elusive metal complexes and enhancement of the reactivity in transition metal catalysis. Over the past decades, the major advances have been accomplished by the development of sterically hindered N-heterocyclic carbenes that provide a flexible steric environment, as elegantly demonstrated by Nolan,^4^ Glorius,^5^ Bertrand,^6^ and others^7^ (Figure 1B).

**Figure 1:**
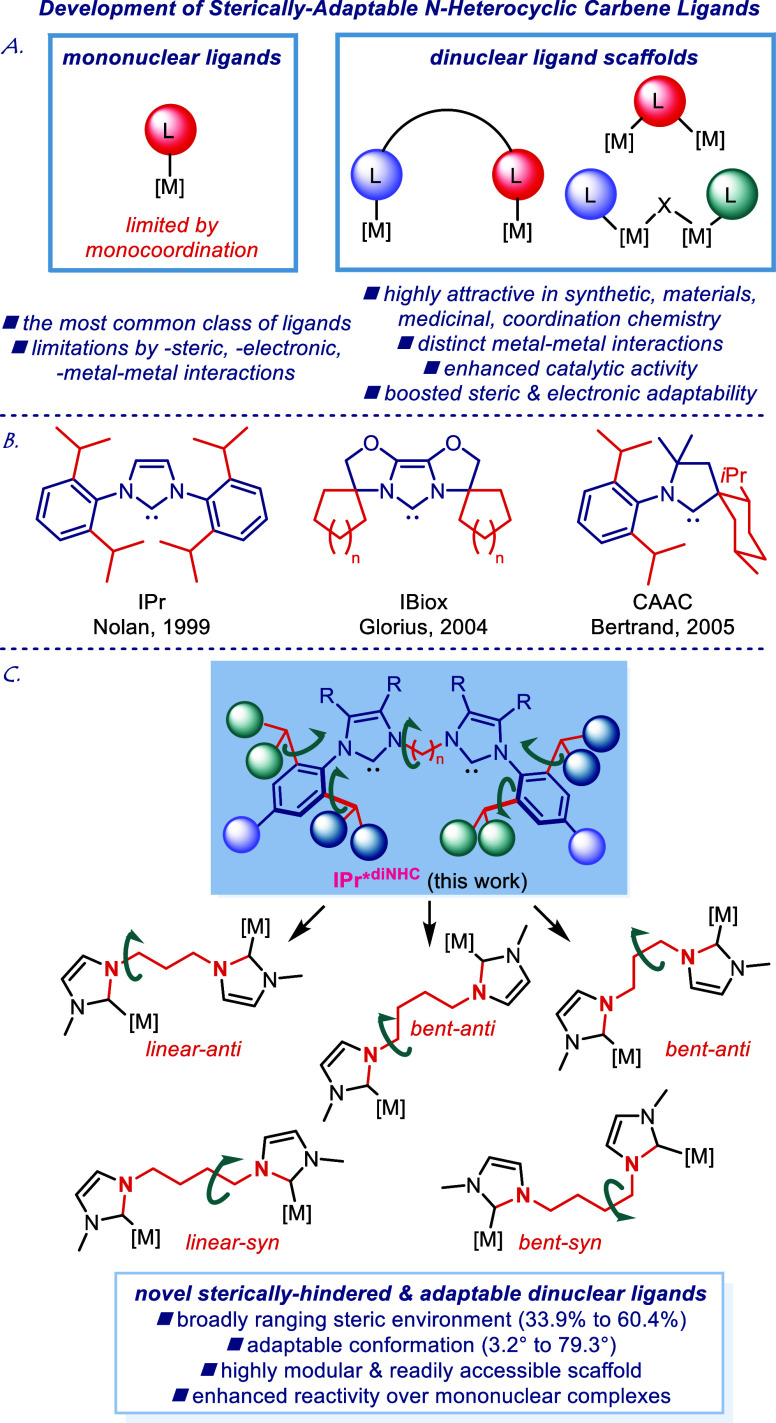
(A) Graphical representation of mononuclear and dinuclear complexes. (B) Examples of sterically adaptable mononuclear N-heterocyclic carbenes in inorganic and organometallic chemistry.^4a,5a,6a^ (C) This study: sterically adaptable dinuclear N-heterocyclic carbenes.

Simultaneously, there has been a major interest in dinuclear complexes that feature two donor centers in a close proximity that enables their cooperation during catalysis (Figure 1A).^8^ Dinuclear N-heterocyclic carbene ligands have found broad applicability across a diverse range of catalytic avenues, including Au, Pd, Ru, Cu, Ag, and Ir catalysis,^9^ where the coordination of N-heterocyclic carbenes and the resulting interaction between the metal centers and ligands allowed for unique steric and electronic properties.^10^ However, one of the major challenges has been the synthesis of well-defined, adaptable dinuclear N-heterocyclic ligands that provide a novel sterically demanding but flexible environment for organometallic complexes.

In view of our interest in ligand development, we considered a new class of dinuclear N-heterocyclic carbenes based on the modular IPr* (IPr* = (2,6-bis(diphenylmethyl)-4-methylphenyl)imidazol-2-ylidene) framework. Herein, we report well-defined and adaptable dinuclear ligands with bridging N-heterocyclic carbenes that are distinguished by (1) the bridging group between the N-heterocyclic carbene donors and (2) the steric and electronic characteristics of the N-aromatic wingtips (Figure 1C). The overall effect is a remarkably broadly ranging steric environment around the metal center (%V_bur_ of 33.9 up to 60.4%) as well as adaptable conformation ranging from perfectly linear (3.2°) to almost fully perpendicular (79.3°) between the carbene donors. These dinuclear complexes show enhanced reactivity over mononuclear gold(I) complexes in gold(I)-catalyzed hydration and carboxylation of alkynes. We expect that these dinuclear carbene ligands will find broad application in various facets of inorganic and organometallic chemistry.

Our study started with the preparation of imidazolium precursors (Scheme 1). *N*-Arylimidazoles **2a–2c** featuring IPr*, IPr*^OMe^, and IPaul substitution (IPr* = 4-Me; IPr*^MeO^ = 4-MeO; IPaul = 2,4-Me_2_) were prepared by our previously optimized route from the corresponding anilines, diacetyl and paraformaldehyde, in the presence of NH_4_OAc and AcOH in CHCl_3_ at reflux (Scheme 1A).^11^ This approach allows for a robust one-pot synthesis of sterically hindered *N*-Ar imidazoles for the synthesis of unsymmetrical imidazolium precursors. The use of 3,4-backbone substitution locks the conformation of the *N*-Ar ring from rotation and permits synthetic reproducibility. The selection of IPr*, IPr*^MeO^, and IPaul was based on the fact that these imidazol-2-ylidenes are the most commonly used sterically hindered N-heterocyclic carbenes, while the presence of the MeO substitution leads to an enhanced σ-donation (vs Me) and the use of IPaul (vs IPr* and IPr*^MeO^) permits for an enhanced spatial flexibility of the N-aromatic wingtip.^7,11,12^*N*-Arylimidazoles **2a–2c** were subjected to alkylation with 1,3-dicholoropropane and 1,4-dichlorobutane in acetonitrile at 110 °C to afford bis-imidazolium precursors with the procarbenic centers separated by C3 and C4 linkers (**3a–3f**) (Scheme 1B).^13^ Mononuclear imidazolium **3g** (R = *n*-Bu) was synthesized as a control mononuclear precursor (Scheme 1B).^14^ Complexation with Au(I) was accomplished by using Au(SMe_2_)Cl in the presence of a mild carbonate base K_2_CO_3_ with the addition of LiBr in acetonitrile at 60 °C to afford dinuclear Au(I)–IPr*^diNHC^ complexes **4a–4f** (Scheme 2).^10h,15^ The synthesis of a model mononuclear complex Au(I)–IPr*^n-Bu^**4g** was accomplished by the same procedure (Scheme 3). All imidazolium salts **4a–4f** and Au(I)–IPr*^diNHC^ complexes were found to be stable to air and moisture. All dinuclear Au(I)–IPr*^diNHC^ complexes **4a–4f** and the mononuclear complex **4g** were crystalline and characterized by X-ray analysis (Figure 2). The most pertinent details of the crystallographic analysis are summarized in Table 1. Full details, including bond lengths and angles, are provided in the Supporting Information.

**Scheme 1:**
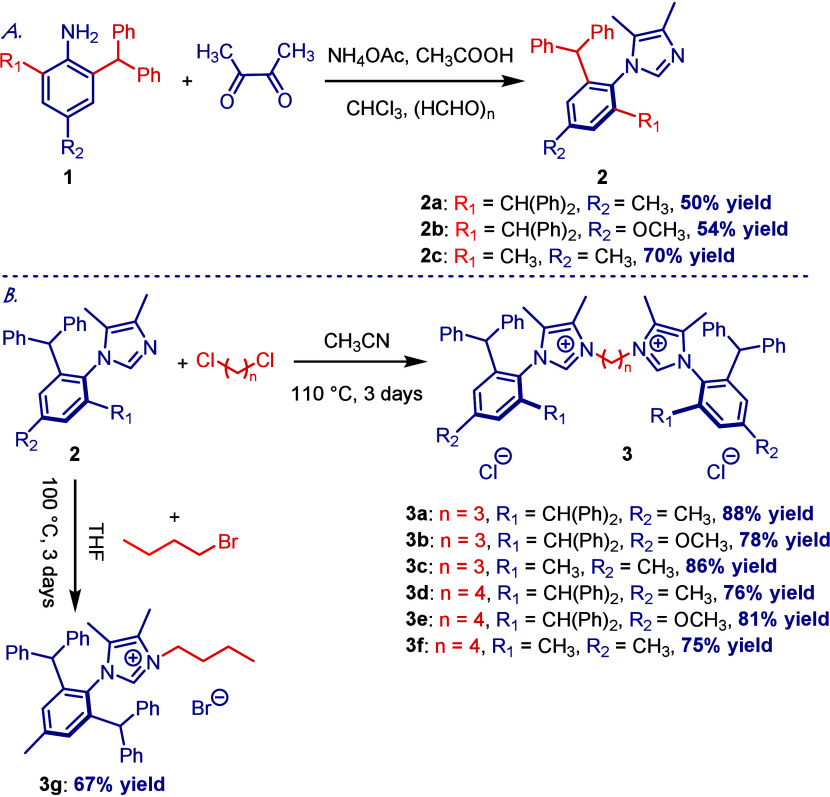
Synthesis of Imidazolium Precursors

**Scheme 2:**
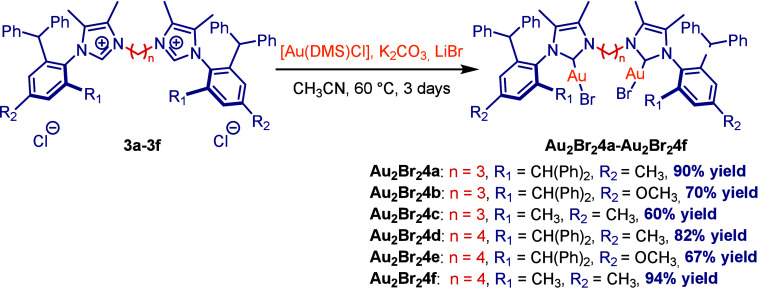
Synthesis of Dinuclear Au(I)–NHC Complexes **4a–4f**

**Scheme 3:**
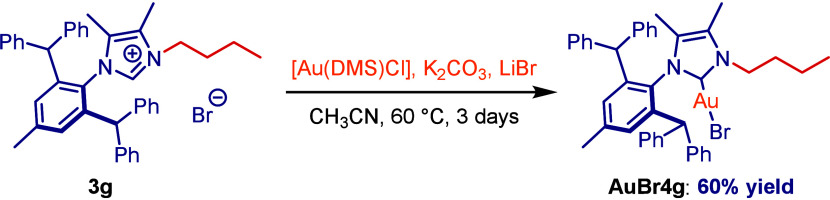
Synthesis of Mononuclear Au(I)–NHC Complex **4g**

**Figure 2:**
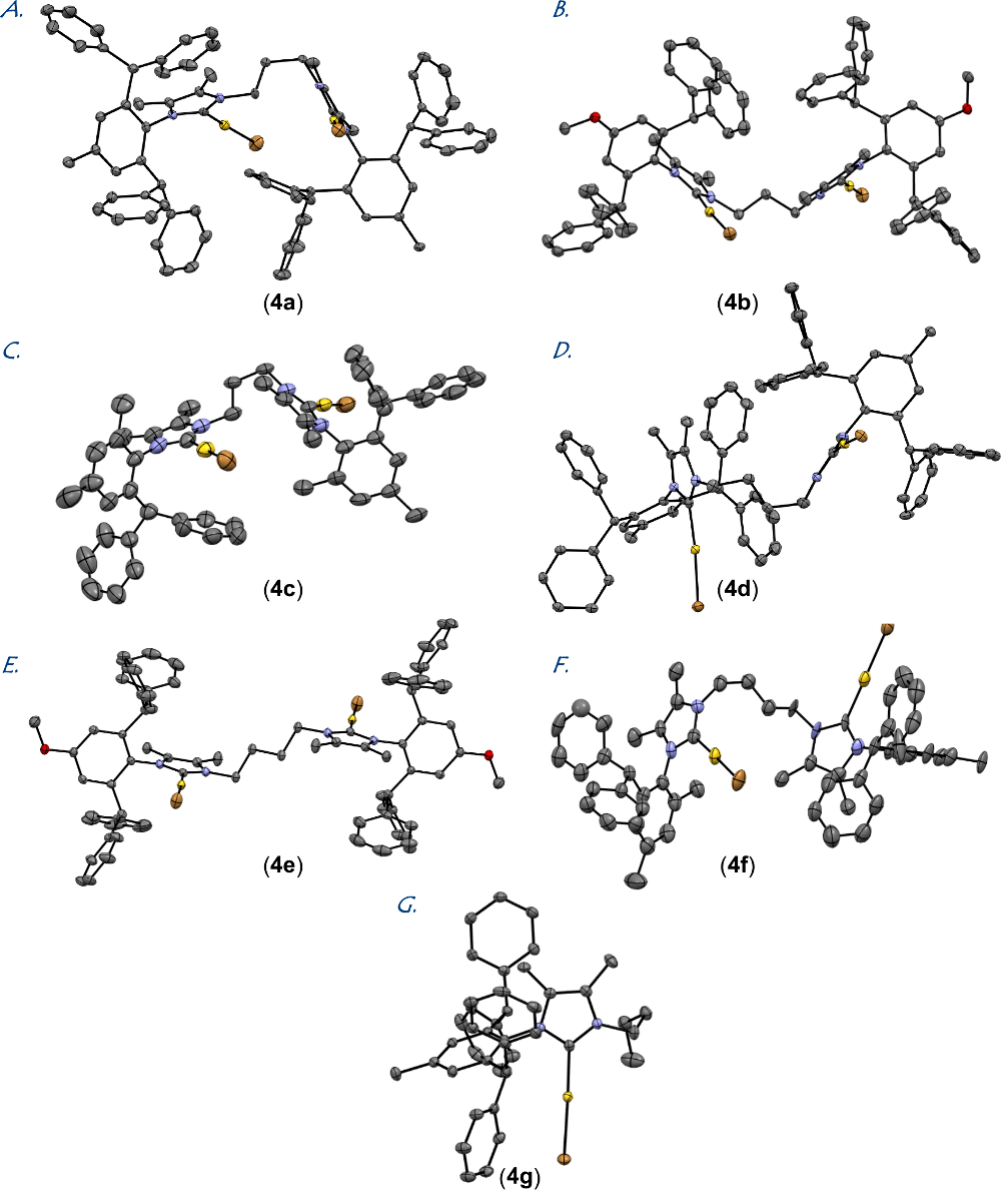
X-ray crystal structures of complexes **4a–4g**. (A) CCDC 2410857, **4a**; (B) CCDC 2410860, **4b**; (C) CCDC 2410859, **4c**; (D) CCDC 2410861, **4d**; (E) CCDC 2410862, **4e**; (F) CCDC 2410858, **4f**; and (G) CCDC 2410863, **4g**. See SI for the selected bond lengths and angles.

**Table 1:** Summary of Steric Parameters of Dinuclear Complexes **4a**–**4f**^a^

NHC–Au	%V_bur_(Au)	NHC–NHC (deg)	Au1–C1–C2–Au2 (deg)	Au1–Au2 (Å)	N–N′ (Å)
**4a**–Au1	50.8	77.2	152.3	6.099	4.326
**4a**–Au2	60.4				
**4b**–Au1	36.6	79.3	146.2	7.859	4.887
**4b**–Au2	42.0				
**4c**–Au1	35.7	58.1	157.9	6.637	4.314
**4c**–Au2	52.2				
**4d**–Au1	48.6	50.2	94.1	8.356	5.083
**4d**–Au2	41.4				
**4e**–Au1	40.2	3.2	180.0	9.112	6.223
**4e**–Au2	40.2				
**4f**–Au1	33.9	41.4	113.6	8.128	5.499
**4f**–Au2	39.9				

a See SI for additional details.

As shown in Figure 2, complexes **4a–4f** were found to be monomeric. All dinuclear complexes **4a–4f** are characterized by linear coordination around gold, which ranges from C–Au–Br of 172.1° (**4a**–Au2) to C–Au–Br of 178.8° (**4c**–Au1). The bond lengths range from C–Au of 1.890 Å (**4f**–Au2) to C–Au of 2.043 Å (**4f**–Au1). This can be compared with the model mononuclear complex **4g**, [Au(IPr*^n-Bu^)Br], C–Au–Br, 175.1°; C–Au, 1.991 Å; Au–Br, 2.392 Å; as well as the parent symmetrical IPr* complex, [Au(IPr*)Cl], C–Au–Cl, 178.4°; C–Au, 1.987 Å; Au–Cl, 2.273 Å.

The bimetallic distances range from 6.099 Å (**4a**–Au1—**4a**–Au2) to 9.112 Å (**4e**–Au1—**4e**–Au2), with the remaining complexes characterized by the bimetallic distances in the following order: 6.637 Å (**4c**–Au1—**4c**–Au2) < 7.859 Å (**4b**–Au1—**4b**–Au2) < 8.128 Å (**4f**–Au1—**4f**–Au2) < 8.356 Å (**4d**–Au1—**4d**–Au2) (Table 1 and Supporting Information (SI)). The distances between the carbenic atoms are in the range from 5.334 Å (**4a**) to 7.928 Å (**4e**) with the remaining complexes in the following order: 5.426 Å (**4c**) < 6.494 Å (**4b**) < 6.562 Å (**4d**) < 6.874 Å (**4f**) < 7.928 Å (**4e**). Indicatives are also the distances between the proximal N atoms of the complexes **4a**–4f with the shortest distances for the complexes **4a** (4.326 Å) and **4c** (4.314 Å) and the longest for **4e** (6.223 Å), while **4b** (4.887 Å), **4d** (5.083 Å) and **4f** (5.499 Å) show intermediate values. Likewise, the distances between the distal N atoms are in the following order: 7.177 Å (**4c**) < 7.214 Å (**4a**) < 7.555 Å (**4d**) < 8.128 Å (**4f**) < 8.137 Å (**4b**) < 10.068 Å (**4e**).

Most interestingly, the dinuclear complexes **4a**–**4f** are distinguished by different conformation of the bridging N-heterocyclic donors with the conformation ranging from the perfectly linear in **4e** (3.2° in a linear-anti arrangement between the Au1–Au2 centers) to almost fully perpendicular in **4b** (79.3° in a linear-anti arrangement between Au1–Au2 centers) between the carbene donors. The remaining complexes are characterized by the conformation of 77.2° (**4a** in bent-anti arrangement) > 58.1° (**4c** in a bent-anti arrangement) > 50.2° (**4d** in a bent syn arrangement) > 41.4° (**4f** in a bent-anti arrangement). This spatial characteristic is visually represented in Figure 3. The corresponding dihedral Au1–C_(carbene)_–C_(carbene’)_–Au2 angles are in the following order: 94.1° (**4d**) < 113.6° (**4f**) < 146.2° (**4b**) < 152.32° (**4a**) < 157.9° (**4c**) < 180.0° (**4e**).

**Figure 3:**
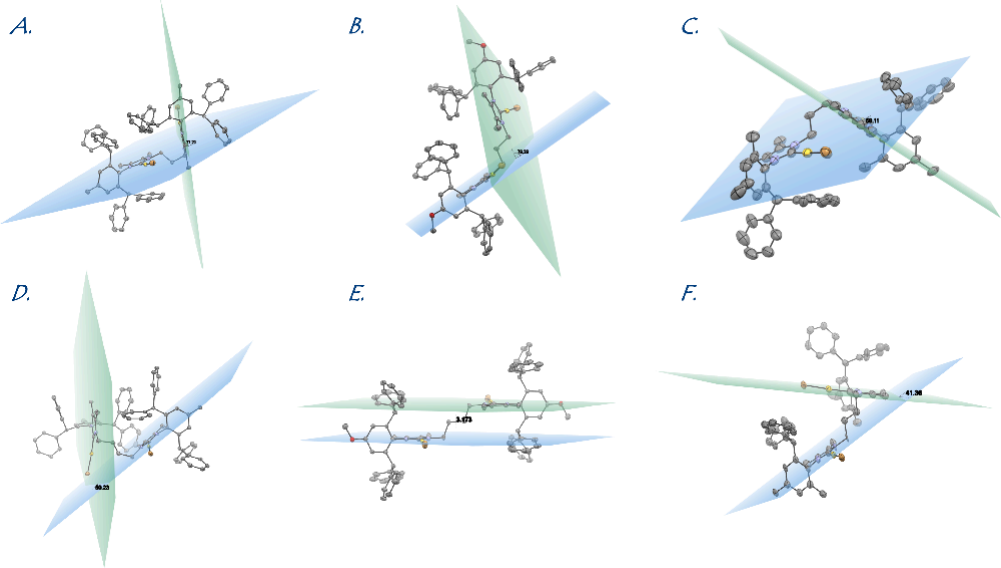
Graphical representation of conformation of dinuclear complexes **4a–4f** with planes drawn between the carbene donors. (A) **4a**, 77.2°; (B) **4b**, 79.3°; (C) **4c**, 58.1°; (D) **4d**, 50.2°; (E) **4e**, 3.2°; (F) **4f**, 41.4°. Note, bent-anti, linear-anti, bent-anti, bent-syn, linear-anti, and bent-anti conformation.

The geometry of binuclear complexes **4a–4f** was further analyzed by the buried volume method pioneered by Díez-González and Nolan (Chart 1).^10a–10c^ The analysis revealed a remarkable range of steric environment around the metal center (%V_bur_ of 33.9 up to 60.4%) (see SI). Thus, it is evident that both the N-aromatic wingtip and the bridging group in the dinuclear complexes **4a**–**4f** impact the steric environment with the key feature as the rotatable substitution rendering the range of geometries around the metal centers covered by dinuclear complexes **4a**–**4f** that are not easily available to mononuclear or less sterically adaptable dinuclear congeners.

**Chart 1:**
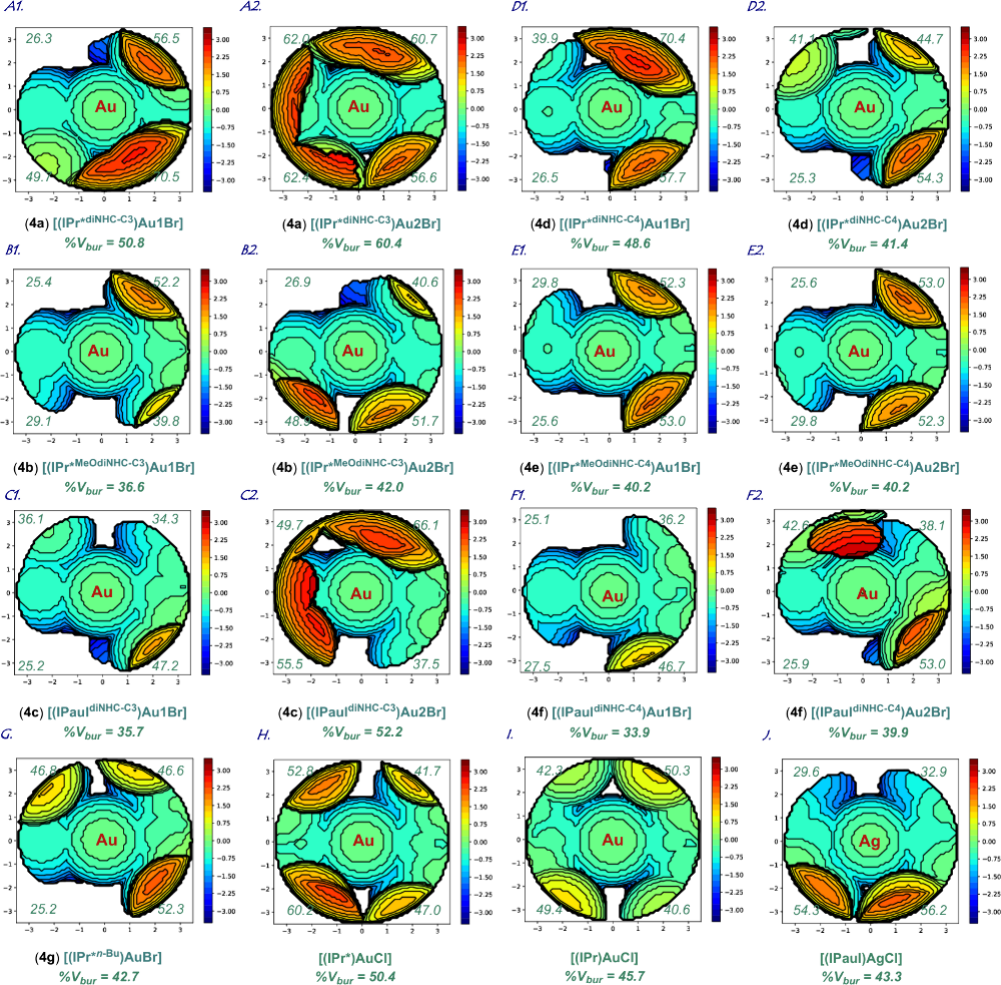
Topographical Steric Maps of Dinuclear Complexes **4a**–**4f** (A–F) Showing %V_bur_ per Quadrant; (G) [(IPr*^n-Bu^)AgCl] (**4g**), (H) [(IPr*)AuCl], (I) [(IPr)AuCl], and (J) [(IPaul)AgCl] for Comparison

In light of the unique features of dinuclear complexes **4a**–**4f**, we were intrigued to test their activity in catalysis. Gold(I)-catalyzed hydration^17^ and carboxylation^18^ of alkynes have been selected as model test reactions due to the industrial importance of alkyne-π functionalization by incorporation of small molecules, H_2_O and CO_2_. Dinuclear gold(I) complexes have emerged as one of the most attractive areas of homogeneous catalysis with further applications in medicinal and materials chemistry. As shown in Scheme 4, the most sterically flexible complex **4c** [Au(IPaul*^diNHC–C3^)Br] was the most active in the carboxylation (84%), which was closely followed by **4f** [Au(IPaul*^diNHC–C4^)Br] (80%) and **4d** [Au(IPr*^diNHC–C4^)Br] (77%), with the remaining complexes in the range of 46–59%. All dinuclear complexes outperformed the model mononuclear complex **4g** [Au(IPr*^n-Bu^)Br] (32%). Furthermore, the more sterically hindered and conformationally flexible dinuclear complex **4d** [Au(IPr*^diNHC–C4^)Br] was the most reactive in the model hydration (78%), which was closely followed by electron-rich **4e** [Au(IPr*^MeOdiNHC–C4^)Br] (66%) and **4b** [Au(IPr*^MeOdiNHC–C3^)Br] (65%), with the remaining complexes in the range of 44–24%. Again, all dinuclear complexes superseded the model mononuclear complex **4g** [Au(IPr*^n-Bu^)Br] (13%). Thus, dinuclear IPr*^diNHC^–Au complexes show a very significant enhancement of catalytic reactivity over the mononuclear gold(I) complex. The high activity of **4a**–**4e** in the synthetically important π-activation of alkynes bodes well for future applications of this sterically adaptable dinuclear catalysis platform in a range of valuable transformations.

**Scheme 4:**
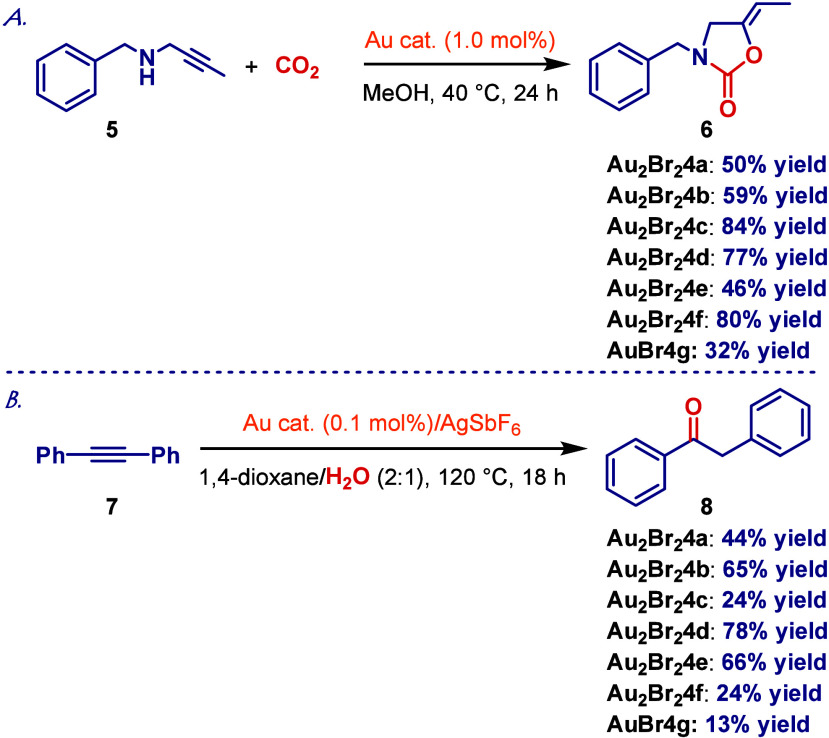
Catalytic Activity of Dinuclear Au(I)–NHC Complexes **4a**–**4f**

To gain insight into the electronic properties of these dinuclear IPr*^diNHC^ ligands, we determined HOMO and LUMO energy levels at the B3LYP6-311++g(d,p) level (Figure 4 and SI). The parent IPr*^diNHC^ carbenes (**3a** and **3b**) and the mononuclear IPr*^n-Bu^ carbene (**3g**) were used as model systems. The σ-donor orbital of IPr*^diNHC–C3^ (**3a**; HOMO–1 due to required symmetry, −5.92 eV, linear-anti geometry, 75.7° conformation) is in the same range as the σ-donor orbital of IPr*^diNHC–C4^ (**3b**; −5.90 eV, linear-anti geometry, 0.0° conformation) and the mononuclear IPr*^n-Bu^ (**3g**; −5.85 eV). These values can be compared with those of IPr* (−6.12 eV) and classical IPr (−6.01 eV). The results indicate that (1) IPr*^diNHC^ carbenes are electronically characterized as strongly σ-nucleophilic ligands and (2) the steric impact arises from the dinuclear rotationally adaptable imidazol-2-ylidene framework.

**Figure 4:**
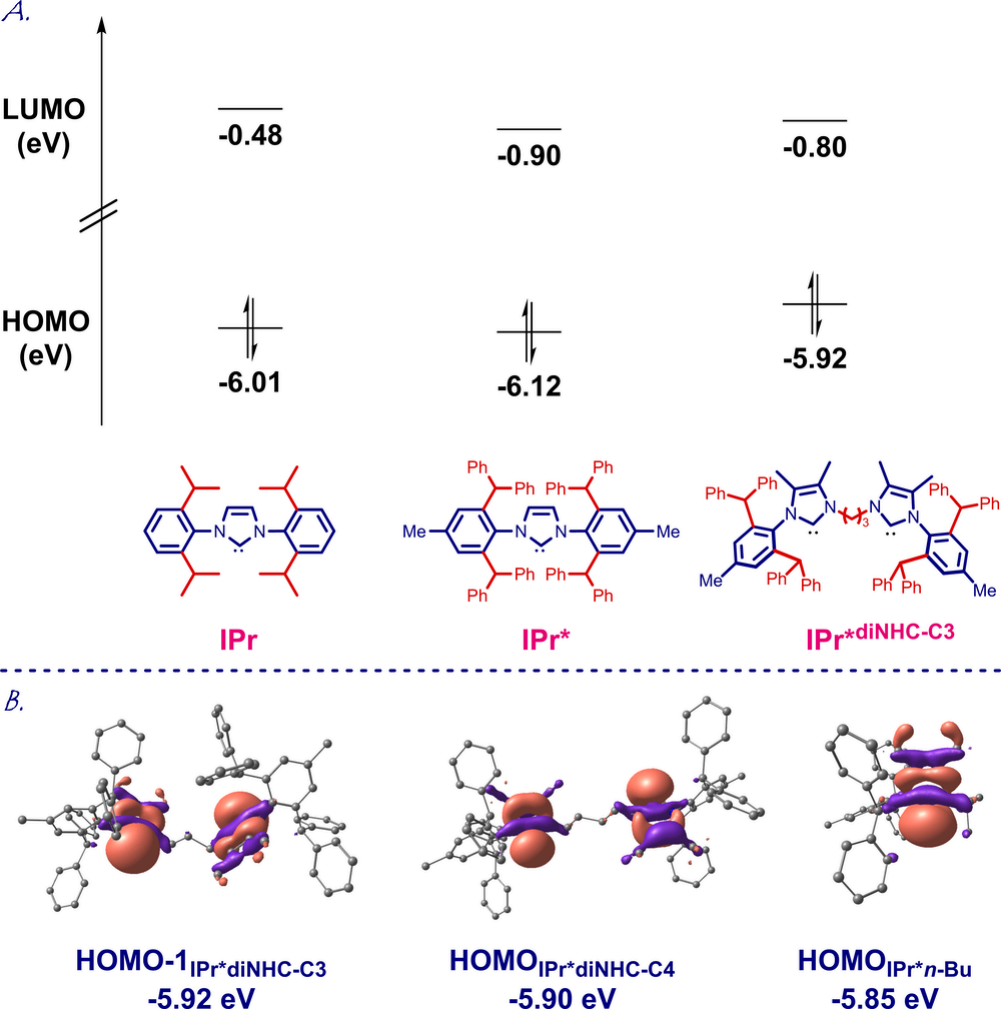
(A) HOMO and LUMO energies (eV). (B) σ-Donor orbitals of IPr*^diNHC-C3^, IPr*^diNHC-C4^, and IPr*^n-Bu^ (eV) calculated at B3LYP 6-311++g(d,p). See SI.

In summary, we have reported a new class of dinuclear sterically hindered and adaptable N-heterocyclic ligands based on the modular IPr* framework. The key features of this class of ligands are a remarkably broadly ranging steric environment around the metal center and adaptable conformation ranging from perfectly linear to almost fully perpendicular between the carbene donors. These features arise from the sterically hindered but flexible environment engendered by the ligand framework. Considering the importance of dinuclear complexes in diverse areas of chemical science, we anticipate broad applications across many fields that utilize sterically demanding carbenes as ancillary ligands.

## Supporting Information Available

Experimental section, crystallographic section, additional studies, computational methods, ^1^H and ^13^C NMR spectra, and Cartesian coordinates with zero-point energies and thermal corrections (PDF)

## References

[ref1] aArduengo A. J.; Harlow R. L.; Kline M. A stable crystalline carbene. J. Am. Chem. Soc. 1991, 113, 361–363. 10.1021/ja00001a054.

[ref2] aHerrmann W. A. N-Heterocyclic Carbenes: A New Concept in Organometallic Catalysis. Angew. Chem. Int. Ed. 2002, 41, 1290–1309. 10.1002/1521-3773(20020415)41:8<1290::AID-ANIE1290>3.0.CO;2-Y. 19750753

[ref3] aBoydston A. J.; Williams K. A.; Bielawski C. W. A modular approach to main-chain organometallic polymers. J. Am. Chem. Soc. 2005, 127, 12496–12497. 10.1021/ja054029k. 16144390

[ref4] aNavarro O.; Kelly R. A.; Nolan S. P. A general method for the Suzuki-Miyaura cross-coupling of sterically hindered aryl chlorides: synthesis of di- and tri-ortho-substituted biaryls in 2-propanol at room temperature. J. Am. Chem. Soc. 2003, 125, 16194–16195. 10.1021/ja038631r. 14692753

[ref5] aAltenhoff G.; Goddard R.; Lehmann C. W.; Glorius F. Sterically demanding, bioxazoline-derived N-heterocyclic carbene ligands with restricted flexibility for catalysis. J. Am. Chem. Soc. 2004, 126, 15195–15201. 10.1021/ja045349r. 15548016

[ref6] aLavallo V.; Canac Y.; Präsang C.; Donnadieu B.; Bertrand G. Stable Cyclic (Alkyl)(Amino)Carbenes as Rigid or Flexible, Bulky, Electron-Rich Ligands for Transition-Metal Catalysts: A Quaternary Carbon Atom Makes the Difference. Angew. Chem. Int. Ed. 2005, 44, 5705–5709. 10.1002/anie.200501841. PMC2427276 16059961

[ref7] aBerthon-Gelloz G.; Siegler M. A.; Spek A. L.; Tinant B.; Reek J. N. H.; Markó I. E. IPr* an easily accessible highly hindered N-heterocyclic carbene. Dalton Trans. 2010, 39, 1444–1446. 10.1039/B921894G. 20104298

[ref8] aGründemann S.; Albrecht M.; Kovacevic A.; Faller J. W.; Crabtree R. H. Bis-carbene complexes from oxidative addition of imidazolium C–H bonds to palladium(0). Dalton Trans. 2002, 2163–2167. 10.1039/b110964b.

[ref9] aDragutan V.; Dragutan I.; Delaude L.; Demonceau A. NHC–Ru complexes—Friendly catalytic tools for manifold chemical transformations. Coord. Chem. Rev. 2007, 251, 765–794. 10.1016/j.ccr.2006.09.002.

[ref10] aHillier A. C.; Sommer W. J.; Yong B. S.; Petersen J. L.; Cavallo L.; Nolan S. P. A Combined Experimental and Theoretical Study Examining the Binding of N-heterocyclic Carbenes (NHC) to the Cp*RuCl (Cp* = η5-C5Me5) Moiety: Insight into Stereoelectronic Differences between Unsaturated and Saturated NHC Ligands. Organometallics 2003, 22, 4322–4326. 10.1021/om034016k.

[ref11] aKuriyama M.; Hamaguchi N.; Yano G.; Tsukuda K.; Sato K.; Onomura O. Deuterodechlorination of Aryl/Heteroaryl Chlorides Catalyzed by a Palladium/Unsymmetrical NHC System. J. Org. Chem. 2016, 81, 8934–8946. 10.1021/acs.joc.6b01609. 27641511

[ref12] aMeiries S.; Speck K.; Cordes D. B.; Slawin A. M. Z.; Nolan S. P. [Pd(IPr*OMe)(acac)Cl]: Tuning the N-heterocyclic Carbene in Catalytic C–N Bond Formation. Organometallics 2013, 32, 330–339. 10.1021/om3011867.

[ref13] aScherg T.; Schneider S.; Frey G.; Schwarz J.; Herdtweck E.; Herrmann W. Bridged Imidazolium Salts Used as Precursors for Chelating Carbene Complexes of Palladium in the Mizoroki-Heck Reaction. Synlett 2006, 2006, 2894–2907. 10.1055/s-2006-951539.

[ref14] aAhrens S.; Peritz A.; Strassner T. Tunable aryl alkyl ionic liquids (TAAILs): the next generation of ionic liquids. Angew. Chem. Int. Ed. 2009, 48, 7908–7910. 10.1002/anie.200903399. 19760688

[ref15] Baron M.; Battistel E.; Tubaro C.; Biffis A.; Armelao L.; Rancan M.; Graiff C. Single-Step Synthesis of Dinuclear Neutral Gold(I) Complexes with Bridging Di(N-heterocyclic carbene) Ligands and Their Catalytic Performance in Cross Coupling Reactions and Alkyne Hydroamination. Organometallics 2018, 37, 4213–4223. 10.1021/acs.organomet.8b00531.

[ref17] aMarion N.; Ramón R. S.; Nolan S. P. [(NHC)Au(I)]-catalyzed acid-free alkyne hydration at part-per-million catalyst loadings. J. Am. Chem. Soc. 2009, 131, 448–449. 10.1021/ja809403e. 19140786

[ref18] aKayaki Y.; Yamamoto M.; Suzuki T.; Ikariya T. Carboxylative cyclization of propargylamines with supercritical carbon dioxide. Green Chem. 2006, 8, 1019 10.1039/b603700c.

