# Supplementary material for: IPr*diNHC: Sterically Adaptable Dinuclear N-Heterocyclic Carbenes
Source: Inorg Chem. 2025 Apr 15;64(16):7851–7. doi: 10.1021/acs.inorgchem.5c01013 (PMC12042265; doi:10.1021/acs.inorgchem.5c01013)
Supplement: Supplementary file 1 — ic5c01013_si_001.pdf [file ic5c01013_si_001.pdf]

Supporting Information

IPr*diNHC – Sterically-Adaptable Dinuclear N-Heterocyclic Carbenes

Katarzyna Halikowska-Tarasek,[†] Wioletta Ochędzan-Siodłak,[†] Błażej Dziuk,[◊] Roman Szostak,[Δ] Michal Szostak*[,§] and Elwira Bisz*[,†]

[†]Department of Chemistry and Pharmacy, Opole University, 48 Oleska Street, Opole 45-052, Poland
[◊]Department of Chemistry, Wroclaw University of Science and Technology, Norwida 4/6, Wroclaw 50-373, Poland
[Δ]Department of Chemistry, Wroclaw University, F. Joliot-Curie 14, Wroclaw 50-383, Poland
[§]Department of Chemistry, Rutgers University, 73 Warren Street, Newark, NJ 07102, United States

ebisz@uni.opole.pl, michal.szostak@rutgers.edu

Table of Contents

Experimental section....................2
Crystallographic Studies....................9
Additional Studies Referred to from the Main Manuscript....................69
Computational Methods....................73
^{1}H and ^{13}C NMR Spectra....................75
Cartesian Coordinates with Zero-Point Energies and Thermal Corrections....................97

Experimental section

General methods

All experiments were performed using standard Schlenk techniques under argon unless stated otherwise. All solvents were purchased at the highest commercial grade and were deoxygenated prior to use. All other chemicals were purchased at the highest commercial grade and used as received. Compounds 1a[1a], 1b[1d], 1c[1f,1g], 2a[2a-2c] and 2b[2b] have been previously reported in the literature. Spectroscopic properties matched the literature data. ^{1}H NMR and ^{13}C NMR spectra were recorded on Bruker spectrometers at 400 (^{1}H NMR) and 100 MHz (^{13}C NMR). High-resolution mass spectroscopy (HRMS) and elemental analyses were performed on a 7T Bruker Daltonics FT-MS and Vario EL II(CHNS) instrument respectively.

Synthesis of Anilines

Anilines were synthesized according to literature procedures.[1]

2,6-Dibenzhydryl-4-methylaniline (1a).[1a] The product was obtained in 52% yield (24.00 g, 54.60 mmol) as a white solid. ^{1}H NMR (400 MHz, $CDCl_3$) δ 7.29 – 7.26 (m, 8H), 7.24 – 7.19 (m, 4H), 7.09 (d, J = 7.0 Hz, 8H), 6.38 (s, 2H), 5.45 (s, 2H), 3.27 (s, 2H), 2.01 (s, 3H). ^{13}C NMR (101 MHz, $CDCl_3$) δ 142.90, 139.78, 129.69, 129.38, 129.18, 128.63, 126.79, 126.74, 52.51, 21.17.

4-Methoxy-2,6-bis(diphenylmethyl)aniline (1b).[1d] The product was obtained in 93% yield (44.00 g, 96.58 mmol) as a white solid. ^{1}H NMR (400 MHz, $CDCl_3$) δ 7.27 (t, J = 7.3 Hz, 8H), 7.20 (t, J = 7.2 Hz, 4H), 7.09 (d, J = 7.2 Hz, 8H), 6.19 (s, 2H), 5.47 (s, 2H), 3.41 (s, 3H), 3.13 (s, 2H). ^{13}C NMR (101 MHz, $CDCl_3$) δ 151.93, 142.63, 136.03, 130.95, 129.65, 128.68, 126.86, 114.49, 55.26, 52.61.

2-Benzhydryl-4,6-dimethylaniline (1c).[1f,1g] The product was obtained in 73% yield (22.00 g, 76.55 mmol) as a white solid. ^{1}H NMR (400 MHz, $CDCl_3$) δ 7.28 (t, J = 7.2 Hz, 4H), 7.23 – 7.19 (m, 2H), 7.12 (d, J = 7.0 Hz, 4H), 6.81 (s, 1H), 6.34 (s, 1H), 5.47 (s, 1H), 3.32 (s, 2H), 2.11 (d, J = 4.3 Hz, 6H). ^{13}C NMR (101 MHz, $CDCl_3$) δ 142.91, 139.99, 129.70, 129.65, 128.73, 128.65, 128.53, 126.99, 126.70, 122.72, 52.53, 20.85, 17.86.

Synthesis of 1-Arylimidazoles

N-Aryl imidazoles were synthesized according to a modified literature procedure.[2] To an aniline derivative (12.00 mmol) in dry $CHCl_3$ (20.00 mL), diacetyl (10.00 mmol), acetic acid (50.00 mmol), NH_4OAc (12.00 mmol), paraformaldehyde (10.00 mmol), and H_2O (0.50 mL) were added, and the mixture was refluxed for 48 h. After removal of the solvent, the dark residue was dissolved in Et_2O and basified to pH 14 in an ice bath with aqueous 40% KOH solution. The resulting mixture was extracted with Et_2O, and the combined organic layers were washed with H_2O and dried over Na_2SO_4. Concentration and purification through silica gel column chromatography gave the desired product.

1-(2,6-Dibenzhydryl-4-methylphenyl)-4,5-dimethyl-1*H*-imidazole (2a). The product was obtained in 50% yield (2.00 g, 3.85 mmol) as a pale brown solid. Purification by flash chromatography (hexane/AcOEt = 2/1). ^{1}H NMR (400 MHz, $CDCl_3$) δ 7.23 – 7.16 (m, 12H), 6.96 (d, J = 7.1 Hz, 4H), 6.89 (d, J = 7.1 Hz, 4H), 6.87 (s, 2H), 6.62 (s, 1H), 5.00 (s, 2H), 2.25 (s, 3H), 2.16 (s, 3H), 1.30 (s, 3H). ^{13}C NMR (101 MHz, $CDCl_3$) δ 142.88, 142.44, 138.96, 135.63, 133.76, 132.31, 129.63, 129.26, 128.55, 128.39, 126.69, 123.54, 51.53, 21.95, 12.96, 7.90.

1-(2,6-Dibenzhydryl-4-methoxyphenyl)-4,5-dimethyl-1*H*-imidazole (2b). The product was obtained in 54% yield (2.17 g, 4.06 mmol) as a pale yellow solid. Purification by flash chromatography (hexane/AcOEt = 2/1). ^{1}H NMR (400 MHz, $CDCl_3$) δ 7.25 – 7.17 (m, 12H), 6.96 (d, J = 6.8 Hz, 4H), 6.90 (d, J = 6.9 Hz, 4H), 6.57 (s, 3H), 5.00 (s, 2H), 3.61 (s, 3H), 2.15 (s, 3H), 1.30 (s, 3H). ^{13}C NMR (101 MHz, $CDCl_3$) δ 159.39, 144.61, 142.63, 142.21, 135.95, 129.58, 129.18, 128.57, 128.41, 127.83, 126.76, 123.55, 114.44, 55.38, 51.75, 13.09, 8.03.

1-(2-benzhydryl-4,6-dimethylphenyl)-4,5-dimethyl-1H-imidazole (2c). The product was obtained in 70% yield (3.50 g, 9.54 mmol) as a pale brown solid. Purification by flash chromatography (hexane/AcOEt = 2/1). ^{1}H NMR (400 MHz, $CDCl_3$) δ 7.26 – 7.16 (m, 6H), 7.01 (s, 1H), 6.95 (d, J = 7.8 Hz, 4H), 6.92 (s, 1H), 6.80 (s, 1H), 5.09 (s, 1H), 2.29 (s, 3H), 2.20 (s, 3H), 1.89 (s, 3H), 1.59 (s, 3H). ^{13}C NMR (101 MHz, $CDCl_3$) δ 142.86, 142.65, 142.40, 138.83, 136.66, 135.01, 133.82, 132.43, 129.79, 129.55, 129.29, 128.87, 128.50, 128.31, 126.61, 126.56, 123.05, 51.45, 21.54, 17.61, 13.07, 8.09.

Synthesis of Imidazolium Salts

Imidazolium salts were synthesized according to a modified literature procedure.[3] To a solution of imidazole 2a-2c (0.56 mmol) in acetonitrile (0.56 mL) in a closed pressure tube, $C_3H_6Cl_2$ or $C_4H_8Cl_2$ (0.28 mmol) was added. A colorless precipitate formed after the solution had been heated at 110 °C for 3 days. The precipitate was filtered and washed with diethyl ether until the supernatant was colorless. The products (**3a-3f**) then dried in vacuo to give the desired bis(imidazolium) salt.

3,3'-(Propane-1,3-diyl)bis(1-(2,6-dibenzhydryl-4-methylphenyl)-4,5-dimethyl-1H-imidazol-3-ium) Dichloride (3a). New compound. The product was obtained in 88% yield (0.29 g, 0.25 mmol) as beige powder of mp 267 – 270 °C. ^{1}H NMR (400 MHz, $CDCl_3$) δ 9.89 (s, 2H), 7.28 – 7.23 (m, 10H), 7.20 – 7.14 (m, 14H), 7.10 (d, J = 7.4 Hz, 8H), 6.90 (d, J = 6.9 Hz, 8H), 6.73 (s, 4H), 5.15 (s, 4H), 4.38 (t, J = 7.5 Hz, 4H), 2.69 (s, 2H), 2.39 (s, 6H), 2.20 (s, 6H), 1.06 (s, 6H). ^{13}C NMR (101 MHz, $CDCl_3$) δ 142.16, 140.93, 140.79, 137.29, 130.27, 129.42, 129.37, 128.86, 128.74, 127.40, 127.19, 127.09, 52.27, 44.34, 21.91, 15.40, 9.26, 7.59. HRMS (ESI/Q-TOF)_m/z (%) [M-H]$^+$ calcd for $C_{79}H_{73}N_4$ 1077.5835 found 1077.5906.

3,3'-(Propane-1,3-diyl)bis(1-(2,6-dibenzhydryl-4-methoxyphenyl)-4,5-dimethyl-1H-imidazol-3-ium) Dichloride (3b). New compound. The product was obtained in 78% yield (0.26 g, 0.22 mmol) as beige powder of mp 215 – 218 °C. ^{1}H NMR (400 MHz, $CDCl_3$) δ 9.90 (s, 2H), 7.28 – 7.18 (m, 24H), 7.13 (d, J = 7.8 Hz, 8H), 6.92 (d, J = 7.0 Hz, 8H), 6.43 (s, 4H), 5.18 (s, 4H), 4.36 (s, 4H), 3.53 (s, 6H), 2.73 (s, 2H), 2.38 (s, 6H), 1.09 (s, 6H). ^{13}C NMR (101 MHz, $CDCl_3$) 160.47, 144.08, 140.61, 140.52, 137.51, 129.29, 129.24, 128.82, 128.69, 127.20, 127.14, 115.08, 55.28, 52.38, 44.42, 44.26, 41.72, 32.89, 30.44, 9.17, 7.54. HRMS (ESI/Q-TOF) m/z (%) [M-H]$^+$ calcd for $C_{79}H_{73}N_4O_2$ 1109.5734 found 1109.5854.

3,3'-(Propane-1,3-diyl)bis(1-(2-benzhydryl-4,6-dimethylphenyl)-4,5-dimethyl-1H-imidazol-3-ium) Dichloride (3c). New compound. The product was obtained in 86% yield (0.20 g, 0.24 mmol) as beige powder of mp 277 – 280 °C. ^{1}H NMR (400 MHz, $CDCl_3$) δ 10.16 (d, J = 8.8 Hz, 2H), 7.29 – 7.24 (m, 6H), 7.22 – 7.18 (m, 6H), 7.12 – 7.09 (m, 4H), 7.05 (s, 2H), 6.89 (t, J = 7.0 Hz, 4H), 6.68 (s, 2H), 5.22 (d, J = 14.3 Hz, 2H), 4.75 – 4.61 (m, 4H), 2.84 (d, J = 33.0 Hz, 2H), 2.51 (d, J = 5.8 Hz, 6H), 2.28 (s, 6H), 1.96 (d, J = 8.6 Hz, 6H), 1.42 (d, J = 12.1 Hz, 6H). ^{13}C NMR (101 MHz, $CDCl_3$) δ 141.42, 141.14, 140.86, 136.36, 135.64, 130.62, 129.42, 129.07, 128.81, 128.69, 128.05, 127.19, 127.03, 52.13, 52.03, 44.35, 31.63, 21.56, 17.83, 9.36, 7.88, 7.82. HRMS (ESI/Q-TOF)_m/z (%) [M-H]$^+$ calcd for $C_{55}H_{57}N_4$ 773.4583 found 773.4619.

3,3'-(Butane-1,4-diyl)bis(1-(2,6-dibenzhydryl-4-methylphenyl)-4,5-dimethyl-1H-imidazol-3-ium) Dichloride (3d). *New compound.* The product was obtained in 76% yield (0.24 g, 0.21 mmol) as beige powder of mp 288 – 291 °C. ^{1}H NMR (400 MHz, $CDCl_3$) δ 9.76 (s, 2H), 7.32 (t, J = 7.4 Hz, 8H), 7.25 – 7.22 (m, 16H), 7.09 (d, J = 7.3 Hz, 8H), 6.91 (d, J = 6.7 Hz, 8H), 6.76 (s, 4H), 5.11 (s, 4H), 4.30 (s, 4H), 2.23 (d, J = 14.2 Hz, 12H), 2.00 (s, 4H), 1.07 (s, 6H). ^{13}C NMR (101 MHz, $CDCl_3$) δ 142.08, 141.11, 141.05, 140.89, 136.99, 130.42, 129.51, 129.37, 128.96, 128.76, 127.43, 127.23, 127.18, 126.88, 52.17, 46.67, 27.05, 21.94, 8.94, 7.58. HRMS (ESI/Q-TOF) m/z (%) $[M-H]^+$ calcd for $C_{80}H_{75}N_4$ 1091.5992 found 1091.6101.

3,3'-(Butane-1,4-diyl)bis(1-(2,6-dibenzhydryl-4-methoxyphenyl)-4,5-dimethyl-1H-imidazol-3-ium) Dichloride (3e). *New compound.* The product was obtained in 81% yield (0.28 g, 0.23 mmol) as beige powder of mp 249 – 253 °C. ^{1}H NMR (400 MHz, $CDCl_3$) δ 9.64 (s, 2H), 7.32 (t, J = 7.4 Hz, 8H), 7.25 – 7.22 (m, 16H), 7.10 (d, J = 7.4 Hz, 8H), 6.93 (d, J = 6.8 Hz, 8H), 6.46 (s, 4H), 5.12 (s, 4H), 4.26 (s, 4H), 3.55 (s, 6H), 2.25 (s, 6H), 1.99 (d, J = 10.4 Hz, 4H), 1.11 (s, 6H). ^{13}C NMR (101 MHz, $CDCl_3$) δ 160.65, 144.09, 140.87, 140.73, 137.35, 129.47, 129.34, 129.02, 128.80, 127.33, 115.45, 115.31, 55.40, 52.40, 46.67, 27.03, 8.98, 7.64. HRMS (ESI/Q-TOF) m/z (%) $[M-H]^+$ calcd for $C_{80}H_{75}N_4O_2$ 1123.5890 found 1123.5975.

3,3'-(Butane-1,4-diyl)bis(1-(2-benzhydryl-4,6-dimethylphenyl)-4,5-dimethyl-1H-imidazol-3-ium) Dichloride (3f). *New compound.* The product was obtained in 75% yield (0.18 g, 0.21 mmol) as beige powder of mp 284 – 287 °C. ^{1}H NMR (400 MHz, $CDCl_3$) δ 10.11 – 9.98 (m, 2H), 7.29 (s, 3H), 7.24 (d, J = 7.4 Hz, 7H), 7.12 – 7.08 (m, 4H), 7.02 (s, 2H), 6.94 (d, *J* = 6.0 Hz, 3H), 6.90 (d, *J* = 8.1 Hz, 3H), 6.67 (s, 2H), 5.25 – 5.17 (m, 2H), 4.58 (d, *J* = 17.6 Hz, 4H), 2.53 (s, 4H), 2.36 (d, J = 6.7 Hz, 3H), 2.29 (d, J = 7.8 Hz, 6H), 2.22 (d, J = 8.3 Hz, 6H), 1.98 (s, 1H), 1.89 (d, J = 4.0 Hz, 3H), 1.59 (s, 1H), 1.48 (s, 1H), 1.39 (d, J = 6.2 Hz, 3H). ^{13}C NMR δ 141.42, 141.16, 141.09, 140.78, 140.67, 136.20, 135.55, 130.59, 129.48, 129.43, 129.39, 129.34, 128.80, 128.74, 128.67, 127.58, 127.53, 127.24, 127.19, 127.02, 52.05, 46.73, 26.75, 21.55, 17.74, 17.68, 9.05, 9.01, 7.77, 7.73. HRMS (ESI/Q-TOF) m/z (%) $[M-H]^+$ calcd for $C_{56}H_{59}N_4$ 787.4740 found 787.4750.

Procedure for the Synthesis of Imidazolium Salt 3g. Imidazolium salt was synthesized according to a modified literature procedure.[4] To a solution of imidazole 2a (1.54 mmol) in tetrahydrofuran (1.30 mL) in a closed pressure tube, C_4H_9Br (1.69 mmol) was added. A white precipitate formed after the solution had been heated at 100 °C for 3 days. The precipitate was filtered and washed with tetrahydrofuran until the supernatant was colorless. The product (**3g**) then dried in vacuo to give the desired imidazolium salt.

3-Butyl-1-(2,6-dibenzhydryl-4-methylphenyl)-4,5-dimethyl-1H-imidazol-3-ium Bromide (3g). New compound. The product was obtained in 67% yield (0.68 g, 1.03 mmol) as a white powder of mp 189 – 193 °C. ^{1}H NMR (400 MHz, $CDCl_3$) δ 8.93 (s, 1H), 7.30 (t, J = 7.5 Hz, 4H), 7.25 – 7.19 (m, 8H), 7.07 (d, J = 7.2 Hz, 4H), 6.94 (d, J = 6.3 Hz, 4H), 6.76 (s, 2H), 5.16 (s, 2H), 4.21 (t, J = 7.6 Hz, 2H), 2.20 (d, J = 14.5 Hz, 6H), 1.45 (dt, J = 12.4, 7.6 Hz, 2H), 1.36 (s, 3H), 1.24 (dq, J = 14.7, 7.3 Hz, 2H), 0.91 (t, J = 7.3 Hz, 3H). ^{13}C NMR (101 MHz, $CDCl_3$) δ 141.95, 141.23, 141.04, 136.17, 130.54, 129.47, 129.26, 128.90, 128.63, 127.51, 127.13, 126.38, 51.88, 47.31, 32.04, 21.89, 19.52, 13.72, 8.83, 8.05. HRMS (ESI/Q-TOF) m/z (%) [M]+ calcd for $C_{42}H_{43}N_2$ 575.343 found 575.338.

Synthesis of Dinuclear Au(I)–NHC Complexes

Au(I)–bis(NHC) complexes were synthesized according to a modified literature procedure.[5] A mixture of the imidazolium salt (0.10 mmol), Au(DMS)Cl (0.20 mmol), K_2CO_3 (2.20 mmol), and LiBr (0.50 mmol) in acetonitrile (10.00 mL) was heated and maintained at 60 °C for 3 days. The obtained suspension was then filtered through silica gel. The solvent was removed at reduced pressure from the filtrate, obtaining a brown/beige/yellow/off-white solid. Crystallization by slow diffusion from CH_2Cl_2/Hex at RT gave suitable crystals for X-ray diffraction studies.

Caution: in case of mononuclear Au(I)–NHC, the amounts of particular reagents were as follows: imidazolium salt (0.10 mmol), Au(DMS)Cl (0.10 mmol), K_2CO_3 (1.10 mmol), LiBr (0.25 mmol), acetonitrile (5.00 mL).

$Au_2Br_2$4a. New compound. The product was obtained in 90% yield (0.15 g, 0.09 mmol) as beige solid. ^{1}H NMR (400 MHz, $CDCl_3$) δ 7.26 (s, 2H), 7.21 – 7.17 (m, 14H), 7.14 – 7.12 (m, 4H), 7.10 (d, J = 4.7 Hz, 8H), 7.08 – 7.05 (m, 4H), 6.89 (d, J = 6.5 Hz, 8H), 6.78 (s, 4H), 5.21 (s, 4H), 3.98 (t, J = 7.6 Hz, 4H), 2.22 (s, 6H), 2.18 – 2.15 (m, 2H), 2.11 (s, 6H), 0.96 (s, 6H). ^{13}C NMR (101 MHz, $CDCl_3$) δ 173.51, 142.31, 142.03, 141.58, 139.70, 133.01, 130.45, 129.89, 129.70, 128.68, 128.53, 127.99, 126.91, 126.53, 124.35, 51.49, 45.75, 31.61, 22.00, 9.83, 8.51. Anal. Calcd for $C_{79}H_{72}Au_2Br_2N_4$ (1631.21): C, 58.17; H, 4.45; N, 3.43. Found: C, 58.29; H, 4.51; N, 3.32.

$Au_2Br_2$4b. New compound. The product was obtained in 70% yield (0.12 g, 0.07 mmol) as brown solid. ^{1}H NMR (400 MHz, $CDCl_3$) δ 7.26 (s, 2H), 7.23 – 7.17 (m, 14H), 7.11 (d, J = 6.5 Hz, 12H), 7.07 – 7.04 (m, 4H), 6.90 (d, J = 6.5 Hz, 8H), 6.49 (s, 4H), 5.23 (s, 4H), 3.96 (t, J = 7.7 Hz, 4H), 3.57 (s, 6H), 2.10 (s, 6H), 1.26 (s, 2H), 0.99 (s, 6H). ^{13}C NMR (101 MHz, $CDCl_3$)

δ 173.96, 159.83, 143.90, 142.03, 141.42, 129.84, 129.68, 128.76, 128.58, 128.17, 127.01, 126.63, 115.25, 55.30, 51.76, 45.72, 31.60, 29.88, 9.85, 8.54. Anal. Calc for $C_{79}H_{72}Au_2Br_2N_4O_2$ (1663.21): C, 57.05; H, 4.36; N, 3.37. Found: C, 57.02; H, 4.34; N, 3.25.

$Au_2Br_2$4c. <u>*New compound.*</u> The product was obtained in 60% yield (0.08 g, 0.06 mmol) as beige solid. ^{1}H NMR (400 MHz, $CDCl_3$) δ 7.26 (s, 2H), 7.24 – 7.20 (m, 6H), 7.19 – 7.16 (m, 8H), 6.99 (s, 2H), 6.93 – 6.88 (m, 4H), 6.70 (s, 2H), 5.38 (d, J = 6.4 Hz, 2H), 4.33 (t, J = 7.2 Hz, 2H), 4.16 (t, J = 7.7 Hz, 2H), 2.40 – 2.37 (m, 2H), 2.27 (s, 6H), 2.21 (s, 6H), 1.91 (d, J = 1.4 Hz, 6H), 1.35 (s, 3H), 1.26 (s, 3H). ^{13}C NMR (101 MHz, $CDCl_3$) δ 172.72, 142.44, 141.69, 139.68, 135.88, 133.13, 130.30, 129.91, 129.82, 129.77, 129.66, 129.55, 128.62, 128.52, 127.51, 127.28, 126.90, 126.55, 126.46, 124.51, 51.47, 45.74, 31.91, 21.66, 18.32, 18.27, 9.78, 8.84, 8.71. Anal. Calc for $C_{55}H_{56}Au_2Br_2N_4$ (1326.82): C, 49.79; H, 4.25; N, 4.22. Found: C, 49.63; H, 4.31; N, 4.23.

$Au_2Br_2$4d. <u>*New compound.*</u> The product was obtained in 82% yield (0.13 g, 0.082 mmol) as off-white solid. ^{1}H NMR (400 MHz, $CDCl_3$) δ 7.29 – 7.24 (m, 12H), 7.22 – 7.20 (m, 12H), 7.13 (d, J = 7.3 Hz, 8H), 6.89 (d, J = 6.8 Hz, 8H), 6.78 (s, 4H), 5.23 (s, 4H), 4.13 (s, 4H), 2.24 (s, 6H), 2.05 (s, 6H), 1.86 (s, 4H), 0.69 (s, 6H). ^{13}C NMR (101 MHz, $CDCl_3$) δ 173.41, 142.42, 142.34, 141.24, 139.72, 133.26, 130.35, 129.83, 129.65, 128.74, 128.58, 127.79, 126.99, 126.71, 124.08, 51.61, 48.01, 28.40, 22.01, 9.43, 7.95. Anal. Calc for $C_{80}H_{74}Au_2Br_2N_4$ (1645.24): C, 58.40; H, 4.53; N, 3.41. Found: C, 58.31; H, 4.61; N, 3.29.

$Au_2Br_2$4e. <u>*New compound.*</u> The product was obtained in 67% yield (0.11 g, 0.067 mmol) as brown solid. ^{1}H NMR (400 MHz, $CDCl_3$) δ 7.29 – 7.24 (m, 8H), 7.23 – 7.18 (m, 16H), 7.14 (d, J = 7.3 Hz, 8H), 6.90 (d, J = 6.9 Hz, 8H), 6.49 (s, 4H), 5.25 (s, 4H), 4.12 (s, 4H), 3.59 (s, 6H), 2.05 (s, 6H), 1.84 (s, 4H), 0.72 (s, 6H). ^{13}C NMR (101 MHz, $CDCl_3$) δ 173.79, 159.80, 144.16, 142.08, 141.01, 129.76, 129.60, 128.79, 128.60, 127.89, 127.07, 126.80, 115.10, 55.32, 51.84, 47.94, 29.86, 28.34, 9.43, 7.94. Anal. Calc for $C_{80}H_{74}Au_2Br_2N_4O_2$ (1677.24): C, 57.29; H, 4.45; N, 3.34. Found: C, 57.27; H, 4.39; N, 3.48.

$Au_2Br_2$4f. <u>*New compound.*</u> The product was obtained in 94% yield (0.13 g, 0.094 mmol) as yellow solid. ^{1}H NMR (400 MHz, $CDCl_3$) δ 7.27 – 7.25 (m, 6H), 7.23 – 7.18 (m, 10H), 6.99 (s, 1H), 6.90 – 6.89 (m, 2H), 6.87 – 6.85 (m, 3H), 6.71 (d, J = 5.3 Hz, 2H), 5.44 (d, J = 9.5 Hz, 2H), 4.31 (s, 4H), 2.28 (d, J = 7.8 Hz, 6H), 2.14 (s, 6H), 2.05 – 2.04 (m, 4H), 1.87 (s, 3H), 1.78 (s, 3H), 1.10 (s, 3H), 1.04 (s, 3H). ^{13}C NMR (101 MHz, $CDCl_3$) δ 172.52, 142.59, 142.09, 141.22, 141.04, 139.65, 135.58, 133.22, 130.22, 129.85, 129.68, 129.49, 129.42, 128.61, 128.53, 127.43, 126.99, 126.60, 124.34, 51.48, 48.28, 28.21, 21.66, 18.12, 17.86, 9.58, 8.31,

8.20. Anal. Calc for $C_{56}H_{58}Au_2Br_2N_4$ (1340.85): C, 50.16; H, 4.36; N, 4.18. Found: C, 49.94; H, 4.44; N, 4.21.

AuBr4g. New compound. The product was obtained in 60% yield (0.051 g, 0.06 mmol) as white solid. ^{1}H NMR (400 MHz, $CDCl_3$) δ 7.26 (t, J = 7.2 Hz, 4H), 7.19 – 7.17 (m, 8H), 7.12 (d, J = 7.3 Hz, 4H), 6.90 (dd, J = 7.7, 1.5 Hz, 4H), 6.77 (s, 2H), 5.25 (s, 2H), 4.12 (t, J = 7.6, 2H), 2.23 (s, 3H), 2.02 (s, 3H), 1.75 (dd, J = 15.3, 7.7 Hz, 2H), 1.41 (dd, J = 15.2, 7.5 Hz, 2H), 1.00 (t, J = 7.3 Hz, 3H), 0.64 (s, 3H). ^{13}C NMR (101 MHz, $CDCl_3$) δ 173.47, 142.40, 142.31, 141.41, 139.58, 133.33, 130.30, 129.86, 129.60, 128.72, 128.44, 127.55, 126.84, 126.76, 123.68, 51.57, 48.56, 33.53, 22.01, 20.08, 14.01, 9.15, 7.88. Anal. Calcd for $C_{42}H_{42}AuBrN_2$ (851.68): C, 59.23; H, 4.97; N, 3.29. Found: C, 59.28; H, 5.12; N, 3.20.

Carboxylative Cyclization of a Propargylamine (PPA)

Previously reported procedure was followed.[6] Prepared according to the procedure using catalyst **$Au_2Br_2$4a - $Au_2Br_2$4g** (1.0 mol%). Reaction was carried out with PPA (0.5 mmol) in MeOH (0.4 mL) under CO_2 atmosphere, at 40 °C for 24 h. After the desired time, the conversion was determined by ^{1}H NMR using 1,3,5-trimethoxybenzene as internal standard. All catalytic reactions were repeated three times, with the results differing by no more than 5%.

3-Benzyl-5-vinyloxazolidin-2-one (6). The product was obtained in 84% yield (85.36 mg, 0.42 mmol) as a yellow liquid. Purification by flash chromatography (Hex/AcOEt = 9/1). ^{1}H NMR (400 MHz, $CDCl_3$) δ 7.38 – 7.26 (m, 5H), 4.56 (qt, J = 6.9, 2.2 Hz, 1H), 4.46 (s, 2H), 3.96 (t, J = 2.2 Hz, 2H), 1.67 (d, J = 6.9 Hz, 3H). ^{13}C NMR (101 MHz, $CDCl_3$) δ 156.20, 141.79, 135.32, 129.07, 128.30, 97.75, 48.00, 47.22, 10.12.

Alkyne Hydration Reaction

Previously reported procedure was followed.[7] According to the procedure, catalyst **$Au_2Br_2$4a - $Au_2Br_2$4g** (0.1 mol%) in THF (10 mg/mL solution in THF), 1,4-dioxane (1.33 mL), $AgSbF_6$ (tip of spatula) were placed in a reaction vial equipped with magnetic stirring bar and stirred for 1 min. After that time, diphenylacethylene (2.0 mmol) and destilled water (0.66 mL) were added to the reaction mixture, then was heated at 120 °C for 18 h. After the desired time, the conversion was determined by ^{1}H NMR using 1,3,5-trimethoxybenzene as internal standard. All catalytic reactions were repeated three times, with the results differing by no more than 5%.

1,2-Diphenylethan-1-one (8). The product was obtained in 78% yield (306.14 mg, 1.56 mmol) as a yellow liquid. Purification by flash chromatography (Petroleum ether/Hex = 9/1). ^{1}H NMR

(400 MHz, $CDCl_3$) δ 8.00 (dd, J = 8.4, 1.2 Hz, 2H), 7.55 – 7.51 (m, 1H), 7.47 – 7.41 (m, 2H), 7.33 – 7.29 (m, 2H), 7.26 – 7.21 (m, 3H), 4.26 (s, 2H). ^{13}C NMR (101 MHz, $CDCl_3$) δ 197.72, 136.66, 134.64, 133.28, 129.59, 128.77, 128.75, 128.71, 126.99, 45.58.

References

1 (a) G. Berthon-Gelloz, M. A. Siegler, A. L. Spek, B. Tinant, J. N. H. Reek and I. E. Markó, *Dalton Trans.*, 2010, **39**, 1444–1446; (b) S. Meiries, G. Le Duc, A. Chartoire, A. Collado, K. Speck, K. S. A. Arachchige, A. M. Z. Slawin and S. P. Nolan, *Chem. - Eur. J.*, 2013, **19**, 17358–17368; (c) F. Izquierdo, S. Manzini and S. P. Nolan, *Chem. Commun.*, 2014, **50**, 14926–14937; (d) W. Chu, T. Zhou, E. Bisz, B. Dziuk, R. Lalancette, R. Szostak and M. Szostak, *Chem. Comm.*, 2022, **58**, 13467–13470; (e) H. Dau, A. Keyes, H. E. Basbug Alhan, E. Ordonez, E. Tsogtgerel, A. P. Gies, E. Auyeung, Z. Zhou, A. Maity, A. Das, D. C. Powers, D. B. Beezer and E. Harth, *J. Am. Chem. Soc.*, 2020, **142**, 21469–21483; (f) P. Shaw, A. R. Kennedy and D. J. Nelson, *Dalton Trans.*, 2016, **45**, 11772–11780; (g) L. Kong, J. Morvan, D. Pichon, M. Jean, M. Albalat, T. Vives, S. Colombel-Rouen, M. Giorgi, V. Dorcet, T. Roisnel, C. Crévisy, D. Nuel, P. Nava, S. Humbel, N. Vanthuyne, M. Mauduit and H. Clavier, *J. Am. Chem. Soc.*, 2020, **142**, 93–98.

2 (a) M. Kuriyama, N. Hamaguchi, G. Yano, K. Tsukuda, K. Sato and O. Onomura, *J. Org. Chem.*, 2016, **81**, 8934–8946; (b) P. Podchorodecka, B. Dziuk, R. Szostak, M. Szostak and E. Bisz, *Dalton Trans.*, 2023, **52**, 13608–13617; (c) Patent 20240181438, 2024.

3 (a) T. Scherg, S. Schneider, G. Frey, J. Schwarz, E. Herdtweck and W. Herrmann, *Synlett*, 2006, 2894–290; (b) M. Micksch and T. Strassner, Eur. J. Inorg. Chem., 2012, 5872–5880.

4 (a) S. Ahrens, A. Peritz and T. Strassner, *Angew. Chem. Int. Ed.*, 2009, **48**, 7908–7910; (b) F. He, X. Chen, Y. Shen, Y. Li, A. Liu, S. Liu, T. Mori and Y. Zhang, *J. Mater. Chem. A*, 2016, **4**, 6630–6638.

5 M. Baron, E. Battistel, C. Tubaro, A. Biffis, L. Armelao, M. Rancan and C. Graiff, *Organometallics*, 2018, **37**, 4213–4223.

6 (a) Y. Kayaki, M. Yamamoto, T. Suzuki and T. Ikariya, *Green Chem.*, 2006, **8**, 1019; (b) S. Hase, Y. Kayaki and T. Ikariya, *Organometallics*, 2013, **32**, 5285–5288; (c) T. A. C. A. Bayrakdar, F. Nahra, J. V. Davis, M. M. Gamage, B. Captain, M. Temprado, M. Marazzi, M. Saab, K. Van Hecke, D. Ormerod, C. D. Hoff and S. P. Nolan, *Organometallics*, 2020, **39**, 2907–2916.

7 (a) N. Marion, R. S. Ramón and S. P. Nolan, *J. Am. Chem. Soc.*, 2009, **131**, 448–449; (b) G. A. Fernández, A. B. Chopa and G. F. Silbestri, *Catal. Sci. Technol.*, 2016, **6**, 1921–1929; (c) F. Nahra, N. V. Tzouras, A. Collado and S. P. Nolan, *Nat. Protoc.*, 2021, **16**, 1476–1493.

Crystallographic Studies

Experimental

The single crystals of the complex 1-7 were collected on a Rigaku Oxford Diffraction XtaLAB SynergyR DW diffractometer equipped with a HyPix ARC 150° Hybrid Photon Counting (HPC) detector using CuKα (λ = 1.54184 Å) at 100 K. The corrections to the Lorentz and polarization factors were applied to the reflection intensities.[1] Data was processed using the CrysAlisPro software. The structures were solved by direct methods using SHELXS and refined by full-matrix least-squares methods based F^2 using SHELXL.[2,3] The hydrogen atoms were determined from the geometric concepts and refined in a riding model with isotropic temperature factors of 1.2 times the Ueq value of the parent atom. All non-hydrogen atoms were located from difference Fourier synthesis and refined by least squares method in the full-matrix anisotropic approximation. Disordered dichloromethane as solvent was omitted from the refinement of 2-6 using a solvent mask (Squeeze) in those structures. The resulting cif have been properly reformatted with the information about the void space and electron density. The crystallographic data for compounds and details of X-ray experiment are collected in the Supplementary information Tables. The structure drawings in ESI were prepared by using Mercury program.[4] The coordinates of atoms and other parameters for structures were deposited with the Cambridge Crystallographic Data Centre: 2410857-2410863 for 1-7; 12 Union Road, Cambridge CB2 1EZ, UK (Fax,_44-(1223)336-033, E-mail deposit@ccdc.cam.ac.uk).

Fig. S1. The molecular structure of complexes **Au_2Br_24a-Au_2Br_24g**. Hydrogen atoms and solvent molecules have been omitted for clarity.

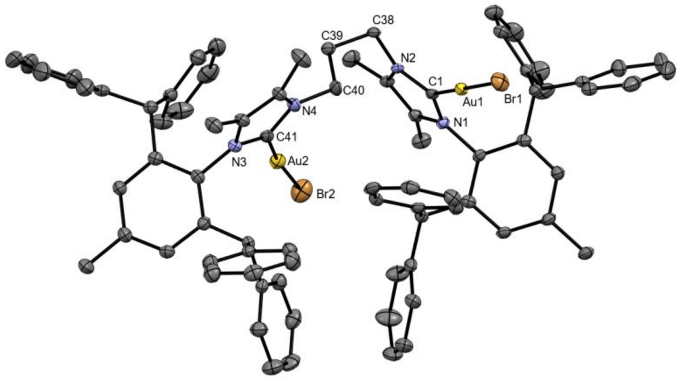

Au_2Br_24a

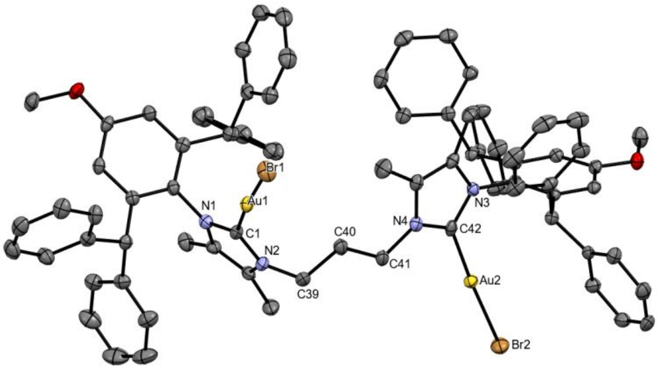

Au_2Br_24b

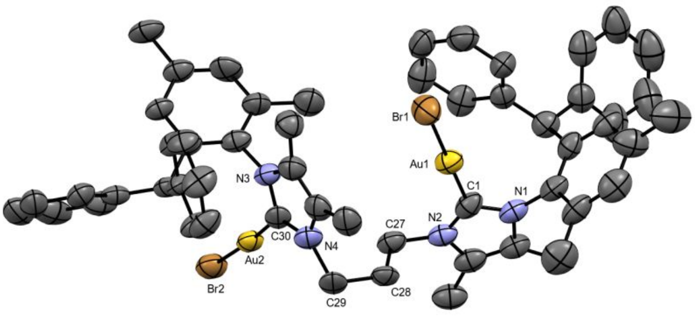

Au_2Br_24c

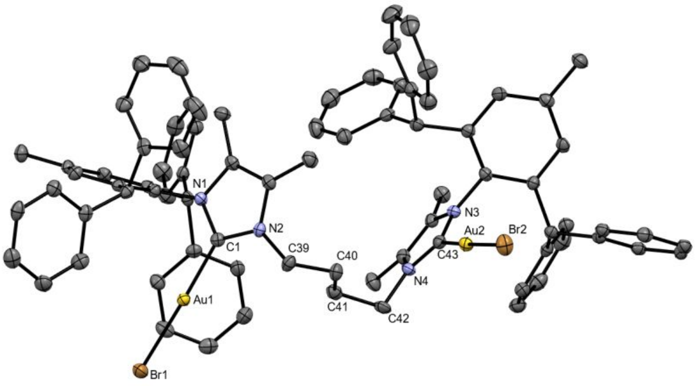

$Au_2Br_2$4d

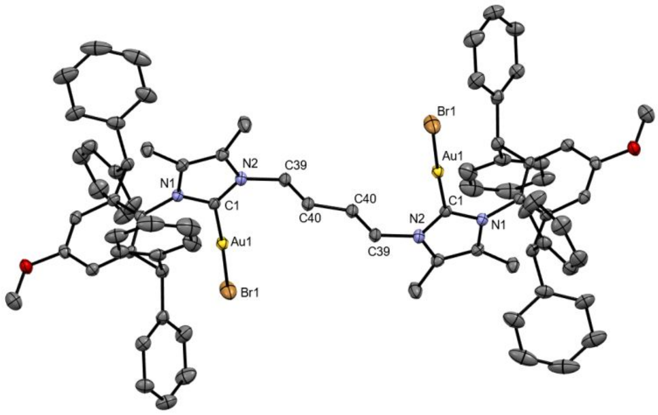

$Au_2Br_2$4e

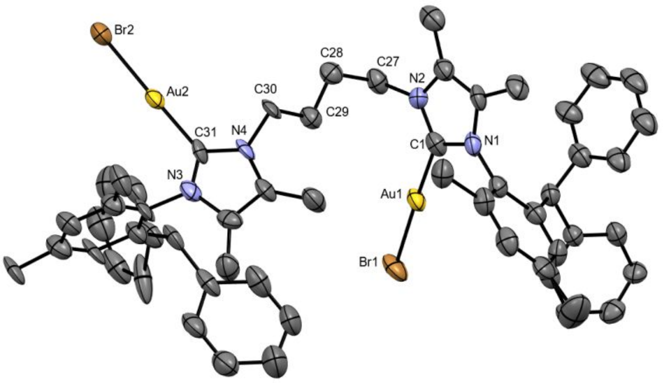

$Au_2Br_2$4f

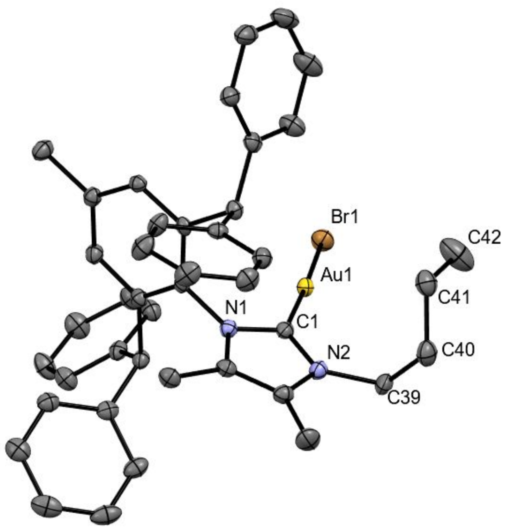

AuBr4g

References

1 CrysAlis CCD; Oxford Diffraction Ltd: Abingdon, England, 2002. CrysAlis RED; Oxford Diffraction Ltd: Abingdon, England, 2002.

2 G. M. Sheldrick, A short history of SHELX, Acta Crystallogr. Sect. A 64 (2008) 112−122, https://doi.org/10.1107/S0108767307043930.

3 G. M. Sheldrick, Crystal structure refinement with SHELXL, Acta Crystallogr. Sect. C 71 (2015) 3–8, https://doi.org/10.1107/S2053229614024218.

4 C. F. Macrae, I. J. Bruno, J. A. Chisholm, P. R. Edgington, P. McCabe, E. Pidcock, L. Rodriguez-Monge, R. Taylor, J. van de Streek, P. A. Wood, New Features for the Visualization and Investigation of Crystal Structures, Journal of Applied Crystallography 41 (2008) 466–470, http://doi.org/10.1107/S0021889807067908.

5 O. V. Dolomanov, L. J.Bourhis, R. J, Gildea, J. A. K. Howard & H. Puschmann, J. Appl. Cryst. 42 (2009) 339−341, https://doi.org/10.1107/S0021889808042726.

Crystallographic data

Table S1. Experimental details

Experiments were carried out at 100 K with Cu $K\alpha$ radiation using a XtaLAB Synergy R, DW system, HyPix-Arc 150. H-atom parameters were constrained.

	**1**	**2**	**3**	**4**
	CCDC:2410857	CCDC:2410858	CCDC:2410859	CCDC:2410860
**Crystal data**				
Chemical formula	$C_{82}H_{78}Au_2Br_2Cl_6N_4$	$C_{56}H_{57}Au_2Br_2N_4$	$C_{55}H_{56}Au_2Br_2N_4$	$C_{79}H_{72}Au_2Br_2N_4O_2$
M_r	1885.93	1339.81	1326.79	1663.15
Crystal system, space group	Monoclinic, $P2_1/n$	Monoclinic, $P2_1/c$	Triclinic, $P\bar{1}$	Triclinic, $P\bar{1}$
a, b, c (Å)	16.3581 (1), 19.5486 (1), 23.9004 (1)	14.76288 (12), 27.9564 (3), 16.02061 (16)	12.6393 (3), 13.7114 (5), 18.8862 (8)	15.6478 (1), 15.9214 (1), 15.9670 (1)
α, β, γ (°)	90, 104.046 (1), 90	90, 103.4418 (9), 90	70.118 (3), 80.554 (3), 63.646 (3)	76.522 (1), 64.646 (1), 89.022 (1)
V (Å^3)	7414.31 (7)	6430.85 (11)	2757.71 (19)	3480.25 (5)
Z	4	4	2	2
μ (mm^{-1})	10.95	10.15	11.83	9.53
Crystal size (mm)	0.40 × 0.35 × 0.30	0.4 × 0.35 × 0.3	0.5 × 0.45 × 0.4	0.45 × 0.43 × 0.40
**Data collection**				
Absorption correction	Multi-scan SCALE3 ABSPACK (Rigaku Oxford Diffraction).	Multi-scan SCALE3 ABSPACK (Rigaku Oxford Diffraction).	Multi-scan SCALE3 ABSPACK (Rigaku Oxford Diffraction).	Multi-scan SCALE3 ABSPACK (Rigaku Oxford Diffraction).
T_{min}, T_{max}	0.031, 1.000	0.043, 1.000	0.007, 1.000	0.043, 1.000
No. of measured, independent and observed [$I > 2\sigma(I)$] reflections	87220, 15179, 14733	101146, 12524, 11120	56096, 11200, 7868	87266, 14216, 13694
R_{int}	0.031	0.056	0.076	0.028
$(\sin \theta/\lambda)_{max}$ (Å^{-1})	0.628	0.631	0.628	0.629
**Refinement**				
$R[F^2 > 2\sigma(F^2)]$, $wR(F^2)$, S	0.040, 0.106, 1.06	0.077, 0.220, 1.04	0.057, 0.160, 1.09	0.032, 0.088, 1.08
No. of reflections	15179	12524	11200	14216
No. of parameters	871	585	576	808
No. of restraints	0	169	0	0

	$w = 1/[$ ʃ$^2(F_o^2) + (0.0516P)^2 + 47.2703P]$ where $P = (F_o^2 + 2F_c^2)/3$	$w = 1/[$ ʃ$^2(F_o^2) + (0.1164P)^2 + 105.9249P]$ where $P = (F_o^2 + 2F_c^2)/3$	$w = 1/[$ ʃ$^2(F_o^2) + (0.081P)^2 + 2.2764P]$ where $P = (F_o^2 + 2F_c^2)/3$	$w = 1/[$ ʃ$^2(F_o^2) + (0.0543P)^2 + 6.3887P]$ where $P = (F_o^2 + 2F_c^2)/3$
⊗〉$_{max}$, ⊗〉$_{min}$ (e Å^{-3})	1.82, -3.11	5.08, -3.36	2.12, -2.34	1.16, -2.59

	**5**<br>CCDC:2410861	**6**<br>CCDC:2410862	**7**<br>CCDC:2410863
**Crystal data**			
Chemical formula	$C_{80}H_{74}Au_2Br_2N_4$	$C_{80}H_{74}Au_2Br_2N_4O_2$	$C_{42}H_{42}AuBrN_2$
M_r	1645.18	1677.18	851.65
Crystal system, space group	Monoclinic, $P2_1/c$	Monoclinic, $P2_1/c$	Monoclinic, $P2_1/n$
a, b, c (Å)	18.9330 (1), 24.4434 (1), 17.7039 (1)	9.1212 (1), 14.7272 (1), 30.3187 (2)	14.25042 (7), 13.48114 (6), 18.64883 (9)
〈, ®, © (°)	90, 106.219 (1), 90	90, 92.161 (1), 90	90, 94.5675 (5), 90
V (Å^3)	7867.05 (8)	4069.81 (6)	3571.28 (3)
Z	4	2	4
μ (mm^{-1})	8.41	8.15	9.28
Crystal size (mm)	0.28 × 0.24 × 0.20	0.42 × 0.39 × 0.36	0.38 × 0.30 × 0.28
**Data collection**			
Absorption correction	Multi-scan *CrysAlis PRO* 1.171.43.105a (Rigaku Oxford Diffraction)	Multi-scan *CrysAlis PRO* 1.171.43.105a (Rigaku Oxford Diffraction)	Multi-scan *CrysAlis PRO* 1.171.43.105a (Rigaku Oxford Diffraction)
T_{min}, T_{max}	0.180, 1.000	0.117, 1.000	0.659, 1.000
No. of measured, independent and observed [$I > 2$ ʃ(I)] reflections	113460, 16082, 15120	48299, 8323, 8138	48459, 7325, 7229
R_{int}	0.034	0.032	0.020
(sin ⋃/⌊)$_{max}$ (Å^{-1})	0.628	0.628	0.628
**Refinement**			
$R[F^2 > 2$ ʃ$(F^2)]$, $wR(F^2)$, S	0.030, 0.074, 1.05	0.027, 0.071, 1.05	0.020, 0.052, 1.10
No. of reflections	16082	8323	7325
No. of parameters	799	409	419
No. of restraints	0	0	0
	$w = 1/[$ ʃ$^2(F_o^2) + (0.0325P)^2 + 25.0984P]$ where $P = (F_o^2 +$	$w = 1/[$ ʃ$^2(F_o^2) + (0.0357P)^2 + 8.6674P]$ where $P = (F_o^2 + 2F_c^2)/3$	$w = 1/[$ ʃ$^2(F_o^2) + (0.0241P)^2 + 5.1496P]$ where $P = (F_o^2 + 2F_c^2)/3$

	$2F_c^2$)/3		
$\otimes\rangle_{max}$, $\otimes\rangle_{min}$ (e Å^{-3})	1.73, -1.70	0.75, -1.57	0.75, -0.98

Computer programs: *CrysAlis PRO* 1.171.42.42a (Rigaku OD, 2022), *CrysAlis PRO* 1.171.43.105a (Rigaku OD, 2024), *SHELXL2014*/7 (Sheldrick, 2014), *SHELXT* (Sheldrick, 2015), *SHELXL* 2014/7 (Sheldrick, 2015), *SHELXTL* (Sheldrick, 2008), Olex2 1.3 (Dolomanov *et al.*, 2009).

Table S2. Selected geometric parameters (Å, º).

**$Au_2Br_2$4a**			
Au1—C1	1.985 (4)	C37—H37A	0.9800
Au1—Br1	2.4039 (5)	C37—H37B	0.9800
Au2—C41	1.976 (5)	C37—H37C	0.9800
Au2—Br2	2.3963 (6)	C38—C39	1.534 (6)
Cl1—C79	1.791 (10)	C38—H38A	0.9900
Cl2—C79	1.659 (11)	C38—H38B	0.9900
Cl3—C80	1.766 (7)	C39—C40	1.514 (6)
Cl4—C80	1.766 (8)	C39—H39A	0.9900
Cl5—C81	1.71 (2)	C39—H39B	0.9900
Cl6—C81	1.77 (2)	C40—H40A	0.9900
N1—C1	1.354 (6)	C40—H40B	0.9900
N1—C2	1.408 (6)	C42—C43	1.365 (6)
N1—C4	1.441 (5)	C42—C77	1.482 (7)
N2—C1	1.367 (5)	C43—C78	1.487 (7)
N2—C3	1.395 (6)	C44—C45	1.401 (6)
N2—C38	1.470 (5)	C44—C49	1.410 (6)
N3—C41	1.369 (6)	C45—C46	1.397 (6)
N3—C42	1.400 (6)	C45—C50	1.520 (6)
N3—C44	1.439 (5)	C46—C47	1.392 (7)
N4—C41	1.359 (6)	C46—H46	0.9500
N4—C43	1.385 (6)	C47—C48	1.392 (7)
N4—C40	1.464 (5)	C47—C63	1.498 (6)
C2—C3	1.350 (6)	C48—C49	1.385 (6)
C2—C36	1.480 (6)	C48—H48	0.9500
C3—C37	1.492 (6)	C49—C64	1.537 (6)
C4—C5	1.400 (6)	C50—C51	1.526 (6)
C4—C9	1.407 (6)	C50—C57	1.531 (6)
C5—C6	1.395 (6)	C50—H50	1.0000
C5—C10	1.530 (6)	C51—C56	1.386 (7)
C6—C7	1.397 (6)	C51—C52	1.394 (7)
C6—H6	0.9500	C52—C53	1.384 (7)
C7—C8	1.389 (6)	C52—H52	0.9500

C7—C22	1.506 (6)	C53—C54	1.390 (7)
C8—C9	1.388 (6)	C53—H53	0.9500
C8—H8	0.9500	C54—C55	1.384 (7)
C9—C23	1.526 (6)	C54—H54	0.9500
C10—C16	1.534 (6)	C55—C56	1.382 (7)
C10—C11	1.537 (6)	C55—H55	0.9500
C10—H10	1.0000	C56—H56	0.9500
C11—C15	1.389 (7)	C57—C62	1.394 (7)
C11—C12	1.392 (6)	C57—C58	1.396 (7)
C12—C82	1.399 (7)	C58—C59	1.380 (7)
C12—H12	0.9500	C58—H58	0.9500
C13—C14	1.384 (7)	C59—C60	1.397 (8)
C13—C82	1.384 (8)	C59—H59	0.9500
C13—H13	0.9500	C60—C61	1.377 (8)
C14—C15	1.385 (7)	C60—H60	0.9500
C14—H14	0.9500	C61—C62	1.390 (7)
C15—H15	0.9500	C61—H61	0.9500
C16—C17	1.379 (7)	C62—H62	0.9500
C16—C21	1.388 (7)	C63—H63A	0.9800
C17—C18	1.397 (7)	C63—H63B	0.9800
C17—H17	0.9500	C63—H63C	0.9800
C18—C19	1.374 (8)	C64—C71	1.526 (7)
C18—H18	0.9500	C64—C65	1.535 (7)
C19—C20	1.392 (9)	C64—H64	1.0000
C19—H19	0.9500	C65—C70	1.380 (8)
C20—C21	1.388 (8)	C65—C66	1.390 (9)
C20—H20	0.9500	C66—C67	1.384 (8)
C21—H21	0.9500	C66—H66	0.9500
C22—H22A	0.9800	C67—C68	1.376 (12)
C22—H22B	0.9800	C67—H67	0.9500
C22—H22C	0.9800	C68—C69	1.372 (12)
C23—C24	1.515 (6)	C68—H68	0.9500
C23—C30	1.525 (6)	C69—C70	1.401 (9)
C23—H23	1.0000	C69—H69	0.9500
C24—C29	1.389 (6)	C70—H70	0.9500
C24—C25	1.400 (7)	C71—C72	1.386 (7)
C25—C26	1.390 (8)	C71—C76	1.391 (7)
C25—H25	0.9500	C72—C73	1.386 (8)
C26—C27	1.382 (8)	C72—H72	0.9500
C26—H26	0.9500	C73—C74	1.384 (9)
C27—C28	1.383 (8)	C73—H73	0.9500

C27—H27	0.9500	C74—C75	1.371 (9)
C28—C29	1.391 (7)	C74—H74	0.9500
C28—H28	0.9500	C75—C76	1.383 (8)
C29—H29	0.9500	C75—H75	0.9500
C30—C31	1.387 (7)	C76—H76	0.9500
C30—C35	1.394 (7)	C77—H77A	0.9800
C31—C32	1.396 (8)	C77—H77B	0.9800
C31—H31	0.9500	C77—H77C	0.9800
C32—C33	1.363 (10)	C78—H78A	0.9800
C32—H32	0.9500	C78—H78B	0.9800
C33—C34	1.382 (9)	C78—H78C	0.9800
C33—H33	0.9500	C79—H79A	0.9900
C34—C35	1.382 (7)	C79—H79B	0.9900
C34—H34	0.9500	C80—H80A	0.9900
C35—H35	0.9500	C80—H80B	0.9900
C36—H36A	0.9800	C81—H81A	0.9900
C36—H36B	0.9800	C81—H81B	0.9900
C36—H36C	0.9800	C82—H82	0.9500

C1—Au1—Br1	177.48 (12)	C39—C40—H40A	108.9
C41—Au2—Br2	172.10 (13)	N4—C40—H40B	108.9
C1—N1—C2	111.3 (4)	C39—C40—H40B	108.9
C1—N1—C4	126.1 (4)	H40A—C40—H40B	107.7
C2—N1—C4	122.6 (4)	N4—C41—N3	104.1 (4)
C1—N2—C3	110.8 (4)	N4—C41—Au2	123.8 (3)
C1—N2—C38	124.6 (4)	N3—C41—Au2	132.1 (3)
C3—N2—C38	124.6 (4)	C43—C42—N3	106.2 (4)
C41—N3—C42	111.2 (4)	C43—C42—C77	131.1 (5)
C41—N3—C44	124.1 (4)	N3—C42—C77	122.7 (4)
C42—N3—C44	124.7 (4)	C42—C43—N4	106.6 (4)
C41—N4—C43	112.0 (4)	C42—C43—C78	130.5 (5)
C41—N4—C40	123.1 (4)	N4—C43—C78	122.8 (4)
C43—N4—C40	124.6 (4)	C45—C44—C49	121.6 (4)
N1—C1—N2	104.6 (4)	C45—C44—N3	120.2 (4)
N1—C1—Au1	127.3 (3)	C49—C44—N3	118.1 (4)
N2—C1—Au1	128.1 (3)	C46—C45—C44	117.5 (4)
C3—C2—N1	106.1 (4)	C46—C45—C50	120.2 (4)
C3—C2—C36	130.9 (4)	C44—C45—C50	122.1 (4)
N1—C2—C36	122.9 (4)	C47—C46—C45	122.2 (4)
C2—C3—N2	107.2 (4)	C47—C46—H46	118.9
C2—C3—C37	130.1 (4)	C45—C46—H46	118.9

N2—C3—C37	122.7 (4)	C46—C47—C48	118.6 (4)
C5—C4—C9	121.9 (4)	C46—C47—C63	120.5 (4)
C5—C4—N1	119.1 (4)	C48—C47—C63	120.9 (4)
C9—C4—N1	118.9 (4)	C49—C48—C47	121.7 (4)
C6—C5—C4	117.3 (4)	C49—C48—H48	119.1
C6—C5—C10	121.7 (4)	C47—C48—H48	119.1
C4—C5—C10	120.6 (4)	C48—C49—C44	118.3 (4)
C5—C6—C7	122.4 (4)	C48—C49—C64	121.4 (4)
C5—C6—H6	118.8	C44—C49—C64	120.1 (4)
C7—C6—H6	118.8	C45—C50—C51	113.0 (4)
C8—C7—C6	118.1 (4)	C45—C50—C57	112.1 (4)
C8—C7—C22	120.3 (4)	C51—C50—C57	111.1 (4)
C6—C7—C22	121.6 (4)	C45—C50—H50	106.7
C9—C8—C7	122.1 (4)	C51—C50—H50	106.7
C9—C8—H8	118.9	C57—C50—H50	106.7
C7—C8—H8	118.9	C56—C51—C52	117.6 (5)
C8—C9—C4	118.0 (4)	C56—C51—C50	123.4 (4)
C8—C9—C23	120.8 (4)	C52—C51—C50	118.7 (4)
C4—C9—C23	121.3 (4)	C53—C52—C51	121.2 (5)
C5—C10—C16	112.0 (4)	C53—C52—H52	119.4
C5—C10—C11	108.3 (4)	C51—C52—H52	119.4
C16—C10—C11	114.0 (4)	C52—C53—C54	120.2 (5)
C5—C10—H10	107.4	C52—C53—H53	119.9
C16—C10—H10	107.4	C54—C53—H53	119.9
C11—C10—H10	107.4	C55—C54—C53	119.1 (5)
C15—C11—C12	117.9 (5)	C55—C54—H54	120.4
C15—C11—C10	122.8 (4)	C53—C54—H54	120.4
C12—C11—C10	119.3 (4)	C56—C55—C54	120.2 (5)
C11—C12—C82	120.5 (5)	C56—C55—H55	119.9
C11—C12—H12	119.7	C54—C55—H55	119.9
C82—C12—H12	119.7	C55—C56—C51	121.7 (5)
C14—C13—C82	119.2 (5)	C55—C56—H56	119.1
C14—C13—H13	120.4	C51—C56—H56	119.1
C82—C13—H13	120.4	C62—C57—C58	118.3 (4)
C13—C14—C15	120.0 (5)	C62—C57—C50	122.9 (4)
C13—C14—H14	120.0	C58—C57—C50	118.8 (4)
C15—C14—H14	120.0	C59—C58—C57	121.1 (5)
C14—C15—C11	121.8 (5)	C59—C58—H58	119.4
C14—C15—H15	119.1	C57—C58—H58	119.4
C11—C15—H15	119.1	C58—C59—C60	119.8 (5)
C17—C16—C21	118.6 (5)	C58—C59—H59	120.1

C17—C16—C10	122.5 (4)	C60—C59—H59	120.1
C21—C16—C10	118.9 (4)	C61—C60—C59	119.8 (5)
C16—C17—C18	120.9 (5)	C61—C60—H60	120.1
C16—C17—H17	119.6	C59—C60—H60	120.1
C18—C17—H17	119.6	C60—C61—C62	120.2 (5)
C19—C18—C17	120.4 (5)	C60—C61—H61	119.9
C19—C18—H18	119.8	C62—C61—H61	119.9
C17—C18—H18	119.8	C61—C62—C57	120.8 (5)
C18—C19—C20	118.9 (5)	C61—C62—H62	119.6
C18—C19—H19	120.5	C57—C62—H62	119.6
C20—C19—H19	120.5	C47—C63—H63A	109.5
C21—C20—C19	120.5 (6)	C47—C63—H63B	109.5
C21—C20—H20	119.7	H63A—C63—H63B	109.5
C19—C20—H20	119.7	C47—C63—H63C	109.5
C16—C21—C20	120.6 (5)	H63A—C63—H63C	109.5
C16—C21—H21	119.7	H63B—C63—H63C	109.5
C20—C21—H21	119.7	C71—C64—C65	113.5 (4)
C7—C22—H22A	109.5	C71—C64—C49	110.2 (4)
C7—C22—H22B	109.5	C65—C64—C49	112.4 (4)
H22A—C22—H22B	109.5	C71—C64—H64	106.8
C7—C22—H22C	109.5	C65—C64—H64	106.8
H22A—C22—H22C	109.5	C49—C64—H64	106.8
H22B—C22—H22C	109.5	C70—C65—C66	118.4 (5)
C24—C23—C30	115.5 (4)	C70—C65—C64	123.2 (5)
C24—C23—C9	111.6 (4)	C66—C65—C64	118.5 (5)
C30—C23—C9	109.9 (4)	C67—C66—C65	121.4 (7)
C24—C23—H23	106.4	C67—C66—H66	119.3
C30—C23—H23	106.4	C65—C66—H66	119.3
C9—C23—H23	106.4	C68—C67—C66	119.7 (7)
C29—C24—C25	118.2 (4)	C68—C67—H67	120.1
C29—C24—C23	120.2 (4)	C66—C67—H67	120.1
C25—C24—C23	121.6 (4)	C69—C68—C67	119.9 (6)
C26—C25—C24	120.4 (5)	C69—C68—H68	120.1
C26—C25—H25	119.8	C67—C68—H68	120.1
C24—C25—H25	119.8	C68—C69—C70	120.4 (7)
C27—C26—C25	120.5 (5)	C68—C69—H69	119.8
C27—C26—H26	119.8	C70—C69—H69	119.8
C25—C26—H26	119.8	C65—C70—C69	120.2 (7)
C26—C27—C28	119.7 (5)	C65—C70—H70	119.9
C26—C27—H27	120.1	C69—C70—H70	119.9
C28—C27—H27	120.1	C72—C71—C76	119.1 (5)

C27—C28—C29	119.8 (5)	C72—C71—C64	120.9 (4)
C27—C28—H28	120.1	C76—C71—C64	120.0 (4)
C29—C28—H28	120.1	C71—C72—C73	120.2 (5)
C24—C29—C28	121.3 (5)	C71—C72—H72	119.9
C24—C29—H29	119.4	C73—C72—H72	119.9
C28—C29—H29	119.4	C74—C73—C72	120.5 (6)
C31—C30—C35	118.5 (5)	C74—C73—H73	119.8
C31—C30—C23	123.0 (5)	C72—C73—H73	119.8
C35—C30—C23	118.5 (4)	C75—C74—C73	119.2 (5)
C30—C31—C32	120.0 (6)	C75—C74—H74	120.4
C30—C31—H31	120.0	C73—C74—H74	120.4
C32—C31—H31	120.0	C74—C75—C76	121.0 (5)
C33—C32—C31	121.0 (6)	C74—C75—H75	119.5
C33—C32—H32	119.5	C76—C75—H75	119.5
C31—C32—H32	119.5	C75—C76—C71	120.0 (5)
C32—C33—C34	119.5 (5)	C75—C76—H76	120.0
C32—C33—H33	120.2	C71—C76—H76	120.0
C34—C33—H33	120.2	C42—C77—H77A	109.5
C33—C34—C35	120.3 (6)	C42—C77—H77B	109.5
C33—C34—H34	119.8	H77A—C77—H77B	109.5
C35—C34—H34	119.8	C42—C77—H77C	109.5
C34—C35—C30	120.7 (5)	H77A—C77—H77C	109.5
C34—C35—H35	119.6	H77B—C77—H77C	109.5
C30—C35—H35	119.6	C43—C78—H78A	109.5
C2—C36—H36A	109.5	C43—C78—H78B	109.5
C2—C36—H36B	109.5	H78A—C78—H78B	109.5
H36A—C36—H36B	109.5	C43—C78—H78C	109.5
C2—C36—H36C	109.5	H78A—C78—H78C	109.5
H36A—C36—H36C	109.5	H78B—C78—H78C	109.5
H36B—C36—H36C	109.5	Cl2—C79—Cl1	112.9 (6)
C3—C37—H37A	109.5	Cl2—C79—H79A	109.0
C3—C37—H37B	109.5	Cl1—C79—H79A	109.0
H37A—C37—H37B	109.5	Cl2—C79—H79B	109.0
C3—C37—H37C	109.5	Cl1—C79—H79B	109.0
H37A—C37—H37C	109.5	H79A—C79—H79B	107.8
H37B—C37—H37C	109.5	Cl4—C80—Cl3	112.0 (4)
N2—C38—C39	112.1 (3)	Cl4—C80—H80A	109.2
N2—C38—H38A	109.2	Cl3—C80—H80A	109.2
C39—C38—H38A	109.2	Cl4—C80—H80B	109.2
N2—C38—H38B	109.2	Cl3—C80—H80B	109.2
C39—C38—H38B	109.2	H80A—C80—H80B	107.9

H38A—C38—H38B	107.9	Cl5—C81—Cl6	114.5 (7)
C40—C39—C38	110.2 (4)	Cl5—C81—H81A	108.6
C40—C39—H39A	109.6	Cl6—C81—H81A	108.6
C38—C39—H39A	109.6	Cl5—C81—H81B	108.6
C40—C39—H39B	109.6	Cl6—C81—H81B	108.6
C38—C39—H39B	109.6	H81A—C81—H81B	107.6
H39A—C39—H39B	108.1	C13—C82—C12	120.5 (5)
N4—C40—C39	113.3 (4)	C13—C82—H82	119.7
N4—C40—H40A	108.9	C12—C82—H82	119.7

C2—N1—C1—N2	0.1 (4)	C38—C39—C40—N4	178.6 (4)
C4—N1—C1—N2	176.9 (4)	C43—N4—C41—N3	-0.8 (5)
C2—N1—C1—Au1	-178.0 (3)	C40—N4—C41—N3	173.4 (4)
C4—N1—C1—Au1	-1.3 (6)	C43—N4—C41—Au2	-178.4 (3)
C3—N2—C1—N1	0.2 (4)	C40—N4—C41—Au2	-4.2 (6)
C38—N2—C1—N1	179.0 (4)	C42—N3—C41—N4	0.3 (5)
C3—N2—C1—Au1	178.4 (3)	C44—N3—C41—N4	177.7 (4)
C38—N2—C1—Au1	-2.8 (6)	C42—N3—C41—Au2	177.6 (3)
C1—N1—C2—C3	-0.4 (5)	C44—N3—C41—Au2	-5.0 (7)
C4—N1—C2—C3	-177.3 (4)	C41—N3—C42—C43	0.4 (5)
C1—N1—C2—C36	177.1 (4)	C44—N3—C42—C43	-177.1 (4)
C4—N1—C2—C36	0.2 (6)	C41—N3—C42—C77	-177.1 (4)
N1—C2—C3—N2	0.6 (5)	C44—N3—C42—C77	5.4 (7)
C36—C2—C3—N2	-176.7 (5)	N3—C42—C43—N4	-0.8 (5)
N1—C2—C3—C37	178.7 (5)	C77—C42—C43—N4	176.3 (5)
C36—C2—C3—C37	1.4 (8)	N3—C42—C43—C78	175.9 (5)
C1—N2—C3—C2	-0.5 (5)	C77—C42—C43—C78	-6.9 (9)
C38—N2—C3—C2	-179.3 (4)	C41—N4—C43—C42	1.1 (5)
C1—N2—C3—C37	-178.8 (4)	C40—N4—C43—C42	-173.0 (4)
C38—N2—C3—C37	2.4 (6)	C41—N4—C43—C78	-175.9 (5)
C1—N1—C4—C5	105.9 (5)	C40—N4—C43—C78	9.9 (7)
C2—N1—C4—C5	-77.7 (5)	C41—N3—C44—C45	83.0 (6)
C1—N1—C4—C9	-76.9 (6)	C42—N3—C44—C45	-99.9 (5)
C2—N1—C4—C9	99.4 (5)	C41—N3—C44—C49	-96.5 (5)
C9—C4—C5—C6	-5.0 (7)	C42—N3—C44—C49	80.6 (6)
N1—C4—C5—C6	172.1 (4)	C49—C44—C45—C46	1.5 (7)
C9—C4—C5—C10	168.1 (4)	N3—C44—C45—C46	-178.0 (4)
N1—C4—C5—C10	-14.8 (6)	C49—C44—C45—C50	-175.3 (4)
C4—C5—C6—C7	2.4 (7)	N3—C44—C45—C50	5.2 (7)

C10—C5—C6—C7	-170.6 (4)	C44—C45—C46—C47	-1.3 (7)
C5—C6—C7—C8	1.6 (7)	C50—C45—C46—C47	175.6 (4)
C5—C6—C7—C22	-179.7 (5)	C45—C46—C47—C48	-0.2 (7)
C6—C7—C8—C9	-3.3 (7)	C45—C46—C47—C63	179.6 (5)
C22—C7—C8—C9	178.0 (5)	C46—C47—C48—C49	1.5 (7)
C7—C8—C9—C4	0.8 (7)	C63—C47—C48—C49	-178.3 (5)
C7—C8—C9—C23	-179.7 (4)	C47—C48—C49—C44	-1.3 (7)
C5—C4—C9—C8	3.5 (7)	C47—C48—C49—C64	174.6 (5)
N1—C4—C9—C8	-173.6 (4)	C45—C44—C49—C48	-0.2 (7)
C5—C4—C9—C23	-176.0 (4)	N3—C44—C49—C48	179.2 (4)
N1—C4—C9—C23	7.0 (6)	C45—C44—C49—C64	-176.2 (4)
C6—C5—C10—C16	-29.5 (6)	N3—C44—C49—C64	3.3 (7)
C4—C5—C10—C16	157.7 (4)	C46—C45—C50—C51	-81.7 (5)
C6—C5—C10—C11	97.1 (5)	C44—C45—C50—C51	95.0 (5)
C4—C5—C10—C11	-75.7 (5)	C46—C45—C50—C57	44.7 (6)
C5—C10—C11—C15	-26.0 (6)	C44—C45—C50—C57	-138.6 (4)
C16—C10—C11—C15	99.4 (5)	C45—C50—C51—C56	31.9 (6)
C5—C10—C11—C12	152.0 (4)	C57—C50—C51—C56	-95.0 (5)
C16—C10—C11—C12	-82.5 (5)	C45—C50—C51—C52	-153.3 (4)
C15—C11—C12—C82	2.5 (7)	C57—C50—C51—C52	79.7 (5)
C10—C11—C12—C82	-175.6 (4)	C56—C51—C52—C53	0.0 (7)
C82—C13—C14—C15	1.5 (7)	C50—C51—C52—C53	-175.1 (5)
C13—C14—C15—C11	-1.4 (8)	C51—C52—C53—C54	0.2 (8)
C12—C11—C15—C14	-0.6 (7)	C52—C53—C54—C55	-0.6 (8)
C10—C11—C15—C14	177.5 (4)	C53—C54—C55—C56	0.8 (8)

C5—C10—C16—C17	102.4 (5)	C54—C55—C56—C51	-0.7 (8)
C11—C10—C16—C17	-21.1 (6)	C52—C51—C56—C55	0.3 (7)
C5—C10—C16—C21	-77.9 (6)	C50—C51—C56—C55	175.1 (5)
C11—C10—C16—C21	158.6 (5)	C45—C50—C57—C62	-106.6 (5)
C21—C16—C17—C18	2.2 (7)	C51—C50—C57—C62	20.9 (6)
C10—C16—C17—C18	-178.0 (4)	C45—C50—C57—C58	74.8 (5)
C16—C17—C18—C19	0.0 (8)	C51—C50—C57—C58	-157.7 (4)
C17—C18—C19—C20	-2.2 (9)	C62—C57—C58—C59	-1.1 (7)
C18—C19—C20—C21	2.2 (10)	C50—C57—C58—C59	177.5 (4)
C17—C16—C21—C20	-2.2 (9)	C57—C58—C59—C60	1.0 (8)
C10—C16—C21—C20	178.0 (5)	C58—C59—C60—C61	-0.3 (8)
C19—C20—C21—C16	0.1 (10)	C59—C60—C61—C62	-0.3 (9)
C8—C9—C23—C24	98.1 (5)	C60—C61—C62—C57	0.1 (9)
C4—C9—C23—C24	-82.4 (5)	C58—C57—C62—C61	0.5 (8)
C8—C9—C23—C30	-31.4 (6)	C50—C57—C62—C61	-178.1 (5)
C4—C9—C23—C30	148.1 (4)	C48—C49—C64—C71	-98.9 (5)
C30—C23—C24—C29	-104.4 (5)	C44—C49—C64—C71	76.9 (5)
C9—C23—C24—C29	129.2 (4)	C48—C49—C64—C65	28.7 (7)
C30—C23—C24—C25	75.3 (6)	C44—C49—C64—C65	-155.5 (5)
C9—C23—C24—C25	-51.1 (6)	C71—C64—C65—C70	19.2 (7)
C29—C24—C25—C26	2.7 (8)	C49—C64—C65—C70	-106.7 (6)
C23—C24—C25—C26	-177.0 (5)	C71—C64—C65—C66	-161.3 (4)
C24—C25—C26—C27	-0.9 (9)	C49—C64—C65—C66	72.8 (6)
C25—C26—C27—C28	-1.9 (9)	C70—C65—C66—C67	-1.9 (8)

C26—C27—C28—C29	2.9 (8)	C64—C65—C66—C67	178.5 (5)
C25—C24—C29—C28	-1.7 (7)	C65—C66—C67—C68	1.2 (9)
C23—C24—C29—C28	178.0 (4)	C66—C67—C68—C69	0.8 (10)
C27—C28—C29—C24	-1.1 (8)	C67—C68—C69—C70	-1.9 (10)
C24—C23—C30—C31	-15.8 (6)	C66—C65—C70—C69	0.8 (9)
C9—C23—C30—C31	111.5 (5)	C64—C65—C70—C69	-179.7 (5)
C24—C23—C30—C35	165.5 (4)	C68—C69—C70—C65	1.1 (10)
C9—C23—C30—C35	-67.1 (5)	C65—C64—C71—C72	-82.7 (6)
C35—C30—C31—C32	1.2 (8)	C49—C64—C71—C72	44.3 (6)
C23—C30—C31—C32	-177.5 (5)	C65—C64—C71—C76	97.9 (5)
C30—C31—C32—C33	-0.6 (9)	C49—C64—C71—C76	-135.1 (5)
C31—C32—C33—C34	0.2 (9)	C76—C71—C72—C73	0.2 (8)
C32—C33—C34—C35	-0.5 (8)	C64—C71—C72—C73	-179.3 (5)
C33—C34—C35—C30	1.1 (8)	C71—C72—C73—C74	1.1 (9)
C31—C30—C35—C34	-1.4 (7)	C72—C73—C74—C75	-1.6 (9)
C23—C30—C35—C34	177.3 (4)	C73—C74—C75—C76	0.9 (9)
C1—N2—C38—C39	-99.7 (5)	C74—C75—C76—C71	0.4 (8)
C3—N2—C38—C39	79.0 (5)	C72—C71—C76—C75	-0.9 (8)
N2—C38—C39—C40	60.0 (5)	C64—C71—C76—C75	178.5 (5)
C41—N4—C40—C39	86.3 (5)	C14—C13—C82—C12	0.4 (7)
C43—N4—C40—C39	-100.2 (5)	C11—C12—C82—C13	-2.5 (7)

$Au_2Br_2$4b

Au2—Br2	2.3971 (4)	C65—H65	1.0000
Au2—C42	1.990 (3)	C9—C4	1.396 (5)
Au1—Br1	2.3990 (4)	C6—H6	0.9500

Au1—C1	1.998 (3)	C6—C7	1.394 (5)
O2—C48	1.375 (4)	C6—C5	1.390 (5)
O2—C64	1.431 (4)	C4—C5	1.401 (5)
O1—C7	1.365 (4)	C2—C37	1.489 (5)
O1—C23	1.427 (4)	C25—C30	1.393 (5)
N3—C42	1.353 (4)	C25—C26	1.390 (5)
N3—C43	1.393 (4)	C71—H71	0.9500
N3—C45	1.442 (4)	C71—C70	1.399 (5)
N1—C1	1.357 (4)	C17—C22	1.394 (5)
N1—C4	1.429 (4)	C56—H56	0.9500
N1—C2	1.410 (4)	C56—C55	1.385 (6)
N4—C42	1.351 (4)	C56—C57	1.385 (5)
N4—C41	1.462 (4)	C39—H39A	0.9900
N4—C44	1.399 (4)	C39—H39B	0.9900
N2—C1	1.345 (4)	C39—C40	1.528 (5)
N2—C3	1.394 (5)	C37—H37A	0.9800
N2—C39	1.456 (4)	C37—H37B	0.9800
C72—C73	1.392 (5)	C37—H37C	0.9800
C72—C65	1.532 (4)	C74—H74	0.9500
C72—C77	1.405 (5)	C78—H78A	0.9800
C11—C16	1.391 (5)	C78—H78B	0.9800
C11—C12	1.391 (5)	C78—H78C	0.9800
C11—C10	1.528 (5)	C30—H30	0.9500
C24—H24	1.0000	C76—H76	0.9500
C24—C31	1.515 (5)	C76—C77	1.384 (5)
C24—C9	1.525 (5)	C40—H40A	0.9900
C24—C25	1.522 (5)	C40—H40B	0.9900
C48—C47	1.390 (5)	C77—H77	0.9500
C48—C49	1.394 (5)	C26—H26	0.9500
C58—C63	1.395 (5)	C26—C27	1.400 (5)
C58—C59	1.391 (5)	C28—H28	0.9500
C58—C51	1.523 (4)	C28—C27	1.367 (6)
C47—H47	0.9500	C22—H22	0.9500
C47—C46	1.399 (5)	C22—C21	1.398 (5)
C46—C45	1.396 (4)	C55—H55	0.9500
C46—C51	1.531 (4)	C55—C54	1.396 (6)
C50—C49	1.389 (5)	C36—H36	0.9500
C50—C45	1.403 (4)	C36—C35	1.380 (5)
C50—C65	1.531 (4)	C20—H20	0.9500
C41—H41A	0.9900	C20—C21	1.375 (6)
C41—H41B	0.9900	C20—C19	1.380 (6)

C41—C40	1.520 (5)	C60—H60	0.9500
C43—C44	1.359 (5)	C60—C61	1.380 (5)
C43—C78	1.488 (5)	C15—H15	0.9500
C66—C65	1.524 (5)	C15—C14	1.389 (6)
C66—C71	1.397 (5)	C51—H51	1.0000
C66—C67	1.398 (5)	C67—H67	0.9500
C8—H8	0.9500	C67—C68	1.392 (5)
C8—C9	1.399 (5)	C21—H21	0.9500
C8—C7	1.394 (5)	C62—H62	0.9500
C44—C79	1.492 (5)	C62—C61	1.393 (5)
C49—H49	0.9500	C57—H57	0.9500
C53—H53	0.9500	C79—H79A	0.9800
C53—C52	1.389 (5)	C79—H79B	0.9800
C53—C54	1.388 (5)	C79—H79C	0.9800
C73—H73	0.9500	C32—H32	0.9500
C73—C74	1.390 (5)	C32—C33	1.392 (6)
C63—H63	0.9500	C19—H19	0.9500
C63—C62	1.389 (5)	C64—H64A	0.9800
C29—H29	0.9500	C64—H64B	0.9800
C29—C30	1.391 (5)	C64—H64C	0.9800
C29—C28	1.381 (6)	C38—H38A	0.9800
C59—H59	0.9500	C38—H38B	0.9800
C59—C60	1.390 (5)	C38—H38C	0.9800
C31—C36	1.393 (5)	C54—H54	0.9500
C31—C32	1.400 (5)	C14—H14	0.9500
C16—H16	0.9500	C14—C13	1.377 (7)
C16—C15	1.389 (5)	C70—H70	0.9500
C12—H12	0.9500	C70—C69	1.374 (7)
C12—C13	1.389 (6)	C61—H61	0.9500
C52—C51	1.518 (4)	C23—H23A	0.9800
C52—C57	1.402 (5)	C23—H23B	0.9800
C75—H75	0.9500	C23—H23C	0.9800
C75—C74	1.392 (6)	C35—H35	0.9500
C75—C76	1.381 (6)	C35—C34	1.382 (7)
C18—H18	0.9500	C68—H68	0.9500
C18—C17	1.391 (5)	C68—C69	1.385 (7)
C18—C19	1.391 (5)	C33—H33	0.9500
C3—C2	1.345 (5)	C33—C34	1.387 (7)
C3—C38	1.493 (5)	C69—H69	0.9500
C10—H10	1.0000	C27—H27	0.9500
C10—C17	1.529 (5)	C13—H13	0.9500

C10—C5	1.531 (5)	C34—H34	0.9500
C42—Au2—Br2	174.92 (9)	N2—C39—H39B	109.7
C1—Au1—Br1	174.84 (10)	N2—C39—C40	109.9 (3)
C48—O2—C64	116.9 (3)	H39A—C39—H39B	108.2
C7—O1—C23	117.5 (3)	C40—C39—H39A	109.7
C42—N3—C43	111.4 (3)	C40—C39—H39B	109.7
C42—N3—C45	122.3 (3)	C2—C37—H37A	109.5
C43—N3—C45	125.9 (3)	C2—C37—H37B	109.5
C1—N1—C4	125.5 (3)	C2—C37—H37C	109.5
C1—N1—C2	110.0 (3)	H37A—C37—H37B	109.5
C2—N1—C4	124.3 (3)	H37A—C37—H37C	109.5
C42—N4—C41	123.9 (3)	H37B—C37—H37C	109.5
C42—N4—C44	111.0 (3)	O1—C7—C8	124.2 (3)
C44—N4—C41	125.0 (3)	O1—C7—C6	115.5 (3)
C1—N2—C3	110.9 (3)	C6—C7—C8	120.3 (3)
C1—N2—C39	122.5 (3)	C73—C74—C75	120.2 (3)
C3—N2—C39	125.7 (3)	C73—C74—H74	119.9
C73—C72—C65	122.4 (3)	C75—C74—H74	119.9
C73—C72—C77	118.7 (3)	C43—C78—H78A	109.5
C77—C72—C65	118.6 (3)	C43—C78—H78B	109.5
C16—C11—C10	123.0 (3)	C43—C78—H78C	109.5
C12—C11—C16	118.3 (3)	H78A—C78—H78B	109.5
C12—C11—C10	118.7 (3)	H78A—C78—H78C	109.5
N3—C42—Au2	125.9 (2)	H78B—C78—H78C	109.5
N4—C42—Au2	129.0 (3)	C29—C30—C25	121.6 (4)
N4—C42—N3	104.9 (3)	C29—C30—H30	119.2
C31—C24—H24	105.9	C25—C30—H30	119.2
C31—C24—C9	113.9 (3)	C75—C76—H76	120.1
C31—C24—C25	113.8 (3)	C75—C76—C77	119.8 (3)
C9—C24—H24	105.9	C77—C76—H76	120.1
C25—C24—H24	105.9	C41—C40—C39	111.5 (3)
C25—C24—C9	110.7 (3)	C41—C40—H40A	109.3
O2—C48—C47	124.3 (3)	C41—C40—H40B	109.3
O2—C48—C49	115.1 (3)	C39—C40—H40A	109.3
C47—C48—C49	120.6 (3)	C39—C40—H40B	109.3
C63—C58—C51	118.4 (3)	H40A—C40—H40B	108.0
C59—C58—C63	118.6 (3)	C72—C77—H77	119.6
C59—C58—C51	123.0 (3)	C76—C77—C72	120.9 (3)
C48—C47—H47	119.8	C76—C77—H77	119.6
C48—C47—C46	120.4 (3)	C25—C26—H26	119.9

C46—C47—H47	119.8	C25—C26—C27	120.2 (4)
C47—C46—C51	121.2 (3)	C27—C26—H26	119.9
C45—C46—C47	118.0 (3)	C29—C28—H28	119.9
C45—C46—C51	120.8 (3)	C27—C28—C29	120.2 (4)
N1—C1—Au1	129.3 (2)	C27—C28—H28	119.9
N2—C1—Au1	124.9 (2)	C17—C22—H22	120.0
N2—C1—N1	105.6 (3)	C17—C22—C21	119.9 (3)
C49—C50—C45	118.4 (3)	C21—C22—H22	120.0
C49—C50—C65	120.9 (3)	C56—C55—H55	120.2
C45—C50—C65	120.7 (3)	C56—C55—C54	119.7 (3)
N4—C41—H41A	109.4	C54—C55—H55	120.2
N4—C41—H41B	109.4	C6—C5—C10	121.3 (3)
N4—C41—C40	111.0 (3)	C6—C5—C4	118.3 (3)
H41A—C41—H41B	108.0	C4—C5—C10	120.4 (3)
C40—C41—H41A	109.4	C31—C36—H36	119.5
C40—C41—H41B	109.4	C35—C36—C31	121.1 (4)
N3—C43—C78	123.9 (3)	C35—C36—H36	119.5
C44—C43—N3	106.3 (3)	C21—C20—H20	120.1
C44—C43—C78	129.8 (3)	C21—C20—C19	119.8 (4)
C71—C66—C65	121.5 (3)	C19—C20—H20	120.1
C71—C66—C67	119.0 (3)	C59—C60—H60	119.6
C67—C66—C65	119.5 (3)	C61—C60—C59	120.7 (3)
C9—C8—H8	120.0	C61—C60—H60	119.6
C7—C8—H8	120.0	C16—C15—H15	119.8
C7—C8—C9	120.1 (3)	C14—C15—C16	120.4 (4)
N4—C44—C79	123.9 (3)	C14—C15—H15	119.8
C43—C44—N4	106.4 (3)	C58—C51—C46	111.8 (3)
C43—C44—C79	129.7 (3)	C58—C51—H51	106.5
C48—C49—H49	119.9	C46—C51—H51	106.5
C50—C49—C48	120.3 (3)	C52—C51—C58	113.5 (3)
C50—C49—H49	119.9	C52—C51—C46	111.6 (3)
C52—C53—H53	119.4	C52—C51—H51	106.5
C54—C53—H53	119.4	C66—C67—H67	119.8
C54—C53—C52	121.1 (3)	C68—C67—C66	120.3 (4)
C46—C45—N3	119.5 (3)	C68—C67—H67	119.8
C46—C45—C50	122.2 (3)	C22—C21—H21	119.7
C50—C45—N3	118.2 (3)	C20—C21—C22	120.6 (4)
C72—C73—H73	119.9	C20—C21—H21	119.7
C74—C73—C72	120.3 (3)	C63—C62—H62	119.9
C74—C73—H73	119.9	C63—C62—C61	120.2 (3)
C58—C63—H63	119.7	C61—C62—H62	119.9

C62—C63—C58	120.7 (3)	C52—C57—H57	119.8
C62—C63—H63	119.7	C56—C57—C52	120.4 (3)
C30—C29—H29	120.4	C56—C57—H57	119.8
C28—C29—H29	120.4	C44—C79—H79A	109.5
C28—C29—C30	119.2 (4)	C44—C79—H79B	109.5
C58—C59—H59	119.7	C44—C79—H79C	109.5
C60—C59—C58	120.6 (3)	H79A—C79—H79B	109.5
C60—C59—H59	119.7	H79A—C79—H79C	109.5
C36—C31—C24	122.4 (3)	H79B—C79—H79 C	109.5
C36—C31—C32	118.6 (3)	C31—C32—H32	120.0
C32—C31—C24	119.1 (3)	C33—C32—C31	120.0 (4)
C11—C16—H16	119.7	C33—C32—H32	120.0
C15—C16—C11	120.5 (4)	C18—C19—H19	119.9
C15—C16—H16	119.7	C20—C19—C18	120.1 (4)
C11—C12—H12	119.4	C20—C19—H19	119.9
C13—C12—C11	121.2 (4)	O2—C64—H64A	109.5
C13—C12—H12	119.4	O2—C64—H64B	109.5
C53—C52—C51	121.0 (3)	O2—C64—H64C	109.5
C53—C52—C57	118.6 (3)	H64A—C64—H64B	109.5
C57—C52—C51	120.3 (3)	H64A—C64—H64C	109.5
C74—C75—H75	119.9	H64B—C64—H64C	109.5
C76—C75—H75	119.9	C3—C38—H38A	109.5
C76—C75—C74	120.1 (3)	C3—C38—H38B	109.5
C17—C18—H18	119.7	C3—C38—H38C	109.5
C17—C18—C19	120.7 (4)	H38A—C38—H38B	109.5
C19—C18—H18	119.7	H38A—C38—H38C	109.5
N2—C3—C38	122.5 (3)	H38B—C38—H38C	109.5
C2—C3—N2	106.9 (3)	C53—C54—C55	119.7 (3)
C2—C3—C38	130.5 (3)	C53—C54—H54	120.2
C11—C10—H10	105.8	C55—C54—H54	120.2
C11—C10—C17	113.4 (3)	C15—C14—H14	120.3
C11—C10—C5	111.3 (3)	C13—C14—C15	119.5 (4)
C17—C10—H10	105.8	C13—C14—H14	120.3
C17—C10—C5	113.8 (3)	C71—C70—H70	119.7
C5—C10—H10	105.8	C69—C70—C71	120.6 (4)
C72—C65—H65	106.1	C69—C70—H70	119.7
C50—C65—C72	112.1 (3)	C60—C61—C62	119.1 (3)
C50—C65—H65	106.1	C60—C61—H61	120.4
C66—C65—C72	113.9 (3)	C62—C61—H61	120.4
C66—C65—C50	111.7 (3)	O1—C23—H23A	109.5
C66—C65—H65	106.1	O1—C23—H23B	109.5

C8—C9—C24	120.8 (3)	O1—C23—H23C	109.5
C4—C9—C24	120.6 (3)	H23A—C23—H23B	109.5
C4—C9—C8	118.5 (3)	H23A—C23—H23C	109.5
C7—C6—H6	119.7	H23B—C23—H23C	109.5
C5—C6—H6	119.7	C36—C35—H35	119.9
C5—C6—C7	120.6 (3)	C36—C35—C34	120.2 (4)
C9—C4—N1	118.7 (3)	C34—C35—H35	119.9
C9—C4—C5	121.8 (3)	C67—C68—H68	119.9
C5—C4—N1	119.4 (3)	C69—C68—C67	120.2 (4)
N1—C2—C37	122.5 (3)	C69—C68—H68	119.9
C3—C2—N1	106.5 (3)	C32—C33—H33	119.8
C3—C2—C37	131.0 (3)	C34—C33—C32	120.4 (4)
C30—C25—C24	117.9 (3)	C34—C33—H33	119.8
C26—C25—C24	124.0 (3)	C70—C69—C68	119.9 (4)
C26—C25—C30	118.0 (3)	C70—C69—H69	120.0
C66—C71—H71	120.1	C68—C69—H69	120.0
C66—C71—C70	119.9 (4)	C26—C27—H27	119.7
C70—C71—H71	120.1	C28—C27—C26	120.7 (4)
C18—C17—C10	118.4 (3)	C28—C27—H27	119.7
C18—C17—C22	118.8 (3)	C12—C13—H13	120.0
C22—C17—C10	122.8 (3)	C14—C13—C12	120.0 (4)
C55—C56—H56	119.8	C14—C13—H13	120.0
C57—C56—H56	119.8	C35—C34—C33	119.6 (4)
C57—C56—C55	120.5 (3)	C35—C34—H34	120.2
N2—C39—H39A	109.7	C33—C34—H34	120.2

O2—C48—C47—C46	179.1 (3)	C12—C11—C10—C17	-142.8 (3)
O2—C48—C49—C50	179.1 (3)	C12—C11—C10—C5	87.3 (4)
N3—C43—C44—N4	0.4 (4)	C52—C53—C54—C55	1.2 (5)
N3—C43—C44—C79	178.4 (4)	C75—C76—C77—C72	1.1 (6)
N1—C4—C5—C10	-5.5 (5)	C18—C17—C22—C21	0.6 (6)
N1—C4—C5—C6	175.2 (3)	C3—N2—C1—Au1	-174.2 (2)
N4—C41—C40—C39	-173.2 (3)	C3—N2—C1—N1	0.9 (4)
N2—C3—C2—N1	-0.6 (4)	C3—N2—C39—C40	87.0 (4)
N2—C3—C2—C37	179.4 (4)	C10—C11—C16—C15	-178.5 (3)
N2—C39—C40—C41	171.6 (3)	C10—C11—C12—C13	179.3 (3)
C72—C73—C74—	1.0 (6)	C10—C17—C22—	179.9 (3)

C75		C21	
C11—C16—C15—C14	0.1 (6)	C65—C72—C73—C74	172.5 (3)
C11—C12—C13—C14	-1.6 (6)	C65—C72—C77—C76	-173.8 (3)
C11—C10—C17—C18	90.5 (4)	C65—C50—C49—C48	179.0 (3)
C11—C10—C17—C22	-88.8 (4)	C65—C50—C45—N3	1.9 (4)
C11—C10—C5—C6	50.3 (4)	C65—C50—C45—C46	-175.6 (3)
C11—C10—C5—C4	-128.9 (3)	C65—C66—C71—C70	179.9 (3)
C42—N3—C43—C44	-0.9 (4)	C65—C66—C67—C68	179.9 (3)
C42—N3—C43—C78	178.5 (3)	C9—C24—C31—C36	-50.2 (5)
C42—N3—C45—C46	92.0 (4)	C9—C24—C31—C32	130.6 (3)
C42—N3—C45—C50	-85.5 (4)	C9—C24—C25—C30	-72.9 (4)
C42—N4—C41—C40	-100.3 (4)	C9—C24—C25—C26	103.6 (4)
C42—N4—C44—C43	0.3 (4)	C9—C8—C7—O1	179.3 (3)
C42—N4—C44—C79	-177.9 (3)	C9—C8—C7—C6	-2.6 (5)
C24—C31—C36—C35	-178.2 (4)	C9—C4—C5—C10	174.8 (3)
C24—C31—C32—C33	179.1 (4)	C9—C4—C5—C6	-4.5 (5)
C24—C9—C4—N1	10.6 (5)	C4—N1—C1—Au1	-1.1 (5)
C24—C9—C4—C5	-169.7 (3)	C4—N1—C1—N2	-175.9 (3)
C24—C25—C30—C29	177.2 (3)	C4—N1—C2—C3	175.9 (3)
C24—C25—C26—C27	-176.2 (4)	C4—N1—C2—C37	-4.2 (5)
C48—C47—C46—C45	2.3 (5)	C2—N1—C1—Au1	173.5 (2)
C48—C47—C46—C51	-175.4 (3)	C2—N1—C1—N2	-1.2 (4)
C58—C63—C62—C61	-1.5 (6)	C2—N1—C4—C9	97.8 (4)
C58—C59—C60—C61	-1.9 (6)	C2—N1—C4—C5	-81.9 (4)
C47—C48—C49—C50	-2.7 (5)	C25—C24—C31—C36	77.9 (4)
C47—C46—C45—N3	178.4 (3)	C25—C24—C31—C32	-101.3 (4)
C47—C46—C45—C50	-4.1 (5)	C25—C24—C9—C8	-19.0 (4)
C47—C46—C51—C58	19.7 (4)	C25—C24—C9—C4	156.9 (3)

C47—C46—C51—C52	-108.6 (3)	C25—C26—C27—C28	-1.3 (6)
C1—N1—C4—C9	-88.3 (4)	C71—C66—C65—C72	75.1 (4)
C1—N1—C4—C5	92.0 (4)	C71—C66—C65—C50	-53.2 (4)
C1—N1—C2—C3	1.2 (4)	C71—C66—C67—C68	0.4 (5)
C1—N1—C2—C37	-178.9 (3)	C71—C70—C69—C68	-0.8 (6)
C1—N2—C3—C2	-0.2 (4)	C17—C18—C19—C20	0.5 (6)
C1—N2—C3—C38	-179.3 (3)	C17—C10—C5—C6	-79.4 (4)
C1—N2—C39—C40	-81.3 (4)	C17—C10—C5—C4	101.4 (4)
C41—N4—C42—Au2	-8.0 (5)	C17—C22—C21—C20	0.0 (6)
C41—N4—C42—N3	177.3 (3)	C56—C55—C54—C53	-0.9 (5)
C41—N4—C44—C43	-177.8 (3)	C39—N2—C1—Au1	-4.4 (5)
C41—N4—C44—C79	4.0 (5)	C39—N2—C1—N1	170.7 (3)
C43—N3—C42—Au2	-173.8 (2)	C39—N2—C3—C2	-169.6 (3)
C43—N3—C42—N4	1.1 (3)	C39—N2—C3—C38	11.3 (5)
C43—N3—C45—C46	-95.6 (4)	C7—C8—C9—C24	173.3 (3)
C43—N3—C45—C50	86.8 (4)	C7—C8—C9—C4	-2.7 (5)
C66—C71—C70—C69	0.8 (6)	C7—C6—C5—C10	179.8 (3)
C66—C67—C68—C69	-0.4 (6)	C7—C6—C5—C4	-1.0 (5)
C8—C9—C4—N1	-173.4 (3)	C74—C75—C76—C77	-1.1 (6)
C8—C9—C4—C5	6.3 (5)	C78—C43—C44—N4	-179.0 (3)
C44—N4—C42—Au2	173.8 (2)	C78—C43—C44—C79	-1.0 (6)
C44—N4—C42—N3	-0.8 (4)	C30—C29—C28—C27	-0.9 (6)
C44—N4—C41—C40	77.6 (4)	C30—C25—C26—C27	0.2 (6)
C49—C48—C47—C46	1.0 (5)	C76—C75—C74—C73	0.0 (6)
C49—C50—C45—N3	180.0 (3)	C77—C72—C73—C74	-1.1 (5)
C49—C50—C45—C46	2.5 (5)	C77—C72—C65—C50	-52.4 (4)
C49—C50—C65—C72	-33.1 (4)	C77—C72—C65—C66	179.5 (3)
C49—C50—C65—C66	96.2 (4)	C26—C25—C30—C29	0.5 (5)

C53—C52—C51—C58	102.3 (4)	C28—C29—C30—C25	-0.1 (6)
C53—C52—C51—C46	-130.3 (3)	C55—C56—C57—C52	0.2 (5)
C53—C52—C57—C56	0.1 (5)	C5—C10—C17—C18	-140.9 (3)
C45—N3—C42—Au2	-0.4 (4)	C5—C10—C17—C22	39.8 (5)
C45—N3—C42—N4	174.4 (3)	C5—C6—C7—O1	-177.2 (3)
C45—N3—C43—C44	-174.0 (3)	C5—C6—C7—C8	4.5 (5)
C45—N3—C43—C78	5.5 (5)	C36—C31—C32—C33	-0.2 (6)
C45—C46—C51—C58	-158.0 (3)	C36—C35—C34—C33	0.0 (7)
C45—C46—C51—C52	73.7 (4)	C15—C14—C13—C12	1.9 (6)
C45—C50—C49—C48	1.0 (5)	C51—C58—C63—C62	-175.9 (3)
C45—C50—C65—C72	144.9 (3)	C51—C58—C59—C60	177.5 (4)
C45—C50—C65—C66	-85.8 (4)	C51—C46—C45—N3	-3.8 (4)
C73—C72—C65—C50	133.9 (3)	C51—C46—C45—C50	173.6 (3)
C73—C72—C65—C66	5.9 (5)	C51—C52—C57—C56	177.1 (3)
C73—C72—C77—C76	0.0 (5)	C67—C66—C65—C72	-104.5 (4)
C63—C58—C59—C60	-0.5 (6)	C67—C66—C65—C50	127.2 (3)
C63—C58—C51—C46	60.6 (4)	C67—C66—C71—C70	-0.6 (5)
C63—C58—C51—C52	-172.1 (3)	C67—C68—C69—C70	0.6 (6)
C63—C62—C61—C60	-0.9 (6)	C21—C20—C19—C18	0.1 (6)
C29—C28—C27—C26	1.6 (6)	C57—C52—C51—C58	-74.7 (4)
C59—C58—C63—C62	2.2 (5)	C57—C52—C51—C46	52.7 (4)
C59—C58—C51—C46	-117.4 (4)	C57—C56—C55—C54	0.2 (5)
C59—C58—C51—C52	9.9 (5)	C32—C31—C36—C35	1.1 (6)
C59—C60—C61—C62	2.6 (6)	C32—C33—C34—C35	0.9 (8)
C31—C24—C9—C8	110.6 (4)	C19—C18—C17—C10	179.9 (3)
C31—C24—C9—C4	-73.4 (4)	C19—C18—C17—	-0.8 (5)

		C22	
C31—C24—C25—C30	157.4 (3)	C19—C20—C21—C22	-0.4 (6)
C31—C24—C25—C26	-26.2 (5)	C64—O2—C48—C47	6.1 (5)
C31—C36—C35—C34	-1.0 (7)	C64—O2—C48—C49	-175.7 (3)
C31—C32—C33—C34	-0.8 (7)	C38—C3—C2—N1	178.4 (4)
C16—C11—C12—C13	0.5 (5)	C38—C3—C2—C37	-1.5 (7)
C16—C11—C10—C17	35.9 (5)	C54—C53—C52—C51	-177.8 (3)
C16—C11—C10—C5	-94.0 (4)	C54—C53—C52—C57	-0.7 (5)
C16—C15—C14—C13	-1.1 (6)	C23—O1—C7—C8	-8.2 (5)
C12—C11—C16—C15	0.2 (5)	C23—O1—C7—C6	173.6 (3)

$Au_2Br_2$4c

Au2—Br2	2.3926 (12)	C32—C54	1.501 (13)
Au2—C30	1.991 (9)	C25—H25A	0.9800
Au1—Br1	2.4060 (12)	C25—H25B	0.9800
Au1—C1	1.980 (10)	C25—H25C	0.9800
N2—C1	1.345 (12)	C9—C12	1.546 (12)
N2—C27	1.475 (11)	C24—H24	0.9500
N2—C3	1.396 (11)	C24—C23	1.346 (14)
N1—C1	1.347 (11)	C29—H29A	0.9900
N1—C2	1.438 (12)	C29—H29B	0.9900
N1—C4	1.436 (12)	C55—C37	1.386 (13)
N3—C30	1.363 (11)	C55—C52	1.505 (13)
N3—C33	1.433 (10)	C54—H54A	0.9800
N3—C31	1.386 (12)	C54—H54B	0.9800
N4—C30	1.346 (10)	C54—H54C	0.9800
N4—C32	1.392 (13)	C12—H12	1.0000
N4—C29	1.481 (11)	C12—C13	1.520 (13)
C45—C38	1.521 (12)	C40—H40	0.9500
C45—C46	1.396 (13)	C40—C41	1.349 (12)
C45—C50	1.369 (12)	C26—H26A	0.9800
C44—H44	0.9500	C26—H26B	0.9800
C44—C39	1.384 (11)	C26—H26C	0.9800
C44—C43	1.381 (12)	C41—H41	0.9500
C39—C38	1.505 (12)	C49—H49	0.9500

C39—C40	1.393 (11)	C49—C48	1.377 (18)
C38—H38	1.0000	C5—C10	1.512 (15)
C38—C34	1.534 (11)	C5—C6	1.396 (15)
C35—H35	0.9500	C37—H37	0.9500
C35—C34	1.386 (11)	C23—H23	0.9500
C35—C36	1.389 (12)	C23—C22	1.376 (16)
C27—H27A	0.9900	C52—H52A	0.9800
C27—H27B	0.9900	C52—H52B	0.9800
C27—C28	1.508 (11)	C52—H52C	0.9800
C2—C3	1.332 (13)	C10—H10A	0.9800
C2—C25	1.484 (13)	C10—H10B	0.9800
C34—C33	1.386 (12)	C10—H10C	0.9800
C46—H46	0.9500	C7—C6	1.370 (16)
C46—C47	1.378 (13)	C7—C11	1.506 (16)
C33—C55	1.421 (12)	C20—H20	0.9500
C3—C26	1.512 (14)	C20—C21	1.386 (14)
C31—C53	1.483 (14)	C13—C18	1.396 (15)
C31—C32	1.345 (13)	C13—C14	1.376 (15)
C42—H42	0.9500	C47—H47	0.9500
C42—C43	1.378 (14)	C47—C48	1.374 (17)
C42—C41	1.409 (15)	C21—H21	0.9500
C50—H50	0.9500	C21—C22	1.415 (16)
C50—C49	1.398 (16)	C51—H51A	0.9800
C19—C24	1.406 (13)	C51—H51B	0.9800
C19—C12	1.507 (13)	C51—H51C	0.9800
C19—C20	1.393 (14)	C18—H18	0.9500
C28—H28A	0.9900	C18—C17	1.391 (15)
C28—H28B	0.9900	C22—H22	0.9500
C28—C29	1.529 (11)	C48—H48	0.9500
C43—H43	0.9500	C17—H17	0.9500
C53—H53A	0.9800	C17—C16	1.387 (19)
C53—H53B	0.9800	C14—H14	0.9500
C53—H53C	0.9800	C14—C15	1.399 (17)
C4—C9	1.388 (12)	C6—H6	0.9500
C4—C5	1.406 (13)	C16—H16	0.9500
C8—H8	0.9500	C16—C15	1.38 (2)
C8—C9	1.377 (13)	C15—H15	0.9500
C8—C7	1.406 (14)	C11—H11A	0.9800
C36—C37	1.366 (14)	C11—H11B	0.9800
C36—C51	1.542 (12)	C11—H11C	0.9800

C30—Au2—Br2	175.7 (2)	C23—C24—H24	118.7
C1—Au1—Br1	178.7 (3)	N4—C29—C28	112.7 (7)
C1—N2—C27	122.3 (8)	N4—C29—H29A	109.1
C1—N2—C3	111.5 (8)	N4—C29—H29B	109.1
C3—N2—C27	126.0 (8)	C28—C29—H29A	109.1
C1—N1—C2	110.3 (8)	C28—C29—H29B	109.1
C1—N1—C4	124.6 (8)	H29A—C29—H29B	107.8
C4—N1—C2	125.0 (7)	C33—C55—C52	120.9 (8)
C30—N3—C33	123.1 (8)	C37—C55—C33	117.2 (9)
C30—N3—C31	111.0 (7)	C37—C55—C52	121.9 (9)
C31—N3—C33	125.9 (7)	C32—C54—H54A	109.5
C30—N4—C32	111.0 (8)	C32—C54—H54B	109.5
C30—N4—C29	124.1 (9)	C32—C54—H54C	109.5
C32—N4—C29	124.3 (8)	H54A—C54—H54B	109.5
C46—C45—C38	122.7 (8)	H54A—C54—H54C	109.5
C50—C45—C38	120.7 (9)	H54B—C54—H54C	109.5
C50—C45—C46	116.6 (9)	C19—C12—C9	111.0 (7)
C39—C44—H44	119.1	C19—C12—H12	106.6
C43—C44—H44	119.1	C19—C12—C13	114.6 (8)
C43—C44—C39	121.8 (8)	C9—C12—H12	106.6
C44—C39—C38	121.6 (7)	C13—C12—C9	110.9 (7)
C44—C39—C40	117.7 (8)	C13—C12—H12	106.6
C40—C39—C38	120.7 (7)	C39—C40—H40	119.3
N2—C1—Au1	129.4 (6)	C41—C40—C39	121.5 (9)
N2—C1—N1	105.2 (8)	C41—C40—H40	119.3
N1—C1—Au1	125.4 (8)	C3—C26—H26A	109.5
C45—C38—H38	105.9	C3—C26—H26B	109.5
C45—C38—C34	113.0 (7)	C3—C26—H26C	109.5
C39—C38—C45	113.7 (7)	H26A—C26—H26B	109.5
C39—C38—H38	105.9	H26A—C26—H26C	109.5
C39—C38—C34	111.6 (7)	H26B—C26—H26C	109.5
C34—C38—H38	105.9	C42—C41—H41	119.7
N3—C30—Au2	124.6 (6)	C40—C41—C42	120.5 (9)
N4—C30—Au2	130.9 (7)	C40—C41—H41	119.7
N4—C30—N3	104.5 (8)	C50—C49—H49	119.3
C34—C35—H35	119.3	C48—C49—C50	121.4 (12)
C34—C35—C36	121.4 (8)	C48—C49—H49	119.3
C36—C35—H35	119.3	C4—C5—C10	120.5 (10)
N2—C27—H27A	109.7	C6—C5—C4	117.4 (10)
N2—C27—H27B	109.7	C6—C5—C10	122.1 (10)
N2—C27—C28	109.7 (7)	C36—C37—C55	121.8 (9)

H27A—C27—H27B	108.2	C36—C37—H37	119.1
C28—C27—H27A	109.7	C55—C37—H37	119.1
C28—C27—H27B	109.7	C24—C23—H23	120.5
N1—C2—C25	120.6 (9)	C24—C23—C22	119.0 (11)
C3—C2—N1	105.7 (8)	C22—C23—H23	120.5
C3—C2—C25	133.8 (9)	C55—C52—H52A	109.5
C35—C34—C38	122.4 (8)	C55—C52—H52B	109.5
C33—C34—C38	119.7 (7)	C55—C52—H52C	109.5
C33—C34—C35	117.9 (8)	H52A—C52—H52B	109.5
C45—C46—H46	118.7	H52A—C52—H52C	109.5
C47—C46—C45	122.6 (10)	H52B—C52—H52C	109.5
C47—C46—H46	118.7	C5—C10—H10A	109.5
C34—C33—N3	120.5 (7)	C5—C10—H10B	109.5
C34—C33—C55	121.8 (8)	C5—C10—H10C	109.5
C55—C33—N3	117.6 (8)	H10A—C10—H10B	109.5
N2—C3—C26	122.4 (9)	H10A—C10—H10C	109.5
C2—C3—N2	107.4 (9)	H10B—C10—H10C	109.5
C2—C3—C26	130.2 (9)	C8—C7—C11	120.6 (11)
N3—C31—C53	123.3 (8)	C6—C7—C8	117.6 (11)
C32—C31—N3	106.5 (9)	C6—C7—C11	121.8 (11)
C32—C31—C53	130.2 (10)	C19—C20—H20	120.4
C43—C42—H42	120.6	C21—C20—C19	119.2 (10)
C43—C42—C41	118.8 (9)	C21—C20—H20	120.4
C41—C42—H42	120.6	C18—C13—C12	123.0 (10)
C45—C50—H50	119.5	C14—C13—C12	119.4 (10)
C45—C50—C49	121.1 (11)	C14—C13—C18	117.6 (10)
C49—C50—H50	119.5	C46—C47—H47	119.9
C24—C19—C12	118.8 (9)	C48—C47—C46	120.2 (11)
C20—C19—C24	118.8 (9)	C48—C47—H47	119.9
C20—C19—C12	122.4 (8)	C20—C21—H21	120.0
C27—C28—H28A	108.9	C20—C21—C22	119.9 (12)
C27—C28—H28B	108.9	C22—C21—H21	120.0
C27—C28—C29	113.5 (7)	C36—C51—H51A	109.5
H28A—C28—H28B	107.7	C36—C51—H51B	109.5
C29—C28—H28A	108.9	C36—C51—H51C	109.5
C29—C28—H28B	108.9	H51A—C51—H51B	109.5
C44—C43—H43	120.1	H51A—C51—H51C	109.5
C42—C43—C44	119.7 (9)	H51B—C51—H51C	109.5
C42—C43—H43	120.1	C13—C18—H18	119.0
C31—C53—H53A	109.5	C17—C18—C13	122.1 (12)
C31—C53—H53B	109.5	C17—C18—H18	119.0

C31—C53—H53C	109.5	C23—C22—C21	120.4 (10)
H53A—C53—H53B	109.5	C23—C22—H22	119.8
H53A—C53—H53C	109.5	C21—C22—H22	119.8
H53B—C53—H53C	109.5	C49—C48—H48	120.9
C9—C4—N1	119.8 (8)	C47—C48—C49	118.1 (11)
C9—C4—C5	121.9 (10)	C47—C48—H48	120.9
C5—C4—N1	118.3 (9)	C18—C17—H17	120.3
C9—C8—H8	118.6	C16—C17—C18	119.5 (14)
C9—C8—C7	122.7 (10)	C16—C17—H17	120.3
C7—C8—H8	118.6	C13—C14—H14	119.4
C35—C36—C51	120.6 (9)	C13—C14—C15	121.1 (14)
C37—C36—C35	119.7 (8)	C15—C14—H14	119.4
C37—C36—C51	119.7 (8)	C5—C6—H6	118.7
N4—C32—C54	124.4 (9)	C7—C6—C5	122.6 (10)
C31—C32—N4	106.9 (8)	C7—C6—H6	118.7
C31—C32—C54	128.3 (10)	C17—C16—H16	120.4
C2—C25—H25A	109.5	C15—C16—C17	119.2 (13)
C2—C25—H25B	109.5	C15—C16—H16	120.4
C2—C25—H25C	109.5	C14—C15—H15	119.7
H25A—C25—H25B	109.5	C16—C15—C14	120.6 (14)
H25A—C25—H25C	109.5	C16—C15—H15	119.7
H25B—C25—H25C	109.5	C7—C11—H11A	109.5
C4—C9—C12	120.8 (8)	C7—C11—H11B	109.5
C8—C9—C4	117.7 (9)	C7—C11—H11C	109.5
C8—C9—C12	121.5 (9)	H11A—C11—H11B	109.5
C19—C24—H24	118.7	H11A—C11—H11C	109.5
C23—C24—C19	122.6 (11)	H11B—C11—H11C	109.5

N2—C27—C28—C29	176.2 (8)	C31—N3—C33—C34	-96.6 (11)
N1—C2—C3—N2	-0.1 (10)	C31—N3—C33—C55	81.8 (11)
N1—C2—C3—C26	179.9 (9)	C50—C45—C38—C39	-172.6 (7)
N1—C4—C9—C8	176.3 (8)	C50—C45—C38—C34	58.8 (10)
N1—C4—C9—C12	-2.7 (12)	C50—C45—C46—C47	1.1 (13)
N1—C4—C5—C10	2.4 (14)	C50—C49—C48—C47	-1.7 (16)
N1—C4—C5—C6	-177.0 (9)	C19—C24—C23—C22	1.0 (16)
N3—C33—C55—C37	-173.9 (10)	C19—C12—C13—C18	19.3 (14)
N3—C33—C55—C52	3.8 (15)	C19—C12—C13—	-161.0 (10)

		C14	
N3—C31—C32—N4	-0.6 (10)	C19—C20—C21—C22	0.1 (16)
N3—C31—C32—C54	-173.2 (8)	C43—C44—C39—C38	179.8 (8)
C45—C38—C34—C35	19.2 (12)	C43—C44—C39—C40	1.0 (13)
C45—C38—C34—C33	-158.7 (8)	C43—C42—C41—C40	0.9 (16)
C45—C46—C47—C48	-1.9 (15)	C53—C31—C32—N4	-179.2 (9)
C45—C50—C49—C48	1.0 (16)	C53—C31—C32—C54	8.2 (17)
C44—C39—C38—C45	-82.3 (9)	C4—N1—C1—Au1	3.0 (12)
C44—C39—C38—C34	47.1 (10)	C4—N1—C1—N2	-176.3 (7)
C44—C39—C40—C41	0.2 (14)	C4—N1—C2—C3	176.2 (8)
C39—C44—C43—C42	-1.3 (15)	C4—N1—C2—C25	-2.4 (13)
C39—C38—C34—C35	-110.5 (9)	C4—C9—C12—C19	83.6 (11)
C39—C38—C34—C33	71.6 (10)	C4—C9—C12—C13	-147.7 (9)
C39—C40—C41—C42	-1.2 (16)	C4—C5—C6—C7	-0.6 (17)
C1—N2—C27—C28	-80.0 (10)	C8—C9—C12—C19	-95.4 (10)
C1—N2—C3—C2	-0.2 (10)	C8—C9—C12—C13	33.3 (12)
C1—N2—C3—C26	179.8 (8)	C8—C7—C6—C5	-1.0 (18)
C1—N1—C2—C3	0.4 (10)	C36—C35—C34—C38	-179.4 (9)
C1—N1—C2—C25	-178.3 (8)	C36—C35—C34—C33	-1.4 (14)
C1—N1—C4—C9	-93.6 (11)	C32—N4—C30—Au2	-179.4 (6)
C1—N1—C4—C5	86.5 (11)	C32—N4—C30—N3	-1.7 (9)
C38—C45—C46—C47	-178.8 (8)	C32—N4—C29—C28	-67.5 (11)
C38—C45—C50—C49	179.3 (8)	C25—C2—C3—N2	178.3 (10)
C38—C39—C40—C41	-178.6 (9)	C25—C2—C3—C26	-1.7 (18)
C38—C34—C33—N3	-6.3 (13)	C9—C4—C5—C10	-177.5 (9)
C38—C34—C33—C55	175.3 (9)	C9—C4—C5—C6	3.1 (14)
C30—N3—C33—C34	84.8 (11)	C9—C8—C7—C6	0.3 (17)
C30—N3—C33—C55	-96.8 (11)	C9—C8—C7—C11	-178.2 (12)

C30—N3—C31—C53	178.2 (8)	C9—C12—C13—C18	-107.4 (11)
C30—N3—C31—C32	-0.5 (10)	C9—C12—C13—C14	72.3 (12)
C30—N4—C32—C31	1.5 (10)	C24—C19—C12—C9	-137.1 (9)
C30—N4—C32—C54	174.5 (8)	C24—C19—C12—C13	96.3 (10)
C30—N4—C29—C28	103.3 (10)	C24—C19—C20—C21	-1.8 (14)
C35—C34—C33—N3	175.7 (8)	C24—C23—C22—C21	-2.7 (17)
C35—C34—C33—C55	-2.7 (14)	C29—N4—C30—Au2	8.8 (12)
C35—C36—C37—C55	-1.6 (18)	C29—N4—C30—N3	-173.6 (7)
C27—N2—C1—Au1	-4.1 (12)	C29—N4—C32—C31	173.3 (8)
C27—N2—C1—N1	175.1 (7)	C29—N4—C32—C54	-13.7 (13)
C27—N2—C3—C2	-174.7 (8)	C12—C19—C24—C23	-177.8 (9)
C27—N2—C3—C26	5.3 (13)	C12—C19—C20—C21	177.3 (9)
C27—C28—C29—N4	-53.4 (12)	C12—C13—C18—C17	178.5 (10)
C2—N1—C1—Au1	178.8 (6)	C12—C13—C14—C15	-178.5 (10)
C2—N1—C1—N2	-0.4 (10)	C40—C39—C38—C45	96.5 (9)
C2—N1—C4—C9	91.2 (10)	C40—C39—C38—C34	-134.2 (8)
C2—N1—C4—C5	-88.7 (11)	C41—C42—C43—C44	0.3 (16)
C34—C35—C36—C37	3.6 (15)	C5—C4—C9—C8	-3.8 (13)
C34—C35—C36—C51	-178.4 (9)	C5—C4—C9—C12	177.2 (8)
C34—C33—C55—C37	4.5 (16)	C52—C55—C37—C36	-179.9 (11)
C34—C33—C55—C52	-177.8 (10)	C10—C5—C6—C7	-180.0 (12)
C46—C45—C38—C39	7.3 (11)	C7—C8—C9—C4	2.0 (14)
C46—C45—C38—C34	-121.3 (9)	C7—C8—C9—C12	-178.9 (10)
C46—C45—C50—C49	-0.6 (13)	C20—C19—C24—C23	1.2 (14)
C46—C47—C48—C49	2.1 (16)	C20—C19—C12—C9	43.9 (12)
C33—N3—C30—Au2	-2.1 (11)	C20—C19—C12—C13	-82.7 (11)

C33—N3—C30—N4	-179.9 (7)	C20—C21—C22—C23	2.1 (17)
C33—N3—C31—C53	-0.5 (14)	C13—C18—C17—C16	0.7 (18)
C33—N3—C31—C32	-179.2 (8)	C13—C14—C15—C16	-1 (2)
C33—C55—C37—C36	-2.3 (18)	C51—C36—C37—C55	-179.7 (11)
C3—N2—C1—Au1	-178.9 (6)	C18—C13—C14—C15	1.2 (17)
C3—N2—C1—N1	0.4 (10)	C18—C17—C16—C15	0 (2)
C3—N2—C27—C28	93.9 (10)	C17—C16—C15—C14	0 (2)
C31—N3—C30—Au2	179.2 (6)	C14—C13—C18—C17	-1.2 (16)
C31—N3—C30—N4	1.4 (9)	C11—C7—C6—C5	177.4 (12)

$Au_2Br_2$4d

Au1—Br1	2.4166 (4)	C23—H23B	0.9800
Au1—C1	1.994 (3)	C23—H23C	0.9800
Au2—Br2	2.3967 (4)	C46—C51	1.388 (5)
Au2—C43	1.990 (4)	C34—H34	0.9500
N2—C3	1.403 (4)	C34—C35	1.384 (5)
N2—C1	1.351 (4)	C57—H57	0.9500
N2—C39	1.470 (4)	C57—C56	1.392 (6)
N4—C43	1.336 (4)	C67—C66	1.527 (5)
N4—C45	1.398 (5)	C67—C68	1.404 (5)
N4—C42	1.475 (4)	C67—C72	1.383 (5)
N3—C43	1.358 (4)	C78—H78	0.9500
N3—C44	1.397 (4)	C78—C77	1.396 (5)
N3—C46	1.443 (4)	C30—H30	0.9500
N1—C2	1.398 (4)	C30—C29	1.393 (5)
N1—C1	1.353 (4)	C36—H36	0.9500
N1—C4	1.449 (4)	C36—C35	1.387 (5)
C32—H32	0.9500	C51—C66	1.536 (5)
C32—C31	1.391 (5)	C51—C50	1.401 (5)
C32—C33	1.387 (5)	C60—H60	0.9500
C47—C52	1.524 (5)	C16—H16	0.9500
C47—C46	1.406 (5)	C16—C15	1.403 (5)
C47—C48	1.392 (5)	C10—H10	1.0000
C8—H8	0.9500	C10—C17	1.534 (5)

C8—C9	1.399 (5)	C55—H55	0.9500
C8—C7	1.392 (5)	C55—C54	1.389 (5)
C52—H52	1.0000	C55—C56	1.380 (6)
C52—C59	1.534 (5)	C48—H48	0.9500
C52—C53	1.523 (5)	C53—C54	1.393 (5)
C9—C4	1.401 (5)	C66—H66	1.0000
C9—C24	1.526 (4)	C68—H68	0.9500
C2—C3	1.351 (5)	C68—C69	1.380 (6)
C2—C37	1.490 (5)	C50—H50	0.9500
C31—C24	1.533 (5)	C38—H38A	0.9800
C31—C36	1.391 (5)	C38—H38B	0.9800
C3—C38	1.490 (5)	C38—H38C	0.9800
C5—C4	1.395 (5)	C26—H26	0.9500
C5—C6	1.402 (5)	C26—C27	1.391 (5)
C5—C10	1.527 (5)	C22—H22	0.9500
C58—H58	0.9500	C22—C21	1.390 (5)
C58—C57	1.387 (5)	C22—C17	1.405 (5)
C58—C53	1.389 (5)	C64—H64	0.9500
C79—H79A	0.9800	C21—H21	0.9500
C79—H79B	0.9800	C21—C20	1.393 (6)
C79—H79C	0.9800	C42—H42A	0.9900
C79—C44	1.492 (5)	C42—H42B	0.9900
C61—H61	0.9500	C40—H40A	0.9900
C61—C62	1.382 (5)	C40—H40B	0.9900
C61—C60	1.389 (5)	C40—C39	1.521 (5)
C18—H18	0.9500	C37—H37A	0.9800
C18—C19	1.396 (5)	C37—H37B	0.9800
C18—C17	1.381 (5)	C37—H37C	0.9800
C24—H24	1.0000	C35—H35	0.9500
C24—C25	1.535 (4)	C19—H19	0.9500
C6—H6	0.9500	C19—C20	1.383 (6)
C6—C7	1.393 (5)	C39—H39A	0.9900
C44—C45	1.354 (5)	C39—H39B	0.9900
C45—C80	1.487 (5)	C20—H20	0.9500
C73—C78	1.387 (5)	C74—H74	0.9500
C73—C66	1.525 (5)	C74—C75	1.377 (6)
C73—C74	1.403 (5)	C29—H29	0.9500
C59—C60	1.391 (5)	C72—H72	0.9500
C59—C64	1.399 (5)	C72—C71	1.389 (6)
C33—H33	0.9500	C65—H65A	0.9800
C33—C34	1.381 (5)	C65—H65B	0.9800

C63—H63	0.9500	C65—H65C	0.9800
C63—C62	1.400 (5)	C27—H27	0.9500
C63—C64	1.388 (5)	C13—H13	0.9500
C41—H41A	0.9900	C13—C14	1.390 (6)
C41—H41B	0.9900	C54—H54	0.9500
C41—C42	1.545 (5)	C56—H56	0.9500
C41—C40	1.517 (5)	C77—H77	0.9500
C25—C30	1.382 (5)	C77—C76	1.382 (6)
C25—C26	1.392 (5)	C15—H15	0.9500
C7—C23	1.512 (5)	C15—C14	1.375 (6)
C49—C48	1.397 (5)	C69—H69	0.9500
C49—C50	1.395 (5)	C69—C70	1.385 (6)
C49—C65	1.505 (5)	C75—H75	0.9500
C11—C12	1.396 (5)	C75—C76	1.390 (6)
C11—C16	1.393 (5)	C80—H80A	0.9800
C11—C10	1.527 (5)	C80—H80B	0.9800
C62—H62	0.9500	C80—H80C	0.9800
C12—H12	0.9500	C71—H71	0.9500
C12—C13	1.386 (6)	C71—C70	1.374 (7)
C28—H28	0.9500	C70—H70	0.9500
C28—C29	1.385 (6)	C76—H76	0.9500
C28—C27	1.383 (6)	C14—H14	0.9500
C23—H23A	0.9800		

C1—Au1—Br1	175.53 (9)	C35—C36—H36	119.5
C43—Au2—Br2	174.86 (10)	C46—C51—C66	120.3 (3)
C3—N2—C39	125.0 (3)	C46—C51—C50	118.3 (3)
C1—N2—C3	110.7 (3)	C50—C51—C66	121.2 (3)
C1—N2—C39	124.1 (3)	C61—C60—C59	120.0 (3)
C43—N4—C45	111.2 (3)	C61—C60—H60	120.0
C43—N4—C42	126.1 (3)	C59—C60—H60	120.0
C45—N4—C42	122.7 (3)	C11—C16—H16	119.8
C43—N3—C44	110.7 (3)	C11—C16—C15	120.4 (4)
C43—N3—C46	124.8 (3)	C15—C16—H16	119.8
C44—N3—C46	124.5 (3)	C5—C10—H10	106.0
C2—N1—C4	122.6 (3)	C5—C10—C17	112.5 (3)
C1—N1—C2	110.9 (3)	C11—C10—C5	112.1 (3)
C1—N1—C4	126.0 (3)	C11—C10—H10	106.0
C31—C32—H32	119.7	C11—C10—C17	113.6 (3)
C33—C32—H32	119.7	C17—C10—H10	106.0
C33—C32—C31	120.5 (3)	C54—C55—H55	119.6

C46—C47—C52	120.4 (3)	C56—C55—H55	119.6
C48—C47—C52	122.0 (3)	C56—C55—C54	120.7 (3)
C48—C47—C46	117.5 (3)	C47—C48—C49	122.0 (3)
C9—C8—H8	119.2	C47—C48—H48	119.0
C7—C8—H8	119.2	C49—C48—H48	119.0
C7—C8—C9	121.7 (3)	C58—C53—C52	121.6 (3)
C47—C52—H52	105.9	C58—C53—C54	118.4 (3)
C47—C52—C59	111.2 (3)	C54—C53—C52	120.0 (3)
C59—C52—H52	105.9	C73—C66—C67	114.0 (3)
C53—C52—C47	112.0 (3)	C73—C66—C51	113.7 (3)
C53—C52—H52	105.9	C73—C66—H66	106.3
C53—C52—C59	115.1 (3)	C67—C66—C51	109.6 (3)
C8—C9—C4	117.5 (3)	C67—C66—H66	106.3
C8—C9—C24	121.9 (3)	C51—C66—H66	106.3
C4—C9—C24	120.5 (3)	C67—C68—H68	119.8
N1—C2—C37	123.0 (3)	C69—C68—C67	120.5 (4)
C3—C2—N1	106.5 (3)	C69—C68—H68	119.8
C3—C2—C37	130.5 (3)	C49—C50—C51	121.2 (3)
C32—C31—C24	122.5 (3)	C49—C50—H50	119.4
C32—C31—C36	118.5 (3)	C51—C50—H50	119.4
C36—C31—C24	119.1 (3)	C3—C38—H38A	109.5
N2—C3—C38	123.8 (3)	C3—C38—H38B	109.5
C2—C3—N2	106.6 (3)	C3—C38—H38C	109.5
C2—C3—C38	129.2 (3)	H38A—C38—H38B	109.5
N2—C1—Au1	128.7 (2)	H38A—C38—H38C	109.5
N2—C1—N1	105.3 (3)	H38B—C38—H38C	109.5
N1—C1—Au1	125.8 (2)	C25—C26—H26	119.6
C4—C5—C6	117.6 (3)	C27—C26—C25	120.8 (3)
C4—C5—C10	120.9 (3)	C27—C26—H26	119.6
C6—C5—C10	121.5 (3)	C21—C22—H22	119.8
C57—C58—H58	119.4	C21—C22—C17	120.4 (3)
C57—C58—C53	121.2 (3)	C17—C22—H22	119.8
C53—C58—H58	119.4	C59—C64—H64	119.6
H79A—C79—H79B	109.5	C63—C64—C59	120.8 (3)
H79A—C79—H79C	109.5	C63—C64—H64	119.6
H79B—C79—H79C	109.5	C22—C21—H21	119.7
C44—C79—H79A	109.5	C22—C21—C20	120.6 (4)
C44—C79—H79B	109.5	C20—C21—H21	119.7
C44—C79—H79C	109.5	N4—C42—C41	113.0 (3)
C62—C61—H61	119.4	N4—C42—H42A	109.0
C62—C61—C60	121.1 (3)	N4—C42—H42B	109.0

C60—C61—H61	119.4	C41—C42—H42A	109.0
C9—C4—N1	118.8 (3)	C41—C42—H42B	109.0
C5—C4—N1	118.6 (3)	H42A—C42—H42B	107.8
C5—C4—C9	122.6 (3)	C41—C40—H40A	108.8
C19—C18—H18	119.7	C41—C40—H40B	108.8
C17—C18—H18	119.7	C41—C40—C39	113.8 (3)
C17—C18—C19	120.7 (3)	H40A—C40—H40B	107.7
C9—C24—C31	111.1 (3)	C39—C40—H40A	108.8
C9—C24—H24	106.4	C39—C40—H40B	108.8
C9—C24—C25	112.8 (3)	C2—C37—H37A	109.5
C31—C24—H24	106.4	C2—C37—H37B	109.5
C31—C24—C25	113.3 (3)	C2—C37—H37C	109.5
C25—C24—H24	106.4	H37A—C37—H37B	109.5
N4—C43—Au2	131.5 (3)	H37A—C37—H37C	109.5
N4—C43—N3	105.3 (3)	H37B—C37—H37C	109.5
N3—C43—Au2	123.1 (2)	C34—C35—C36	120.0 (4)
C5—C6—H6	119.3	C34—C35—H35	120.0
C7—C6—C5	121.5 (3)	C36—C35—H35	120.0
C7—C6—H6	119.3	C18—C19—H19	119.5
N3—C44—C79	123.7 (3)	C20—C19—C18	120.9 (3)
C45—C44—N3	106.3 (3)	C20—C19—H19	119.5
C45—C44—C79	129.9 (3)	N2—C39—C40	115.8 (3)
N4—C45—C80	123.9 (3)	N2—C39—H39A	108.3
C44—C45—N4	106.5 (3)	N2—C39—H39B	108.3
C44—C45—C80	129.6 (3)	C40—C39—H39A	108.3
C78—C73—C66	122.9 (3)	C40—C39—H39B	108.3
C78—C73—C74	118.1 (3)	H39A—C39—H39B	107.4
C74—C73—C66	119.0 (3)	C21—C20—H20	120.6
C60—C59—C52	123.9 (3)	C19—C20—C21	118.8 (4)
C60—C59—C64	119.0 (3)	C19—C20—H20	120.6
C64—C59—C52	117.1 (3)	C18—C17—C10	123.8 (3)
C32—C33—H33	119.8	C18—C17—C22	118.7 (3)
C34—C33—C32	120.4 (3)	C22—C17—C10	117.5 (3)
C34—C33—H33	119.8	C73—C74—H74	119.7
C62—C63—H63	120.1	C75—C74—C73	120.5 (4)
C64—C63—H63	120.1	C75—C74—H74	119.7
C64—C63—C62	119.7 (3)	C28—C29—C30	120.3 (4)
H41A—C41—H41B	107.9	C28—C29—H29	119.8
C42—C41—H41A	109.2	C30—C29—H29	119.8
C42—C41—H41B	109.2	C67—C72—H72	119.8
C40—C41—H41A	109.2	C67—C72—C71	120.4 (4)

C40—C41—H41B	109.2	C71—C72—H72	119.8
C40—C41—C42	112.2 (3)	C49—C65—H65A	109.5
C30—C25—C24	122.6 (3)	C49—C65—H65B	109.5
C30—C25—C26	118.7 (3)	C49—C65—H65C	109.5
C26—C25—C24	118.7 (3)	H65A—C65—H65B	109.5
C8—C7—C6	119.0 (3)	H65A—C65—H65C	109.5
C8—C7—C23	119.8 (3)	H65B—C65—H65C	109.5
C6—C7—C23	121.2 (3)	C28—C27—C26	120.1 (4)
C48—C49—C65	120.3 (3)	C28—C27—H27	120.0
C50—C49—C48	118.6 (3)	C26—C27—H27	120.0
C50—C49—C65	121.0 (3)	C12—C13—H13	119.9
C12—C11—C10	119.5 (3)	C12—C13—C14	120.2 (4)
C16—C11—C12	118.5 (3)	C14—C13—H13	119.9
C16—C11—C10	122.1 (3)	C55—C54—C53	120.4 (3)
C61—C62—C63	119.3 (3)	C55—C54—H54	119.8
C61—C62—H62	120.4	C53—C54—H54	119.8
C63—C62—H62	120.4	C57—C56—H56	120.4
C11—C12—H12	119.5	C55—C56—C57	119.3 (4)
C13—C12—C11	121.0 (4)	C55—C56—H56	120.4
C13—C12—H12	119.5	C78—C77—H77	120.0
C29—C28—H28	120.3	C76—C77—C78	120.0 (4)
C27—C28—H28	120.3	C76—C77—H77	120.0
C27—C28—C29	119.4 (3)	C16—C15—H15	119.8
C7—C23—H23A	109.5	C14—C15—C16	120.3 (4)
C7—C23—H23B	109.5	C14—C15—H15	119.8
C7—C23—H23C	109.5	C68—C69—H69	119.9
H23A—C23—H23B	109.5	C68—C69—C70	120.3 (4)
H23A—C23—H23C	109.5	C70—C69—H69	119.9
H23B—C23—H23C	109.5	C74—C75—H75	119.5
C47—C46—N3	119.1 (3)	C74—C75—C76	121.0 (4)
C51—C46—N3	118.8 (3)	C76—C75—H75	119.5
C51—C46—C47	122.1 (3)	C45—C80—H80A	109.5
C33—C34—H34	120.2	C45—C80—H80B	109.5
C33—C34—C35	119.6 (3)	C45—C80—H80C	109.5
C35—C34—H34	120.2	H80A—C80—H80B	109.5
C58—C57—H57	120.1	H80A—C80—H80C	109.5
C58—C57—C56	119.9 (4)	H80B—C80—H80C	109.5
C56—C57—H57	120.1	C72—C71—H71	119.7
C68—C67—C66	117.5 (3)	C70—C71—C72	120.7 (4)
C72—C67—C66	123.8 (3)	C70—C71—H71	119.7
C72—C67—C68	118.6 (4)	C69—C70—H70	120.2

C73—C78—H78	119.4	C71—C70—C69	119.5 (4)
C73—C78—C77	121.2 (3)	C71—C70—H70	120.2
C77—C78—H78	119.4	C77—C76—C75	119.1 (4)
C25—C30—H30	119.7	C77—C76—H76	120.4
C25—C30—C29	120.7 (4)	C75—C76—H76	120.4
C29—C30—H30	119.7	C13—C14—H14	120.1
C31—C36—H36	119.5	C15—C14—C13	119.7 (4)
C35—C36—C31	121.0 (3)	C15—C14—H14	120.1

N3—C44—C45—N4	0.1 (4)	C7—C8—C9—C24	175.6 (3)
N3—C44—C45—C80	-176.9 (3)	C11—C12—C13—C14	-0.7 (6)
N3—C46—C51—C66	9.5 (5)	C11—C16—C15—C14	-0.1 (6)
N3—C46—C51—C50	-175.0 (3)	C11—C10—C17—C18	27.2 (5)
N1—C2—C3—N2	0.1 (4)	C11—C10—C17—C22	-152.6 (3)
N1—C2—C3—C38	-173.7 (3)	C62—C61—C60—C59	0.9 (5)
C32—C31—C24—C9	29.0 (4)	C62—C63—C64—C59	0.6 (5)
C32—C31—C24—C25	-99.2 (4)	C12—C11—C16—C15	0.0 (5)
C32—C31—C36—C35	0.9 (6)	C12—C11—C10—C5	-134.7 (3)
C32—C33—C34—C35	0.3 (6)	C12—C11—C10—C17	96.3 (4)
C47—C52—C59—C60	-116.6 (3)	C12—C13—C14—C15	0.6 (6)
C47—C52—C59—C64	62.9 (4)	C46—N3—C43—Au2	-7.2 (4)
C47—C52—C53—C58	55.0 (4)	C46—N3—C43—N4	176.7 (3)
C47—C52—C53—C54	-122.9 (3)	C46—N3—C44—C79	6.2 (5)
C47—C46—C51—C66	-169.7 (3)	C46—N3—C44—C45	-177.0 (3)
C47—C46—C51—C50	5.9 (5)	C46—C47—C52—C59	-150.6 (3)
C8—C9—C4—N1	-173.9 (3)	C46—C47—C52—C53	79.0 (4)
C8—C9—C4—C5	3.3 (5)	C46—C47—C48—C49	1.3 (5)
C8—C9—C24—C31	-100.2 (4)	C46—C51—C66—C73	-89.0 (4)

C8—C9—C24—C25	28.3 (4)	C46—C51—C66—C67	142.1 (3)
C52—C47—C46—N3	-7.9 (5)	C46—C51—C50—C49	-1.3 (5)
C52—C47—C46—C51	171.2 (3)	C57—C58—C53—C52	179.4 (3)
C52—C47—C48—C49	-175.7 (3)	C57—C58—C53—C54	-2.6 (5)
C52—C59—C60—C61	180.0 (3)	C67—C68—C69—C70	-0.1 (6)
C52—C59—C64—C63	179.2 (3)	C67—C72—C71—C70	-1.3 (7)
C52—C53—C54—C55	179.2 (3)	C78—C73—C66—C67	85.7 (4)
C9—C8—C7—C6	-1.8 (5)	C78—C73—C66—C51	-41.0 (5)
C9—C8—C7—C23	179.3 (3)	C78—C73—C74—C75	-0.3 (5)
C9—C24—C25—C30	-101.5 (4)	C78—C77—C76—C75	-0.4 (6)
C9—C24—C25—C26	78.0 (4)	C30—C25—C26—C27	-1.1 (5)
C2—N1—C1—Au1	174.1 (2)	C36—C31—C24—C9	-150.7 (3)
C2—N1—C1—N2	-0.8 (4)	C36—C31—C24—C25	81.1 (4)
C2—N1—C4—C9	85.6 (4)	C60—C61—C62—C63	-1.5 (5)
C2—N1—C4—C5	-91.7 (4)	C60—C59—C64—C63	-1.2 (5)
C31—C32—C33—C34	-1.0 (5)	C16—C11—C12—C13	0.5 (5)
C31—C24—C25—C30	25.9 (5)	C16—C11—C10—C5	44.4 (4)
C31—C24—C25—C26	-154.7 (3)	C16—C11—C10—C17	-84.6 (4)
C31—C36—C35—C34	-1.6 (6)	C16—C15—C14—C13	-0.2 (6)
C3—N2—C1—Au1	-173.8 (2)	C10—C5—C4—N1	-4.5 (5)
C3—N2—C1—N1	0.9 (4)	C10—C5—C4—C9	178.2 (3)
C3—N2—C39—C40	-66.7 (4)	C10—C5—C6—C7	179.2 (3)
C1—N2—C3—C2	-0.7 (4)	C10—C11—C12—C13	179.6 (3)
C1—N2—C3—C38	173.6 (3)	C10—C11—C16—C15	-179.1 (3)
C1—N2—C39—C40	120.3 (4)	C48—C47—C52—C59	26.3 (4)
C1—N1—C2—C3	0.4 (4)	C48—C47—C52—C53	-104.0 (4)

C1—N1—C2—C37	-178.3 (3)	C48—C47—C46—N3	175.0 (3)
C1—N1—C4—C9	-103.4 (4)	C48—C47—C46—C51	-5.8 (5)
C1—N1—C4—C5	79.3 (4)	C48—C49—C50—C51	-3.0 (5)
C5—C6—C7—C8	1.6 (5)	C53—C52—C59—C60	12.1 (5)
C5—C6—C7—C23	-179.5 (3)	C53—C52—C59—C64	-168.3 (3)
C5—C10—C17—C18	-101.5 (4)	C53—C58—C57—C56	1.7 (5)
C5—C10—C17—C22	78.6 (4)	C66—C73—C78—C77	-180.0 (3)
C58—C57—C56—C55	0.7 (6)	C66—C73—C74—C75	-180.0 (3)
C58—C53—C54—C55	1.2 (5)	C66—C67—C68—C69	174.6 (3)
C79—C44—C45—N4	176.6 (3)	C66—C67—C72—C71	-173.7 (4)
C79—C44—C45—C80	-0.4 (6)	C66—C51—C50—C49	174.2 (3)
C4—N1—C2—C3	172.6 (3)	C68—C67—C66—C73	165.5 (3)
C4—N1—C2—C37	-6.1 (5)	C68—C67—C66—C51	-65.8 (4)
C4—N1—C1—Au1	2.2 (5)	C68—C67—C72—C71	3.0 (6)
C4—N1—C1—N2	-172.7 (3)	C68—C69—C70—C71	1.9 (7)
C4—C9—C24—C31	75.9 (4)	C50—C49—C48—C47	3.0 (5)
C4—C9—C24—C25	-155.6 (3)	C50—C51—C66—C73	95.6 (4)
C4—C5—C6—C7	0.9 (5)	C50—C51—C66—C67	-33.3 (4)
C4—C5—C10—C11	85.7 (4)	C26—C25—C30—C29	0.2 (6)
C4—C5—C10—C17	-144.8 (3)	C22—C21—C20—C19	0.7 (6)
C18—C19—C20—C21	-0.2 (5)	C64—C59—C60—C61	0.5 (5)
C24—C9—C4—N1	9.8 (5)	C64—C63—C62—C61	0.8 (5)
C24—C9—C4—C5	-173.0 (3)	C21—C22—C17—C18	-0.4 (5)
C24—C31—C36—C35	-179.5 (3)	C21—C22—C17—C10	179.4 (3)
C24—C25—C30—	179.7 (3)	C42—N4—C43—Au2	5.6 (5)

C29			
C24—C25—C26—C27	179.4 (3)	C42—N4—C43—N3	-178.7 (3)
C43—N4—C45—C44	-0.5 (4)	C42—N4—C45—C44	179.0 (3)
C43—N4—C45—C80	176.7 (3)	C42—N4—C45—C80	-3.8 (5)
C43—N4—C42—C41	94.7 (4)	C42—C41—C40—C39	-171.4 (3)
C43—N3—C44—C79	-176.4 (3)	C40—C41—C42—N4	-85.8 (4)
C43—N3—C44—C45	0.4 (4)	C37—C2—C3—N2	178.8 (3)
C43—N3—C46—C47	86.0 (4)	C37—C2—C3—C38	4.9 (6)
C43—N3—C46—C51	-93.2 (4)	C19—C18—C17—C10	-178.9 (3)
C6—C5—C4—N1	173.8 (3)	C19—C18—C17—C22	0.9 (5)
C6—C5—C4—C9	-3.5 (5)	C39—N2—C3—C2	-174.4 (3)
C6—C5—C10—C11	-92.5 (4)	C39—N2—C3—C38	-0.2 (5)
C6—C5—C10—C17	37.0 (4)	C39—N2—C1—Au1	0.0 (5)
C44—N3—C43—Au2	175.4 (2)	C39—N2—C1—N1	174.7 (3)
C44—N3—C43—N4	-0.7 (4)	C17—C18—C19—C20	-0.6 (5)
C44—N3—C46—C47	-97.0 (4)	C17—C22—C21—C20	-0.4 (6)
C44—N3—C46—C51	83.8 (4)	C74—C73—C78—C77	0.4 (5)
C45—N4—C43—Au2	-174.9 (3)	C74—C73—C66—C67	-94.7 (4)
C45—N4—C43—N3	0.7 (4)	C74—C73—C66—C51	138.6 (3)
C45—N4—C42—C41	-84.7 (4)	C74—C75—C76—C77	0.5 (6)
C73—C78—C77—C76	0.0 (6)	C29—C28—C27—C26	-0.7 (6)
C73—C74—C75—C76	-0.1 (6)	C72—C67—C66—C73	-17.8 (5)
C59—C52—C53—C58	-73.3 (4)	C72—C67—C66—C51	111.0 (4)
C59—C52—C53—C54	108.8 (3)	C72—C67—C68—C69	-2.3 (6)
C33—C32—C31—C24	-179.2 (3)	C72—C71—C70—C69	-1.2 (7)
C33—C32—C31—C36	0.4 (5)	C65—C49—C48—C47	179.2 (3)
C33—C34—C35—C36	1.0 (6)	C65—C49—C50—C51	-179.2 (3)
C41—C40—C39—N2	-62.3 (4)	C27—C28—C29—C30	-0.2 (6)
C25—C30—C29—	0.4 (6)	C54—C55—C56—	-2.1 (6)

C28		C57	
C25—C26—C27—C28	1.4 (6)	C56—C55—C54—C53	1.2 (5)
C7—C8—C9—C4	-0.6 (5)		

$Au_2Br_2$4e

Au1—Br1	2.3868 (3)	C24—H24	1.0000
Au1—C1	1.988 (3)	C40—C40^{i}	1.522 (5)
O1—C7	1.361 (3)	C40—H40A	0.9900
O1—C23	1.429 (4)	C40—H40B	0.9900
N1—C1	1.362 (3)	C26—H26	0.9500
N1—C2	1.394 (3)	C26—C27	1.390 (5)
N1—C4	1.433 (3)	C10—H10	1.0000
N2—C1	1.336 (4)	C10—C11	1.526 (4)
N2—C39	1.463 (3)	C34—H34	0.9500
N2—C3	1.398 (4)	C34—C35	1.390 (5)
C25—C30	1.392 (5)	C34—C33	1.382 (5)
C25—C24	1.522 (4)	C18—H18	0.9500
C25—C26	1.393 (5)	C18—C19	1.395 (5)
C31—C36	1.393 (4)	C19—H19	0.9500
C31—C32	1.387 (4)	C19—C20	1.373 (5)
C31—C24	1.532 (4)	C35—H35	0.9500
C37—H37A	0.9800	C16—H16	0.9500
C37—H37B	0.9800	C16—C11	1.398 (5)
C37—H37C	0.9800	C16—C15	1.396 (5)
C37—C2	1.492 (4)	C11—C12	1.389 (5)
C36—H36	0.9500	C38—H38A	0.9800
C36—C35	1.385 (5)	C38—H38B	0.9800
C6—H6	0.9500	C38—H38C	0.9800
C6—C7	1.396 (4)	C22—H22	0.9500
C6—C5	1.386 (4)	C22—C21	1.381 (5)
C2—C3	1.358 (4)	C33—H33	0.9500
C7—C8	1.394 (4)	C20—H20	0.9500
C32—H32	0.9500	C20—C21	1.390 (5)
C32—C33	1.398 (4)	C27—H27	0.9500
C39—H39A	0.9900	C27—C28	1.371 (7)
C39—H39B	0.9900	C12—H12	0.9500
C39—C40	1.519 (4)	C12—C13	1.398 (6)
C17—C10	1.525 (4)	C23—H23A	0.9800
C17—C18	1.394 (4)	C23—H23B	0.9800

C17—C22	1.392 (5)	C23—H23C	0.9800
C8—H8	0.9500	C28—H28	0.9500
C8—C9	1.388 (4)	C28—C29	1.377 (7)
C4—C9	1.394 (4)	C15—H15	0.9500
C4—C5	1.409 (4)	C15—C14	1.370 (7)
C3—C38	1.487 (4)	C14—H14	0.9500
C9—C24	1.534 (4)	C14—C13	1.390 (7)
C5—C10	1.523 (4)	C29—H29	0.9500
C30—H30	0.9500	C13—H13	0.9500
C30—C29	1.400 (6)	C21—H21	0.9500

C1—Au1—Br1	176.68 (7)	C40^{i}—C40—H40B	109.5
C7—O1—C23	118.1 (2)	H40A—C40—H40B	108.1
C1—N1—C2	110.5 (2)	C25—C26—H26	119.4
C1—N1—C4	125.1 (2)	C27—C26—C25	121.3 (3)
C2—N1—C4	124.4 (2)	C27—C26—H26	119.4
C1—N2—C39	123.0 (2)	C17—C10—H10	105.5
C1—N2—C3	111.4 (2)	C17—C10—C11	116.3 (2)
C3—N2—C39	125.3 (2)	C5—C10—C17	111.0 (2)
N1—C1—Au1	124.60 (19)	C5—C10—H10	105.5
N2—C1—Au1	130.0 (2)	C5—C10—C11	112.2 (2)
N2—C1—N1	105.3 (2)	C11—C10—H10	105.5
C30—C25—C24	119.3 (3)	C35—C34—H34	120.3
C30—C25—C26	117.9 (3)	C33—C34—H34	120.3
C26—C25—C24	122.8 (3)	C33—C34—C35	119.4 (3)
C36—C31—C24	117.2 (3)	C17—C18—H18	119.9
C32—C31—C36	119.2 (3)	C17—C18—C19	120.2 (3)
C32—C31—C24	123.5 (3)	C19—C18—H18	119.9
H37A—C37—H37B	109.5	C18—C19—H19	119.5
H37A—C37—H37C	109.5	C20—C19—C18	121.1 (3)
H37B—C37—H37C	109.5	C20—C19—H19	119.5
C2—C37—H37A	109.5	C36—C35—C34	120.6 (3)
C2—C37—H37B	109.5	C36—C35—H35	119.7
C2—C37—H37C	109.5	C34—C35—H35	119.7
C31—C36—H36	119.9	C11—C16—H16	120.1
C35—C36—C31	120.2 (3)	C15—C16—H16	120.1
C35—C36—H36	119.9	C15—C16—C11	119.7 (4)
C7—C6—H6	119.7	C16—C11—C10	121.5 (3)
C5—C6—H6	119.7	C12—C11—C10	119.5 (3)
C5—C6—C7	120.6 (2)	C12—C11—C16	118.9 (3)
N1—C2—C37	122.3 (2)	C3—C38—H38A	109.5

C3—C2—N1	106.6 (2)	C3—C38—H38B	109.5
C3—C2—C37	131.1 (3)	C3—C38—H38C	109.5
O1—C7—C6	116.8 (2)	H38A—C38—H38B	109.5
O1—C7—C8	123.0 (3)	H38A—C38—H38C	109.5
C8—C7—C6	120.2 (3)	H38B—C38—H38C	109.5
C31—C32—H32	119.8	C17—C22—H22	119.1
C31—C32—C33	120.3 (3)	C21—C22—C17	121.9 (3)
C33—C32—H32	119.8	C21—C22—H22	119.1
N2—C39—H39A	109.3	C32—C33—H33	119.9
N2—C39—H39B	109.3	C34—C33—C32	120.2 (3)
N2—C39—C40	111.6 (2)	C34—C33—H33	119.9
H39A—C39—H39B	108.0	C19—C20—H20	120.4
C40—C39—H39A	109.3	C19—C20—C21	119.3 (3)
C40—C39—H39B	109.3	C21—C20—H20	120.4
C18—C17—C10	124.8 (3)	C26—C27—H27	119.9
C22—C17—C10	117.4 (3)	C28—C27—C26	120.3 (4)
C22—C17—C18	117.8 (3)	C28—C27—H27	119.9
C7—C8—H8	120.0	C11—C12—H12	119.5
C9—C8—C7	120.0 (3)	C11—C12—C13	120.9 (4)
C9—C8—H8	120.0	C13—C12—H12	119.5
C9—C4—N1	119.6 (2)	O1—C23—H23A	109.5
C9—C4—C5	121.4 (2)	O1—C23—H23B	109.5
C5—C4—N1	119.0 (2)	O1—C23—H23C	109.5
N2—C3—C38	123.5 (3)	H23A—C23—H23B	109.5
C2—C3—N2	106.2 (2)	H23A—C23—H23C	109.5
C2—C3—C38	130.3 (3)	H23B—C23—H23C	109.5
C8—C9—C4	119.1 (2)	C27—C28—H28	120.2
C8—C9—C24	121.1 (2)	C27—C28—C29	119.6 (4)
C4—C9—C24	119.8 (2)	C29—C28—H28	120.2
C6—C5—C4	118.2 (3)	C16—C15—H15	119.5
C6—C5—C10	121.6 (2)	C14—C15—C16	121.0 (4)
C4—C5—C10	120.1 (2)	C14—C15—H15	119.5
C25—C30—H30	119.8	C15—C14—H14	120.0
C25—C30—C29	120.3 (4)	C15—C14—C13	120.0 (4)
C29—C30—H30	119.8	C13—C14—H14	120.0
C25—C24—C31	114.5 (2)	C30—C29—H29	119.7
C25—C24—C9	111.8 (2)	C28—C29—C30	120.6 (4)
C25—C24—H24	106.3	C28—C29—H29	119.7
C31—C24—C9	111.1 (2)	C12—C13—H13	120.3
C31—C24—H24	106.3	C14—C13—C12	119.4 (4)
C9—C24—H24	106.3	C14—C13—H13	120.3

C39—C40—C40^{i}	110.8 (3)	C22—C21—C20	119.7 (3)
C39—C40—H40A	109.5	C22—C21—H21	120.1
C39—C40—H40B	109.5	C20—C21—H21	120.1
C40^{i}—C40—H40A	109.5		

O1—C7—C8—C9	177.4 (3)	C4—N1—C2—C3	-177.1 (2)
N1—C2—C3—N2	-0.2 (3)	C4—C9—C24—C25	-91.7 (3)
N1—C2—C3—C38	179.2 (3)	C4—C9—C24—C31	139.0 (3)
N1—C4—C9—C8	-173.7 (2)	C4—C5—C10—C17	-135.3 (3)
N1—C4—C9—C24	6.6 (4)	C4—C5—C10—C11	92.8 (3)
N1—C4—C5—C6	173.3 (2)	C3—N2—C1—Au1	-178.3 (2)
N1—C4—C5—C10	-9.3 (4)	C3—N2—C1—N1	-0.4 (3)
N2—C39—C40—C40^{i}	-170.2 (3)	C3—N2—C39—C40	-75.7 (3)
C1—N1—C2—C37	-179.2 (3)	C9—C4—C5—C6	-6.2 (4)
C1—N1—C2—C3	0.0 (3)	C9—C4—C5—C10	171.3 (3)
C1—N1—C4—C9	-87.4 (3)	C5—C6—C7—O1	-177.8 (2)
C1—N1—C4—C5	93.1 (3)	C5—C6—C7—C8	5.0 (4)
C1—N2—C39—C40	98.5 (3)	C5—C4—C9—C8	5.7 (4)
C1—N2—C3—C2	0.4 (3)	C5—C4—C9—C24	-174.0 (2)
C1—N2—C3—C38	-179.1 (3)	C5—C10—C11—C16	57.1 (4)
C25—C30—C29—C28	0.6 (6)	C5—C10—C11—C12	-120.2 (3)
C25—C26—C27—C28	-0.3 (5)	C30—C25—C24—C31	-98.9 (3)
C31—C36—C35—C34	-1.2 (6)	C30—C25—C24—C9	133.6 (3)
C31—C32—C33—C34	-0.5 (5)	C30—C25—C26—C27	0.0 (5)
C37—C2—C3—N2	178.9 (3)	C24—C25—C30—C29	-178.3 (3)
C37—C2—C3—C38	-1.7 (5)	C24—C25—C26—C27	178.1 (3)
C36—C31—C32—C33	-0.3 (4)	C24—C31—C36—C35	-179.1 (3)
C36—C31—C24—C25	152.5 (3)	C24—C31—C32—C33	179.9 (3)
C36—C31—C24—C9	-79.7 (3)	C26—C25—C30—C29	-0.1 (5)
C6—C7—C8—C9	-5.5 (4)	C26—C25—C24—C31	82.9 (3)
C6—C5—C10—C17	42.1 (4)	C26—C25—C24—C9	-44.5 (4)
C6—C5—C10—C11	-89.8 (3)	C26—C27—C28—C29	0.8 (6)
C2—N1—C1—Au1	178.30 (19)	C10—C17—C18—C19	-178.4 (3)

C2—N1—C1—N2	0.3 (3)	C10—C17—C22—C21	178.0 (3)
C2—N1—C4—C9	89.2 (3)	C10—C11—C12—C13	176.5 (3)
C2—N1—C4—C5	-90.2 (3)	C18—C17—C10—C5	-118.0 (3)
C7—C6—C5—C4	0.8 (4)	C18—C17—C10—C11	11.8 (4)
C7—C6—C5—C10	-176.7 (3)	C18—C17—C22—C21	-1.4 (6)
C7—C8—C9—C4	0.2 (4)	C18—C19—C20—C21	0.8 (6)
C7—C8—C9—C24	179.9 (2)	C19—C20—C21—C22	-1.2 (6)
C32—C31—C36—C35	1.1 (5)	C35—C34—C33—C32	0.5 (5)
C32—C31—C24—C25	-27.8 (4)	C16—C11—C12—C13	-0.9 (5)
C32—C31—C24—C9	100.1 (3)	C16—C15—C14—C13	-0.5 (6)
C39—N2—C1—Au1	6.8 (4)	C11—C16—C15—C14	1.2 (5)
C39—N2—C1—N1	-175.3 (2)	C11—C12—C13—C14	1.5 (5)
C39—N2—C3—C2	175.2 (3)	C22—C17—C10—C5	62.7 (4)
C39—N2—C3—C38	-4.3 (4)	C22—C17—C10—C11	-167.6 (3)
C17—C10—C11—C16	-72.1 (4)	C22—C17—C18—C19	1.0 (5)
C17—C10—C11—C12	110.6 (3)	C33—C34—C35—C36	0.4 (6)
C17—C18—C19—C20	-0.7 (5)	C27—C28—C29—C30	-1.0 (6)
C17—C22—C21—C20	1.5 (6)	C23—O1—C7—C6	180.0 (3)
C8—C9—C24—C25	88.6 (3)	C23—O1—C7—C8	-2.9 (4)
C8—C9—C24—C31	-40.7 (4)	C15—C16—C11—C10	-177.8 (3)
C4—N1—C1—Au1	-4.7 (4)	C15—C16—C11—C12	-0.5 (5)
C4—N1—C1—N2	177.3 (2)	C15—C14—C13—C12	-0.8 (6)
C4—N1—C2—C37	3.7 (4)		

Au_2Br_24f

Au1—Br1	2.2902 (13)	C26—H26A	0.9800
Au1—C1	1.887 (11)	C26—H26B	0.9800

Au2—Br2	2.4455 (13)	C26—H26C	0.9800
Au2—C31	2.039 (11)	C27—H27A	0.9900
N1—C1	1.305 (13)	C27—H27B	0.9900
N1—C2	1.383 (14)	C27—C28	1.659 (17)
N1—C4	1.465 (14)	C28—H28A	0.9900
N2—C1	1.378 (15)	C28—H28B	0.9900
N2—C3	1.315 (14)	C28—C29	1.432 (16)
N2—C27	1.437 (14)	C29—H29A	0.9900
N3—C31	1.188 (14)	C29—H29B	0.9900
N3—C32	1.460 (15)	C29—C30	1.619 (16)
N3—C34	1.390 (13)	C30—H30	0.9500
N4—C30	1.385 (12)	C32—C33	1.294 (15)
N4—C31	1.289 (12)	C32—C55	1.441 (17)
N4—C33	1.403 (15)	C33—C56	1.405 (17)
C2—C3	1.353 (16)	C34—C35	1.417 (17)
C2—C25	1.406 (14)	C34—C39	1.446 (18)
C3—C26	1.473 (18)	C35—C36	1.308 (15)
C4—C5	1.527 (16)	C35—C40	1.618 (17)
C4—C9	1.445 (17)	C36—H36	0.9500
C5—C6	1.383 (16)	C36—C37	1.504 (18)
C5—C10	1.591 (15)	C37—C38	1.405 (17)
C6—H6	0.9500	C37—C53	1.423 (15)
C6—C7	1.413 (18)	C38—H38	0.9500
C7—C8	1.508 (19)	C38—C39	1.348 (17)
C7—C23	1.59 (2)	C39—C54	1.497 (17)
C8—H8	0.9500	C40—H40	1.0000
C8—C9	1.376 (19)	C40—C41	1.60 (2)
C9—C24	1.668 (18)	C40—C47	1.495 (19)
C10—H10	1.0000	C41—C42	1.429 (19)
C10—C11	1.379 (15)	C41—C46	1.331 (18)
C10—C17	1.636 (16)	C42—H42	0.9500
C11—C12	1.406 (18)	C42—C43	1.41 (2)
C11—C16	1.334 (13)	C43—H43	0.9500
C12—H12	0.9500	C43—C44	1.32 (2)
C12—C13	1.277 (18)	C44—H44	0.9500
C13—H13	0.9500	C44—C45	1.42 (2)
C13—C14	1.35 (2)	C45—H45	0.9500
C14—H14	0.9500	C45—C46	1.38 (2)
C14—C15	1.33 (2)	C46—H46	0.9500
C15—H15	0.9500	C47—C48	1.43 (2)
C15—C16	1.294 (17)	C47—C52	1.40 (2)

C16—H16	0.9500	C48—H48	0.9500
C17—C18	1.426 (16)	C48—C49	1.44 (2)
C17—C22	1.303 (16)	C49—H49	0.9500
C18—H18	0.9500	C49—C50	1.35 (2)
C18—C19	1.493 (17)	C50—H50	0.9500
C19—H19	0.9500	C50—C51	1.36 (2)
C19—C20	1.261 (18)	C51—H51	0.9500
C20—H20	0.9500	C51—C52	1.34 (2)
C20—C21	1.465 (19)	C52—H52	0.9500
C21—H21	0.9500	C53—H53A	0.9800
C21—C22	1.444 (18)	C53—H53B	0.9800
C22—H22	0.9500	C53—H53C	0.9800
C23—H23A	0.9800	C54—H54A	0.9800
C23—H23B	0.9800	C54—H54B	0.9800
C23—H23C	0.9800	C54—H54C	0.9800
C24—H24A	0.9800	C55—H55A	0.9800
C24—H24B	0.9800	C55—H55B	0.9800
C24—H24C	0.9800	C55—H55C	0.9800
C25—H25A	0.9800	C56—H56A	0.9800
C25—H25B	0.9800	C56—H56B	0.9800
C25—H25C	0.9800	C56—H56C	0.9800

C1—Au1—Br1	178.0 (3)	C28—C27—H27A	109.5
C31—Au2—Br2	176.5 (3)	C28—C27—H27B	109.5
C1—N1—C2	106.5 (9)	C27—C28—H28A	109.3
C1—N1—C4	125.7 (9)	C27—C28—H28B	109.3
C2—N1—C4	127.2 (9)	H28A—C28—H28B	108.0
C1—N2—C27	128.2 (9)	C29—C28—C27	111.6 (10)
C3—N2—C1	109.7 (9)	C29—C28—H28A	109.3
C3—N2—C27	122.1 (10)	C29—C28—H28B	109.3
C31—N3—C32	117.1 (9)	C28—C29—H29A	109.6
C31—N3—C34	124.0 (10)	C28—C29—H29B	109.6
C34—N3—C32	118.8 (9)	C28—C29—C30	110.3 (10)
C30—N4—C33	123.4 (9)	H29A—C29—H29B	108.1
C31—N4—C30	120.9 (10)	C30—C29—H29A	109.6
C31—N4—C33	115.7 (9)	C30—C29—H29B	109.6
N1—C1—Au1	121.9 (9)	N4—C30—C29	114.2 (9)
N1—C1—N2	108.4 (10)	N4—C30—H30	122.9
N2—C1—Au1	129.7 (8)	C29—C30—H30	122.9
N1—C2—C25	117.9 (10)	N3—C31—Au2	128.9 (8)
C3—C2—N1	109.3 (9)	N3—C31—N4	101.8 (10)

C3—C2—C25	132.7 (11)	N4—C31—Au2	129.2 (8)
N2—C3—C2	106.1 (10)	C33—C32—N3	102.0 (10)
N2—C3—C26	119.7 (11)	C33—C32—C55	130.0 (12)
C2—C3—C26	134.1 (11)	C55—C32—N3	128.0 (10)
N1—C4—C5	122.1 (10)	N4—C33—C56	129.3 (10)
C9—C4—N1	113.9 (10)	C32—C33—N4	103.4 (10)
C9—C4—C5	123.9 (10)	C32—C33—C56	127.0 (12)
C4—C5—C10	122.4 (10)	N3—C34—C35	108.8 (10)
C6—C5—C4	118.3 (10)	N3—C34—C39	121.4 (10)
C6—C5—C10	119.2 (10)	C35—C34—C39	129.7 (10)
C5—C6—H6	120.7	C34—C35—C40	127.9 (10)
C5—C6—C7	118.5 (11)	C36—C35—C34	110.6 (11)
C7—C6—H6	120.7	C36—C35—C40	121.5 (11)
C6—C7—C8	122.3 (12)	C35—C36—H36	118.9
C6—C7—C23	115.2 (12)	C35—C36—C37	122.3 (11)
C8—C7—C23	122.2 (13)	C37—C36—H36	118.9
C7—C8—H8	119.0	C38—C37—C36	124.8 (11)
C9—C8—C7	121.9 (12)	C38—C37—C53	116.1 (12)
C9—C8—H8	119.0	C53—C37—C36	119.0 (11)
C4—C9—C24	123.5 (11)	C37—C38—H38	123.2
C8—C9—C4	115.0 (11)	C39—C38—C37	113.6 (12)
C8—C9—C24	121.5 (11)	C39—C38—H38	123.2
C5—C10—H10	106.3	C34—C39—C54	130.7 (11)
C5—C10—C17	115.5 (9)	C38—C39—C34	119.0 (11)
C11—C10—C5	110.1 (9)	C38—C39—C54	110.3 (11)
C11—C10—H10	106.3	C35—C40—H40	105.5
C11—C10—C17	111.8 (9)	C41—C40—C35	113.7 (10)
C17—C10—H10	106.3	C41—C40—H40	105.5
C10—C11—C12	120.7 (11)	C47—C40—C35	114.7 (10)
C16—C11—C10	117.2 (11)	C47—C40—H40	105.5
C16—C11—C12	122.1 (11)	C47—C40—C41	110.9 (11)
C11—C12—H12	119.4	C42—C41—C40	124.4 (11)
C13—C12—C11	121.2 (14)	C46—C41—C40	123.6 (12)
C13—C12—H12	119.4	C46—C41—C42	111.8 (14)
C12—C13—H13	122.4	C41—C42—H42	116.9
C12—C13—C14	115.2 (15)	C43—C42—C41	126.3 (13)
C14—C13—H13	122.4	C43—C42—H42	116.9
C13—C14—H14	118.1	C42—C43—H43	120.5
C15—C14—C13	123.8 (13)	C44—C43—C42	118.9 (14)
C15—C14—H14	118.1	C44—C43—H43	120.5
C14—C15—H15	118.7	C43—C44—H44	122.1

C16—C15—C14	122.6 (15)	C43—C44—C45	115.9 (17)
C16—C15—H15	118.7	C45—C44—H44	122.1
C11—C16—H16	122.4	C44—C45—H45	118.0
C15—C16—C11	115.1 (14)	C46—C45—C44	123.9 (15)
C15—C16—H16	122.4	C46—C45—H45	118.0
C18—C17—C10	129.0 (10)	C41—C46—C45	123.0 (14)
C22—C17—C10	117.7 (10)	C41—C46—H46	118.5
C22—C17—C18	113.3 (11)	C45—C46—H46	118.5
C17—C18—H18	116.8	C48—C47—C40	117.3 (14)
C17—C18—C19	126.3 (11)	C52—C47—C40	118.4 (12)
C19—C18—H18	116.8	C52—C47—C48	124.2 (15)
C18—C19—H19	119.8	C47—C48—H48	127.2
C20—C19—C18	120.3 (12)	C47—C48—C49	105.6 (19)
C20—C19—H19	119.8	C49—C48—H48	127.2
C19—C20—H20	123.5	C48—C49—H49	114.6
C19—C20—C21	113.1 (12)	C50—C49—C48	130.9 (17)
C21—C20—H20	123.5	C50—C49—H49	114.6
C20—C21—H21	116.5	C49—C50—H50	120.7
C22—C21—C20	126.9 (11)	C49—C50—C51	118.6 (16)
C22—C21—H21	116.5	C51—C50—H50	120.7
C17—C22—C21	119.9 (11)	C50—C51—H51	121.6
C17—C22—H22	120.0	C52—C51—C50	116.8 (18)
C21—C22—H22	120.0	C52—C51—H51	121.6
C7—C23—H23A	109.5	C47—C52—H52	118.1
C7—C23—H23B	109.5	C51—C52—C47	123.7 (15)
C7—C23—H23C	109.5	C51—C52—H52	118.1
H23A—C23—H23B	109.5	C37—C53—H53A	109.5
H23A—C23—H23C	109.5	C37—C53—H53B	109.5
H23B—C23—H23C	109.5	C37—C53—H53C	109.5
C9—C24—H24A	109.5	H53A—C53—H53B	109.5
C9—C24—H24B	109.5	H53A—C53—H53C	109.5
C9—C24—H24C	109.5	H53B—C53—H53C	109.5
H24A—C24—H24B	109.5	C39—C54—H54A	109.5
H24A—C24—H24C	109.5	C39—C54—H54B	109.5
H24B—C24—H24C	109.5	C39—C54—H54C	109.5
C2—C25—H25A	109.5	H54A—C54—H54B	109.5
C2—C25—H25B	109.5	H54A—C54—H54C	109.5
C2—C25—H25C	109.5	H54B—C54—H54C	109.5
H25A—C25—H25B	109.5	C32—C55—H55A	109.5
H25A—C25—H25C	109.5	C32—C55—H55B	109.5
H25B—C25—H25C	109.5	C32—C55—H55C	109.5

C3—C26—H26A	109.5	H55A—C55—H55B	109.5
C3—C26—H26B	109.5	H55A—C55—H55C	109.5
C3—C26—H26C	109.5	H55B—C55—H55C	109.5
H26A—C26—H26B	109.5	C33—C56—H56A	109.5
H26A—C26—H26C	109.5	C33—C56—H56B	109.5
H26B—C26—H26C	109.5	C33—C56—H56C	109.5
N2—C27—H27A	109.5	H56A—C56—H56B	109.5
N2—C27—H27B	109.5	H56A—C56—H56C	109.5
N2—C27—C28	110.7 (9)	H56B—C56—H56C	109.5
H27A—C27—H27B	108.1		

N1—C2—C3—N2	-0.3 (13)	C25—C2—C3—C26	-2 (2)
N1—C2—C3—C26	-178.9 (13)	C27—N2—C1—Au1	5.2 (17)
N1—C4—C5—C6	179.3 (9)	C27—N2—C1—N1	-178.0 (10)
N1—C4—C5—C10	3.1 (15)	C27—N2—C3—C2	178.1 (10)
N1—C4—C9—C8	179.7 (10)	C27—N2—C3—C26	-3.0 (17)
N1—C4—C9—C24	0.6 (15)	C27—C28—C29—C30	171.2 (9)
N2—C27—C28—C29	-73.8 (12)	C28—C29—C30—N4	171.9 (10)
N3—C32—C33—N4	-0.7 (12)	C30—N4—C31—Au2	-6.3 (16)
N3—C32—C33—C56	-174.7 (13)	C30—N4—C31—N3	177.3 (10)
N3—C34—C35—C36	179.9 (11)	C30—N4—C33—C32	-178.2 (11)
N3—C34—C35—C40	1.1 (18)	C30—N4—C33—C56	-4 (2)
N3—C34—C39—C38	179.4 (12)	C31—N3—C32—C33	-1.6 (14)
N3—C34—C39—C54	-1 (2)	C31—N3—C32—C55	177.1 (13)
C1—N1—C2—C3	-0.1 (13)	C31—N3—C34—C35	90.4 (14)
C1—N1—C2—C25	-177.6 (11)	C31—N3—C34—C39	-87.8 (16)
C1—N1—C4—C5	79.8 (14)	C31—N4—C30—C29	-115.1 (12)
C1—N1—C4—C9	-98.8 (13)	C31—N4—C33—C32	2.7 (14)
C1—N2—C3—C2	0.5 (13)	C31—N4—C33—C56	176.6 (13)
C1—N2—C3—C26	179.4 (10)	C32—N3—C31—Au2	-173.3 (8)
C1—N2—C27—C28	102.2 (13)	C32—N3—C31—N4	3.1 (13)
C2—N1—C1—Au1	177.5 (8)	C32—N3—C34—C35	-94.5 (13)
C2—N1—C1—N2	0.4 (12)	C32—N3—C34—C39	87.3 (14)
C2—N1—C4—C5	-110.7 (12)	C33—N4—C30—C29	65.9 (14)
C2—N1—C4—C9	70.7 (14)	C33—N4—C31—Au2	172.8 (8)
C3—N2—C1—Au1	-177.4 (9)	C33—N4—C31—N3	-3.6 (13)
C3—N2—C1—N1	-0.6 (13)	C34—N3—C31—Au2	1.9 (17)
C3—N2—C27—C28	-74.9 (13)	C34—N3—C31—N4	178.3 (11)
C4—N1—C1—Au1	-11.2 (15)	C34—N3—C32—C33	-177.1 (11)
C4—N1—C1—N2	171.7 (10)	C34—N3—C32—C55	1.6 (18)

C4—N1—C2—C3	-171.2 (10)	C34—C35—C36—C37	1.4 (19)
C4—N1—C2—C25	11.3 (17)	C34—C35—C40—C41	86.1 (14)
C4—C5—C6—C7	2.4 (16)	C34—C35—C40—C47	-144.8 (13)
C4—C5—C10—C11	74.7 (13)	C35—C34—C39—C38	2 (2)
C4—C5—C10—C17	-157.6 (9)	C35—C34—C39—C54	-178.9 (14)
C5—C4—C9—C8	1.2 (16)	C35—C36—C37—C38	-1 (2)
C5—C4—C9—C24	-177.9 (10)	C35—C36—C37—C53	-176.9 (14)
C5—C6—C7—C8	-1.9 (17)	C35—C40—C41—C42	-135.6 (12)
C5—C6—C7—C23	-175.6 (13)	C35—C40—C41—C46	38.7 (16)
C5—C10—C11—C12	-125.4 (10)	C35—C40—C47—C48	-101.8 (14)
C5—C10—C11—C16	53.2 (14)	C35—C40—C47—C52	74.7 (14)
C5—C10—C17—C18	-129.6 (12)	C36—C35—C40—C41	-92.6 (15)
C5—C10—C17—C22	50.2 (14)	C36—C35—C40—C47	36.5 (18)
C6—C5—C10—C11	-101.5 (12)	C36—C37—C38—C39	0 (2)
C6—C5—C10—C17	26.2 (14)	C37—C38—C39—C34	-0.4 (18)
C6—C7—C8—C9	0.8 (18)	C37—C38—C39—C54	-179.9 (12)
C7—C8—C9—C4	-0.5 (17)	C39—C34—C35—C36	-2 (2)
C7—C8—C9—C24	178.6 (11)	C39—C34—C35—C40	179.1 (12)
C9—C4—C5—C6	-2.2 (16)	C40—C35—C36—C37	-179.7 (12)
C9—C4—C5—C10	-178.5 (10)	C40—C41—C42—C43	179.1 (14)
C10—C5—C6—C7	178.8 (10)	C40—C41—C46—C45	-176.6 (13)
C10—C11—C12—C13	179.3 (12)	C40—C47—C48—C49	174.9 (11)
C10—C11—C16—C15	-177.5 (12)	C40—C47—C52—C51	-173.0 (13)
C10—C17—C18—C19	-179.3 (11)	C41—C40—C47—C48	28.6 (14)

C10—C17—C22—C21	-178.9 (10)	C41—C40—C47—C52	-154.8 (11)
C11—C10—C17—C18	-2.6 (16)	C41—C42—C43—C44	-5 (3)
C11—C10—C17—C22	177.2 (11)	C42—C41—C46—C45	-2 (2)
C11—C12—C13—C14	-1.9 (14)	C42—C43—C44—C45	2 (3)
C12—C11—C16—C15	1.0 (17)	C43—C44—C45—C46	0 (3)
C12—C13—C14—C15	1 (2)	C44—C45—C46—C41	0 (3)
C13—C14—C15—C16	1 (2)	C46—C41—C42—C43	4 (2)
C14—C15—C16—C11	-2 (2)	C47—C40—C41—C42	93.4 (14)
C16—C11—C12—C13	0.8 (14)	C47—C40—C41—C46	-92.3 (14)
C17—C10—C11—C12	104.9 (11)	C47—C48—C49—C50	0 (2)
C17—C10—C11—C16	-76.6 (13)	C48—C47—C52—C51	3 (2)
C17—C18—C19—C20	-1 (2)	C48—C49—C50—C51	0 (3)
C18—C17—C22—C21	0.9 (17)	C49—C50—C51—C52	2 (2)
C18—C19—C20—C21	-0.2 (18)	C50—C51—C52—C47	-3 (2)
C19—C20—C21—C22	2 (2)	C52—C47—C48—C49	-1.4 (18)
C20—C21—C22—C17	-3 (2)	C53—C37—C38—C39	176.3 (13)
C22—C17—C18—C19	0.9 (17)	C55—C32—C33—N4	-179.4 (13)
C23—C7—C8—C9	174.1 (14)	C55—C32—C33—C56	7 (2)
C25—C2—C3—N2	176.7 (13)		

$Au_2Br_2$4g

Au1—Br1	2.3917 (3)	C27—C28	1.385 (4)
Au1—C1	1.991 (2)	C8—H8	0.9500
N2—C1	1.342 (3)	C8—C9	1.397 (3)
N2—C3	1.393 (3)	C39—H39A	0.9900
N2—C39	1.476 (3)	C39—H39B	0.9900
C1—N1	1.357 (3)	C39—C40	1.534 (4)
C3—C38	1.494 (3)	C31—C36	1.392 (3)
C3—C2	1.362 (3)	C12—H12	0.9500

C6—H6	0.9500	C12—C13	1.386 (4)
C6—C7	1.389 (3)	C18—H18	0.9500
C6—C5	1.394 (3)	C18—C19	1.395 (3)
C25—C26	1.396 (3)	C23—H23A	0.9800
C25—C24	1.523 (3)	C23—H23B	0.9800
C25—C30	1.392 (3)	C23—H23C	0.9800
C26—H26	0.9500	C14—H14	0.9500
C26—C27	1.388 (3)	C14—C15	1.386 (4)
C24—H24	1.0000	C14—C13	1.391 (4)
C24—C31	1.524 (3)	C29—H29	0.9500
C24—C9	1.529 (3)	C29—C28	1.382 (4)
C38—H38A	0.9800	C29—C30	1.389 (3)
C38—H38B	0.9800	C4—C9	1.400 (3)
C38—H38C	0.9800	C36—H36	0.9500
C10—H10	1.0000	C36—C35	1.390 (4)
C10—C17	1.528 (3)	C42—H42A	0.9800
C10—C11	1.524 (3)	C42—H42B	0.9800
C10—C5	1.534 (3)	C42—H42C	0.9800
C22—H22	0.9500	C40—H40A	0.9900
C22—C17	1.403 (3)	C40—H40B	0.9900
C22—C21	1.380 (4)	C33—H33	0.9500
C7—C8	1.389 (3)	C33—C34	1.376 (4)
C7—C23	1.514 (3)	C15—H15	0.9500
N1—C4	1.443 (3)	C15—C16	1.390 (3)
N1—C2	1.395 (3)	C28—H28	0.9500
C17—C18	1.383 (3)	C30—H30	0.9500
C21—H21	0.9500	C2—C37	1.490 (3)
C21—C20	1.392 (4)	C20—H20	0.9500
C41—H41A	0.9900	C20—C19	1.386 (4)
C41—H41B	0.9900	C19—H19	0.9500
C41—C42	1.513 (4)	C13—H13	0.9500
C41—C40	1.518 (4)	C16—H16	0.9500
C11—C12	1.397 (3)	C37—H37A	0.9800
C11—C16	1.394 (3)	C37—H37B	0.9800
C32—H32	0.9500	C37—H37C	0.9800
C32—C31	1.394 (3)	C35—H35	0.9500
C32—C33	1.399 (4)	C35—C34	1.384 (4)
C5—C4	1.398 (3)	C34—H34	0.9500
C27—H27	0.9500		

C1—Au1—Br1	175.10 (6)	C36—C31—C24	118.0 (2)

C1—N2—C3	110.88 (19)	C36—C31—C32	118.8 (2)
C1—N2—C39	123.8 (2)	C11—C12—H12	119.5
C3—N2—C39	125.2 (2)	C13—C12—C11	121.0 (2)
N2—C1—Au1	130.37 (17)	C13—C12—H12	119.5
N2—C1—N1	105.37 (19)	C17—C18—H18	119.5
N1—C1—Au1	123.94 (16)	C17—C18—C19	120.9 (2)
N2—C3—C38	123.5 (2)	C19—C18—H18	119.5
C2—C3—N2	106.9 (2)	C7—C23—H23A	109.5
C2—C3—C38	129.6 (2)	C7—C23—H23B	109.5
C7—C6—H6	118.9	C7—C23—H23C	109.5
C7—C6—C5	122.1 (2)	H23A—C23—H23B	109.5
C5—C6—H6	118.9	H23A—C23—H23C	109.5
C26—C25—C24	119.6 (2)	H23B—C23—H23C	109.5
C30—C25—C26	118.5 (2)	C15—C14—H14	120.5
C30—C25—C24	121.8 (2)	C15—C14—C13	119.1 (2)
C25—C26—H26	119.6	C13—C14—H14	120.5
C27—C26—C25	120.8 (2)	C28—C29—H29	119.7
C27—C26—H26	119.6	C28—C29—C30	120.6 (2)
C25—C24—H24	106.4	C30—C29—H29	119.7
C25—C24—C31	114.54 (19)	C5—C4—N1	120.0 (2)
C25—C24—C9	111.62 (18)	C5—C4—C9	121.9 (2)
C31—C24—H24	106.4	C9—C4—N1	118.06 (19)
C31—C24—C9	111.02 (18)	C31—C36—H36	119.7
C9—C24—H24	106.4	C35—C36—C31	120.6 (3)
C3—C38—H38A	109.5	C35—C36—H36	119.7
C3—C38—H38B	109.5	C41—C42—H42A	109.5
C3—C38—H38C	109.5	C41—C42—H42B	109.5
H38A—C38—H38B	109.5	C41—C42—H42C	109.5
H38A—C38—H38C	109.5	H42A—C42—H42B	109.5
H38B—C38—H38C	109.5	H42A—C42—H42C	109.5
C17—C10—H10	106.8	H42B—C42—H42C	109.5
C17—C10—C5	110.50 (19)	C8—C9—C24	121.0 (2)
C11—C10—H10	106.8	C8—C9—C4	117.9 (2)
C11—C10—C17	113.76 (19)	C4—C9—C24	121.19 (19)
C11—C10—C5	111.63 (19)	C41—C40—C39	115.4 (2)
C5—C10—H10	106.8	C41—C40—H40A	108.4
C17—C22—H22	119.6	C41—C40—H40B	108.4
C21—C22—H22	119.6	C39—C40—H40A	108.4
C21—C22—C17	120.8 (2)	C39—C40—H40B	108.4
C6—C7—C23	120.9 (2)	H40A—C40—H40B	107.5
C8—C7—C6	118.5 (2)	C32—C33—H33	119.6

C8—C7—C23	120.6 (2)	C34—C33—C32	120.7 (3)
C1—N1—C4	122.33 (18)	C34—C33—H33	119.6
C1—N1—C2	111.02 (19)	C14—C15—H15	119.8
C2—N1—C4	125.89 (19)	C14—C15—C16	120.4 (2)
C22—C17—C10	118.5 (2)	C16—C15—H15	119.8
C18—C17—C10	123.1 (2)	C27—C28—H28	120.3
C18—C17—C22	118.3 (2)	C29—C28—C27	119.4 (2)
C22—C21—H21	119.8	C29—C28—H28	120.3
C22—C21—C20	120.5 (2)	C25—C30—H30	119.8
C20—C21—H21	119.8	C29—C30—C25	120.5 (2)
H41A—C41—H41B	107.6	C29—C30—H30	119.8
C42—C41—H41A	108.7	C3—C2—N1	105.8 (2)
C42—C41—H41B	108.7	C3—C2—C37	130.9 (2)
C42—C41—C40	114.2 (3)	N1—C2—C37	123.2 (2)
C40—C41—H41A	108.7	C21—C20—H20	120.5
C40—C41—H41B	108.7	C19—C20—C21	119.1 (2)
C12—C11—C10	120.2 (2)	C19—C20—H20	120.5
C16—C11—C10	121.8 (2)	C18—C19—H19	119.8
C16—C11—C12	118.0 (2)	C20—C19—C18	120.3 (2)
C31—C32—H32	120.0	C20—C19—H19	119.8
C31—C32—C33	120.0 (2)	C12—C13—C14	120.5 (2)
C33—C32—H32	120.0	C12—C13—H13	119.8
C6—C5—C10	120.8 (2)	C14—C13—H13	119.8
C6—C5—C4	117.7 (2)	C11—C16—H16	119.5
C4—C5—C10	121.5 (2)	C15—C16—C11	121.0 (2)
C26—C27—H27	119.9	C15—C16—H16	119.5
C28—C27—C26	120.2 (2)	C2—C37—H37A	109.5
C28—C27—H27	119.9	C2—C37—H37B	109.5
C7—C8—H8	119.1	C2—C37—H37C	109.5
C7—C8—C9	121.8 (2)	H37A—C37—H37B	109.5
C9—C8—H8	119.1	H37A—C37—H37C	109.5
N2—C39—H39A	109.2	H37B—C37—H37C	109.5
N2—C39—H39B	109.2	C36—C35—H35	119.8
N2—C39—C40	112.3 (2)	C34—C35—C36	120.5 (3)
H39A—C39—H39B	107.9	C34—C35—H35	119.8
C40—C39—H39A	109.2	C33—C34—C35	119.4 (2)
C40—C39—H39B	109.2	C33—C34—H34	120.3
C32—C31—C24	123.2 (2)	C35—C34—H34	120.3

Au1—C1—N1—C4	-15.8 (3)	C17—C10—C5—C4	-147.0 (2)
Au1—C1—N1—C2	173.68 (16)	C17—C22—C21—	0.4 (4)

		C20	
N2—C1—N1—C4	170.17 (19)	C17—C18—C19—C20	0.7 (4)
N2—C1—N1—C2	-0.4 (2)	C21—C22—C17—C10	-176.7 (2)
N2—C3—C2—N1	-0.1 (3)	C21—C22—C17—C18	1.2 (4)
N2—C3—C2—C37	178.0 (2)	C21—C20—C19—C18	0.9 (4)
N2—C39—C40—C41	-63.3 (3)	C11—C10—C17—C22	-171.9 (2)
C1—N2—C3—C38	-179.4 (2)	C11—C10—C17—C18	10.3 (3)
C1—N2—C3—C2	-0.1 (3)	C11—C10—C5—C6	-96.5 (3)
C1—N2—C39—C40	104.7 (3)	C11—C10—C5—C4	85.4 (3)
C1—N1—C4—C5	92.1 (3)	C11—C12—C13—C14	0.4 (4)
C1—N1—C4—C9	-85.4 (3)	C32—C31—C36—C35	-0.8 (4)
C1—N1—C2—C3	0.3 (3)	C32—C33—C34—C35	-0.3 (4)
C1—N1—C2—C37	-178.0 (2)	C5—C6—C7—C8	2.7 (4)
C3—N2—C1—Au1	-173.23 (17)	C5—C6—C7—C23	-178.7 (2)
C3—N2—C1—N1	0.3 (3)	C5—C10—C17—C22	61.6 (3)
C3—N2—C39—C40	-72.9 (3)	C5—C10—C17—C18	-116.2 (2)
C6—C7—C8—C9	-2.0 (3)	C5—C10—C11—C12	-131.5 (2)
C6—C5—C4—N1	179.0 (2)	C5—C10—C11—C16	49.5 (3)
C6—C5—C4—C9	-3.6 (3)	C5—C4—C9—C24	-175.4 (2)
C25—C26—C27—C28	-0.3 (4)	C5—C4—C9—C8	4.2 (3)
C25—C24—C31—C32	-29.3 (3)	C39—N2—C1—Au1	8.8 (3)
C25—C24—C31—C36	153.4 (2)	C39—N2—C1—N1	-177.6 (2)
C25—C24—C9—C8	93.6 (2)	C39—N2—C3—C38	-1.5 (4)
C25—C24—C9—C4	-86.8 (2)	C39—N2—C3—C2	177.8 (2)
C26—C25—C24—C31	-95.0 (3)	C31—C24—C9—C8	-35.5 (3)
C26—C25—C24—C9	137.7 (2)	C31—C24—C9—C4	144.1 (2)
C26—C25—C30—C29	-0.6 (4)	C31—C32—C33—C34	-0.6 (4)
C26—C27—C28—C29	0.7 (4)	C31—C36—C35—C34	-0.1 (4)
C24—C25—C26—C27	-177.2 (2)	C12—C11—C16—C15	0.0 (3)
C24—C25—C30—	176.7 (2)	C23—C7—C8—C9	179.4 (2)

C29			
C24—C31—C36—C35	176.7 (2)	C14—C15—C16—C11	0.2 (4)
C38—C3—C2—N1	179.1 (2)	C4—N1—C2—C3	-169.8 (2)
C38—C3—C2—C37	-2.7 (4)	C4—N1—C2—C37	11.9 (3)
C10—C17—C18—C19	176.1 (2)	C36—C35—C34—C33	0.7 (4)
C10—C11—C12—C13	-179.4 (2)	C42—C41—C40—C39	-71.4 (3)
C10—C11—C16—C15	179.1 (2)	C9—C24—C31—C32	98.3 (3)
C10—C5—C4—N1	-2.8 (3)	C9—C24—C31—C36	-79.0 (3)
C10—C5—C4—C9	174.6 (2)	C33—C32—C31—C24	-176.1 (2)
C22—C17—C18—C19	-1.7 (4)	C33—C32—C31—C36	1.1 (4)
C22—C21—C20—C19	-1.4 (4)	C15—C14—C13—C12	-0.1 (4)
C7—C6—C5—C10	-178.1 (2)	C28—C29—C30—C25	1.1 (4)
C7—C6—C5—C4	0.1 (4)	C30—C25—C26—C27	0.2 (4)
C7—C8—C9—C24	178.3 (2)	C30—C25—C24—C31	87.7 (3)
C7—C8—C9—C4	-1.3 (3)	C30—C25—C24—C9	-39.6 (3)
N1—C4—C9—C24	2.0 (3)	C30—C29—C28—C27	-1.1 (4)
N1—C4—C9—C8	-178.35 (19)	C2—N1—C4—C5	-98.8 (3)
C17—C10—C11—C12	102.6 (2)	C2—N1—C4—C9	83.6 (3)
C17—C10—C11—C16	-76.4 (3)	C13—C14—C15—C16	-0.1 (4)
C17—C10—C5—C6	31.2 (3)	C16—C11—C12—C13	-0.3 (4)

Symmetry code(s): (i) -*x*+2, -*y*+1, -*z*+1.

Additional Studies Referred to from the Main Manuscript

Additional Discussion Regarding %V_{bur}. The geometry of binuclear complexes **4a–4f** was analyzed by the buried volume method pioneered by Nolan and Cavallo (Table S3 and S4). The analysis revealed a remarkable range of steric environment around the metal center (%V_{bur} of 33.9 up to 60.4%). As such, [Au(IPr*$^{diNHC–C3}$)Br] (**4a**) is characterized by the % buried volume (%V_{bur}) of 60.4% (Au2) with quadrant distribution of SW, 62.4%; NW, 62.0%; NE, 60.7%; SE, 56.6%, and the (%V_{bur}) of 50.8% (Au1) with quadrant distribution of SW, 49.7%; NW, 26.3%; NE, 56.5%; SE, 70.5%. This can be compared with the least sterically-demanding [Au(IPaul*$^{diNHC–C4}$)Br] (**4f**), which is characterized by the (%V_{bur}) of 33.9% (Au1) with quadrant distribution of SW, 27.5%; NW, 25.1%; NE, 36.2%; SE, 46.7%, and the (%V_{bur}) of 39.9% (Au2) with quadrant distribution of SW, 25.9%; NW, 42.6%; NE, 38.1%; SE, 53.0%.

The remaining dinuclear complexes are in the range between 36.6% (**4b**) and 52.2% (**4c**) and are characterized as follows in the C3 series: [Au(IPr*$^{MeOdiNHC–C3}$)Br] (**4b**) (%V_{bur}) of 36.6% (Au1) with quadrant distribution of SW, 29.1%; NW, 25.4%; NE, 52.2%; SE, 39.8%, and the (%V_{bur}) of 42.0% (Au2) with quadrant distribution of SW, 48.9%; NW, 26.9%; NE, 40.6%; SE, 51.7%, and [Au(IPaul*$^{diNHC–C3}$)Br] (**4c**) (%V_{bur}) of 35.7% (Au1) with quadrant distribution of SW, 25.2%; NW, 36.1%; NE, 34.3%; SE, 47.2%, and the (%V_{bur}) of 52.2% (Au2) with quadrant distribution of SW, 55.5%; NW, 49.7%; NE, 66.1%; SE, 37.5%. Furthermore, the remaining complexes in the C4 series are characterized as follows: [Au(IPr*$^{diNHC–C4}$)Br] (**4d**) (%V_{bur}) of 48.6% (Au1) with quadrant distribution of SW, 26.5%; NW, 39.9%; NE, 70.4%; SE, 57.7%, and the (%V_{bur}) of 41.4% (Au2) with quadrant distribution of SW, 25.3%; NW, 41.1%; NE, 44.7%; SE, 54.3%, and [Au(IPr*$^{MeOdiNHC–C4}$)Br] (**4e**) (%V_{bur}) of 40.2% (Au1) with quadrant distribution of SW, 25.6%; NW, 29.8%; NE, 52.3%; SE, 53.0%, and the (%V_{bur}) of 40.2% (Au2) with quadrant distribution of SW, 29.8%; NW, 25.6%; NE, 53.0%; SE, 52.3%. These values can be compared with the (%V_{bur}) of the reference mononuclear [Au(IPr*$^{n\text{-}Bu}$)Br] (**4g**) of 42.8% with quadrant distribution of SW, 25.2%; NW, 46.8%; NE, 46.6%; SE, 52.3% as well as with the symmetrical mononuclear imidazol-2-ylidene, IPr*, [Au(IPr*)Cl], (%V_{bur}) = 50.4% with quadrant distribution of SW, 60.2%; NW, 52.8%; NE, 41.7%; SE, 47.0%, and IPr, [Au(IPr)Cl], (%V_{bur}) = 45.7% with quadrant distribution of SW, 49.4%; NW, 42.3%; NE, 50.3%; SE, 40.6%. Thus, it is evident that both the N-aromatic wingtip and the bridging group in the dinuclear complexes **4a–4f** impact the steric environment with the key feature as the rotatable substitution.

Table S3. Summary of Stuructural Parameters of Dinuclear Complexes 4a–4f[a]

entry	compound	Au–C [Å]	Au–Br [Å]	C–Au–Br [°]	N–$C_{(Ar)}$ [Å]	N–$C_{(alk)}$ [Å]	N–$C_{(Ar)}$ [°]	Au–Au [Å]	C–C [Å][a]	N–N [Å][a]	N–N [Å][a]
1	**4a–Au1**	1.985	2.404	177.47	1.441	1.470	10.9	6.099	5.334	4.326	7.214
2	**4a–Au2**	1.976	2.396	172.09	1.439	1.464	4.3	-	-	-	-
3	**4b–Au1**	1.991	2.397	174.92	1.460	1.442	2.9	7.859	6.494	4.887	8.137
4	**4b–Au2**	1.998	2.399	174.84	1.429	1.454	8.0	-	-	-	-
5	**4c–Au1**	1.993	2.393	175.64	1.484	1.432	4.9	6.637	5.426	4.314	7.177
6	**4c–Au2**	1.981	2.406	178.79	1.433	1.477	2.5	-	-	-	-
7	**4d–Au1**	1.994	2.417	175.47	1.446	1.471	7.0	8.356	6.562	5.083	7.555
8	**4d–Au2**	1.991	2.397	174.8	1.445	1.474	8.6	-	-	-	-
9	**4e–Au1**	1.988	2.387	176.66	1.433	1.464	8.0	9.112	7.928	6.223	10.068
10	**4e–Au2**	1.988	2.387	176.66	1.433	1.464	8.0	-	-	-	-
11	**4f–Au1**	2.043	2.446	176.75	1.38	1.377	1.0	8.128	6.874	5.499	8.128
12	**4f–Au2**	1.890	2.290	177.91	1.449	1.439	0.5	-	-	-	-
13	**4g**	1.991	2.392	175.10	1.443	1.476	0.4	-	-	-	-
14[b]	**IPr***	1.987	2.273	178.35	1.430	-	1.0	-	-	-	-
15[c]	**IPr**	1.941	2.270	177.07	1.415	-	6.3	-	-	-	-
16[d]	**IPaul**	2.055	2.297	175.24	1.439	-	5.4	-	-	-	-

[a]$C1_{(carbene)}$–$C2_{(carbene)}$, $N1_{(proximal)}$–$N2_{(proximal)}$, $N1_{(distal)}$–$N2_{(distal)}$. [b][(IPr*)AuCl]. [c][(IPr)AuCl]. [d][(IPaul)AgCl].

Table S4. Summary of Stuructural Parameters of Dinuclear Complexes 4a–4f – %V_{bur}[a]

entry	compound	SW	NW	NE	SE	%V_{bur}
1	**4a–Au1**	49.7	26.3	56.5	70.5	50.8
2	**4a–Au2**	62.4	62	60.7	56.6	60.4
3	**4b–Au1**	29.1	25.4	52.2	39.8	36.6
4	**4b–Au2**	48.9	26.9	40.6	51.7	42.0
5	**4c–Au1**	25.2	36.1	34.3	47.2	35.7
6	**4c–Au2**	55.5	49.7	66.1	37.5	52.2
7	**4d–Au1**	26.5	39.9	70.4	57.7	48.6
8	**4d–Au2**	25.3	41.1	44.7	54.3	41.4
9	**4e–Au1**	25.6	29.8	52.3	53.0	40.2
10	**4e–Au2**	29.8	25.6	53	52.3	40.2
11	**4f–Au1**	27.5	25.1	36.2	46.7	33.9
12	**4f–Au2**	25.9	42.6	38.1	53.0	39.9
13	**4g**	25.2	46.8	46.6	52.3	42.7
14[b]	**IPr***	60.2	52.8	41.7	47.0	50.4
15[c]	**IPr**	49.4	42.3	50.3	40.6	45.7
16[d]	**IPaul**	54.3	29.6	32.9	56.2	43.3

[a]See, Cavallo, L. *Nat. Chem.* **2019**, *11*, 872-879. [b][(IPr*)AuCl]. [c][(IPr)AuCl]. [d][(IPaul)AgCl].

Additional Discussion Regarding HOMO and LUMO Energies. To gain insight into the electronic properties of these dinuclear IPr*^{diNHC} ligands, we determined HOMO and LUMO energy levels at the B3LYP 6-311++g(d,p) level (Fig. S2 and Chart S1). The parent IPr*^{diNHC} carbenes (**3a**) and (**3b**) corresponding to the monomeric IPr* the mononuclear IPr*$^{n\text{-Bu}}$ carbene (**3g**) were used as model systems. The s-donor orbital of IPr*$^{diNHC–C3}$ (**3a**) (HOMO-1 due to required symmetry, -5.92 eV, linear anti geometry, 75.7° conformation between the carbene donors) is in the same range as the s-donor orbital of IPr*$^{diNHC–C4}$ (**3b**) (-5.90 eV, linear anti geometry, 0.0° conformation between the carbene donors) and the mononuclear IPr*$^{n\text{-Bu}}$ (**3g**) (-5.85 eV). These values can be compared with IPr* (-6.12 eV) and the classical IPr (-6.01 eV). The □-accepting orbital of IPr*$^{diNHC–C3}$ (**3a**) (-0.80 eV) is at the same level as that of IPr*$^{diNHC–C4}$ (**3b**) (-0.81 eV) and of the mononuclear IPr*$^{n\text{-Bu}}$ (**3g**) (-0.80 eV). These values can be compared with IPr* (-0.90 eV) and IPr (-0.48 eV). Furthermore, the p-donor orbital of IPr*$^{diNHC–C3}$ (**3a**) (HOMO, -5.88 eV) is at same level as that of IPr*$^{diNHC–C4}$ (**3b**) (HOMO-3, -5.93 eV) and of the mononuclear IPr*$^{n\text{-Bu}}$ (**3g**) (HOMO-1, -5.89 eV). These values can be compared with the corresponding p-donor orbitals of IPr* (-6.28 eV) and IPr (-6.55 eV). Thus, the results indicate that (1) IPr*^{diNHC} ligands are electronically characterized as strongly σ-nucleophilic ligands, (2) the steric impact arises from the dinuclear rotationally-adaptable imidazol-2-ylidene framework.

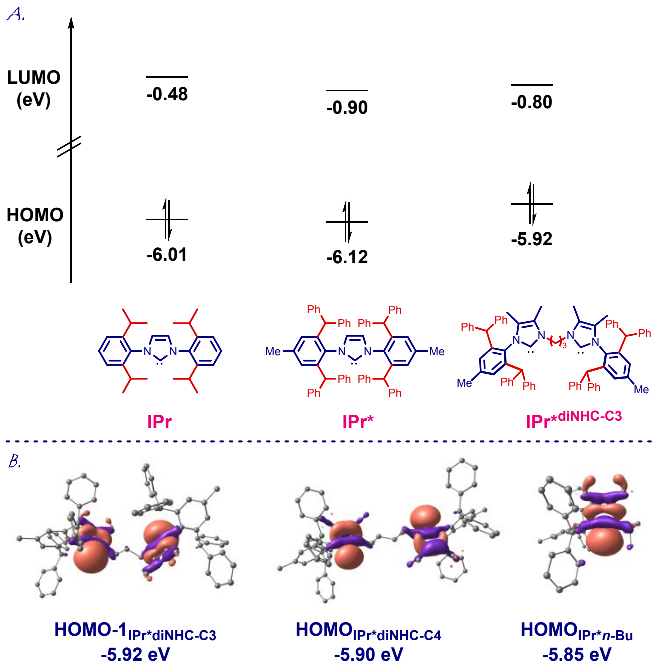

Fig. S2. (A) HOMO and LUMO energies (eV). (B) s-Donor orbitals of IPr*$^{diNHC-C3}$, IPr*$^{diNHC-C4}$ and IPr*$^{n\text{-Bu}}$ (eV) calculated at B3LYP 6-311++g(d,p).

Computational Methods

All of the calculations were performed using Gaussian 09 suite of programs. All of the geometry optimizations were performed at the B3LYP level of theory in the gas phase with the 6-311++G(d,p) basis set. For geometry optimizations, we employed the X-ray structures of 1-(2,6-dibenzhydryl-4-methylphenyl)-3-(3-(1-(2,6-dibenzhydryl-4-methylphenyl)-4,5-dimethyl-1*H*-imidazol-3-ium-3-yl)propyl)-4,5-dimethyl-1*H*-imidazol-3-ium, 1-(2,6-dibenzhydryl-4-methylphenyl)-3-(4-(1-(2,6-dibenzhydryl-4-methylphenyl)-4,5-dimethyl-1*H*-imidazol-3-ium-3-yl)butyl)-4,5-dimethyl-1*H*-imidazol-3-ium and 3-butyl-1-(2,6-dibenzhydryl-4-methylphenyl)-4,5-dimethyl-1*H*-imidazol-3-ium and their linear metal complexes as the starting geometry and performed full optimization. The absence of imaginary frequencies was used to characterize the structures as minima on the potential energy surface. All of the optimized geometries were verified as minima (no imaginary frequencies). Energetic parameters were calculated under standard conditions (298.15 K and 1 atm). Structural representations were generated using CYLview software (Legault, C. Y. CYLview version 1.0 BETA, University of Sherbrooke). All other representations were generated using GaussView (GaussView, version 5, Dennington, R.; Keith, T.; Millam, J. Semichem Inc., Shawnee Mission, KS, 2009) or ChemCraft software (Andrienko, G. L. ChemCraft version b562a, https://www.chemcraftprog.com).

Full Reference for Gaussian 09

Gaussian 09, Revision D.01, Frisch, M. J.; Trucks, G. W.; Schlegel, H. B.; Scuseria, G. E.; Robb, M. A.; Cheeseman, J. R.; Scalmani, G.; Barone, V.; Mennucci, B.; Petersson, G. A.; Nakatsuji, H.; Caricato, M.; Li, X.; Hratchian, H. P.; Izmaylov, A. F.; Bloino, J.; Zheng, G.; Sonnenberg, J. L.; Hada, M.; Ehara, M.; Toyota, K.; Fukuda, R.; Hasegawa, J.; Ishida, M.; Nakajima, T.; Honda, Y.; Kitao, O.; Nakai, H.; Vreven, T.; Montgomery, J. A., Jr.; Peralta, J. E.; Ogliaro, F.; Bearpark, M.; Heyd, J. J.; Brothers, E.; Kudin, K. N.; Staroverov, V. N.; Kobayashi, R.; Normand, J.; Raghavachari, K.; Rendell, A.; Burant, J. C.; Iyengar, S. S.; Tomasi, J.; Cossi, M.; Rega, N.; Millam, M. J.; Klene, M.; Knox, J. E.; Cross, J. B.; Bakken, V.; Adamo, C.; Jaramillo, J.; Gomperts, R.; Stratmann, R. E.; Yazyev, O.; Austin, A. J.; Cammi, R.; Pomelli, C.; Ochterski, J. W.; Martin, R. L.; Morokuma, K.; Zakrzewski, V. G.; Voth, G.

A.; Salvador, P.; Dannenberg, J. J.; Dapprich, S.; Daniels, A. D.; Farkas, Ö.; Foresman, J. B.; Ortiz, J. V.; Cioslowski, J.; Fox, D. J. Gaussian, Inc., Wallingford CT, 2009.

Chart S1. HOMO and LUMO Energy Levels of Dinuclear and Related NHC Ligands Calculated at the B3LYP 6-311++g(d,p) Level[a,b]

entry	compound	orbital	E [au]	E [eV]	E [kcal/mol]	□□□ [eV]
1	IPr*$^{\text{diNHC–C3}}$	HOMO-1	-0.2174	-5.92	-136.42	
2	IPr*$^{\text{diNHC–C3}}$	HOMO	-0.2160	-5.88	-135.54	
3	IPr*$^{\text{diNHC–C3}}$	LUMO	-0.0295	-0.80	-18.51	-5.07
4[a]	IPr*$^{\text{diNHC–C4}}$	HOMO-3	-0.2180	-5.93	-136.80	
5	IPr*$^{\text{diNHC–C4}}$	HOMO	-0.2167	-5.90	-135.98	
6	IPr*$^{\text{diNHC–C4}}$	LUMO	-0.0299	-0.81	-18.76	-5.08
7	IPr*$^{n\text{-Bu}}$	HOMO	-0.2166	-5.89	-135.92	
8	IPr*$^{n\text{-Bu}}$	HOMO	-0.2151	-5.85	-134.98	
9	IPr*$^{n\text{-Bu}}$	LUMO	-0.2166	-0.80	-18.45	-5.05
10	IPr*	HOMO-1	-0.2309	-6.28	-144.89	
11	IPr*	HOMO	-0.2249	-6.12	-141.12	
12	IPr*	LUMO	-0.0330	-0.90	-20.71	-5.22
13	IPr	HOMO-1	-0.2406	-6.55	-150.98	
14	IPr	HOMO	-0.2210	-6.01	-139.78	
15[b]	IPr	LUMO+1	-0.0177	-0.48	-12.21	-5.53

[a]HOMO–3 due to required orbital symmetry. [b]LUMO+1 due to required orbital symmetry. See, Falivene, L.; Cavallo, L. *Coord. Chem. Rev.* **2017**, *344*, 101-114.

^{1}H and ^{13}C NMR Spectra

1a 2,6-Dibenzhydryl-4-methylaniline

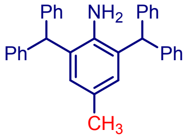

1a

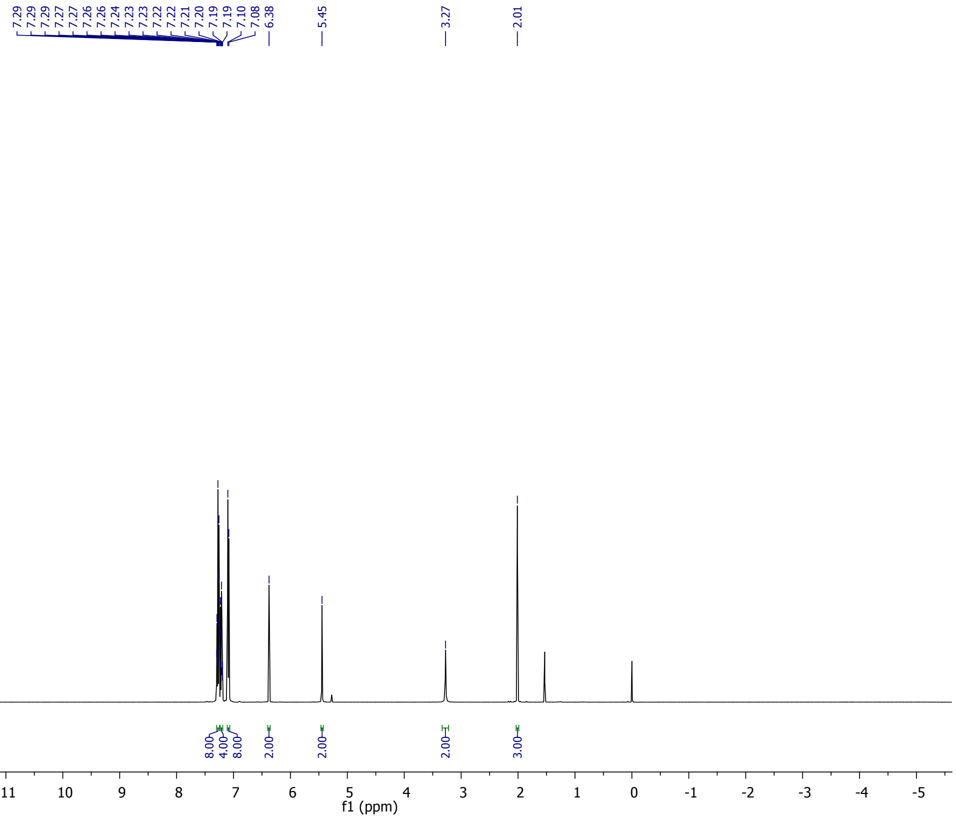

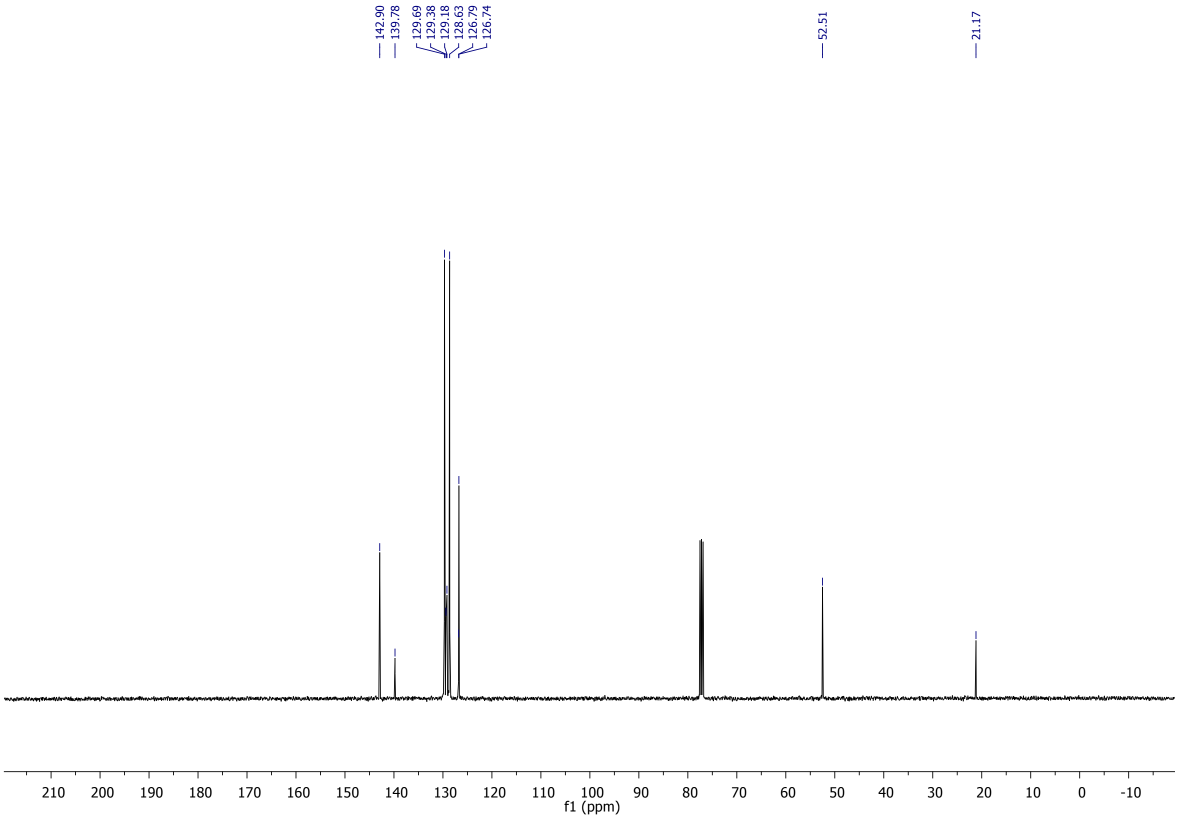

1b 4-Methoxy-2,6-bis(diphenylmethyl)aniline

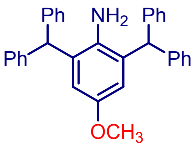

1b

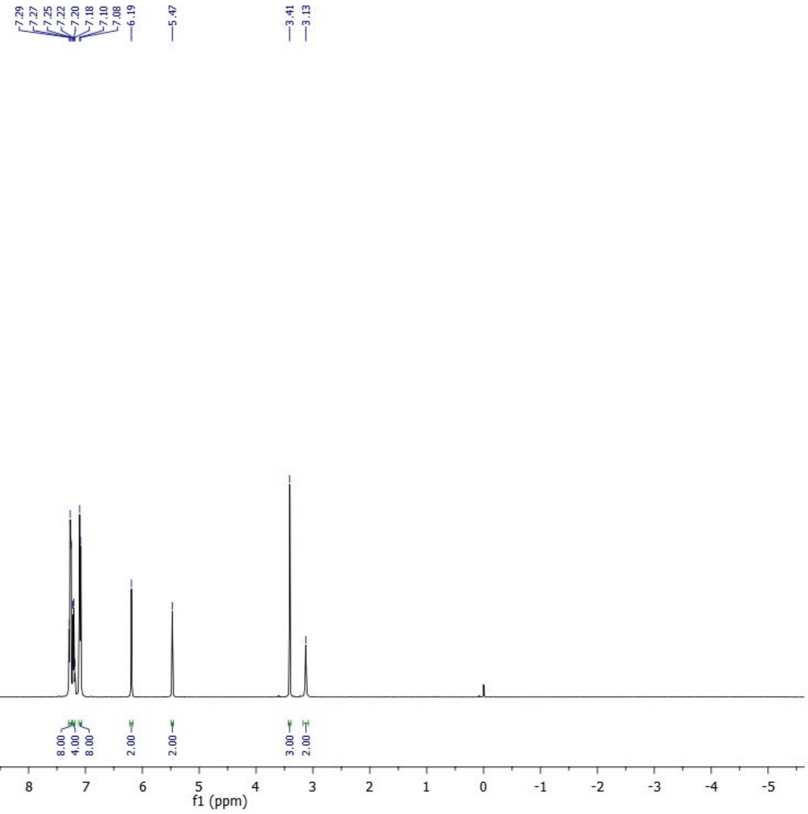

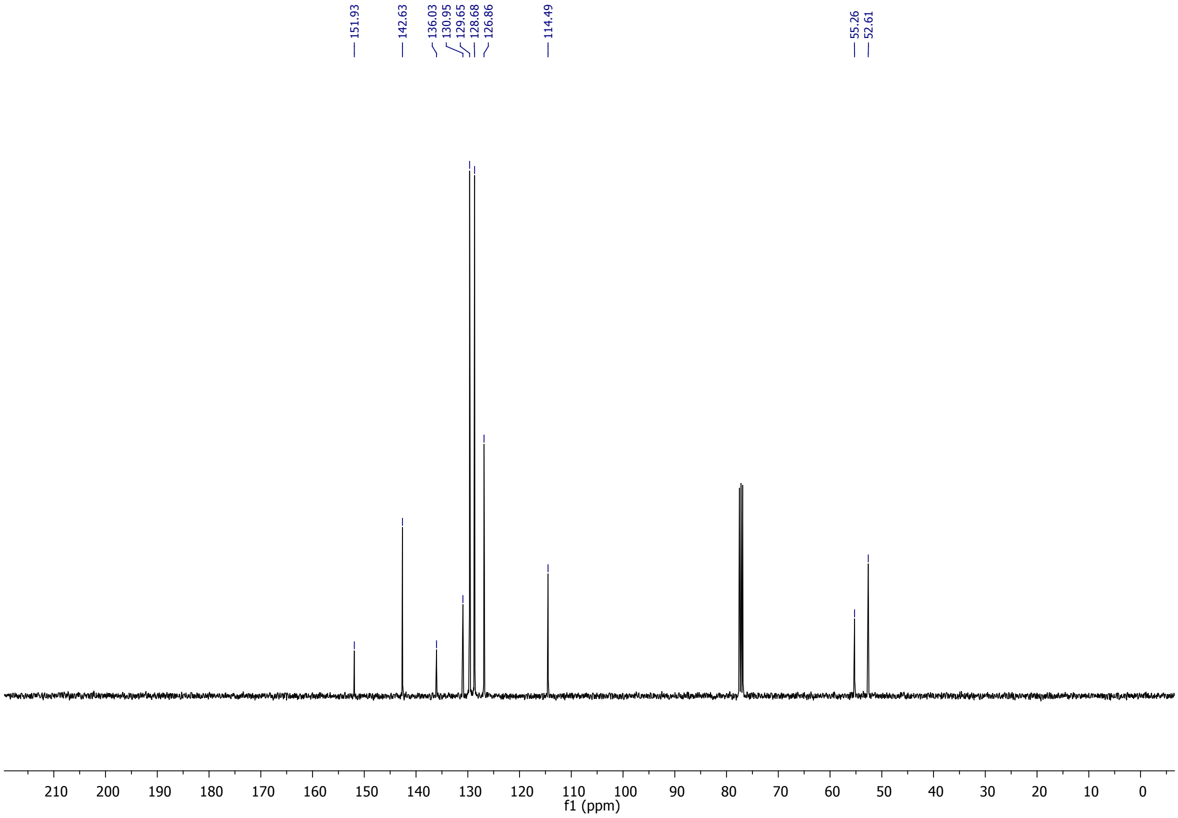

1c 2-Benzhydryl-4,6-dimethylaniline

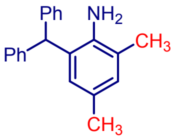

1c

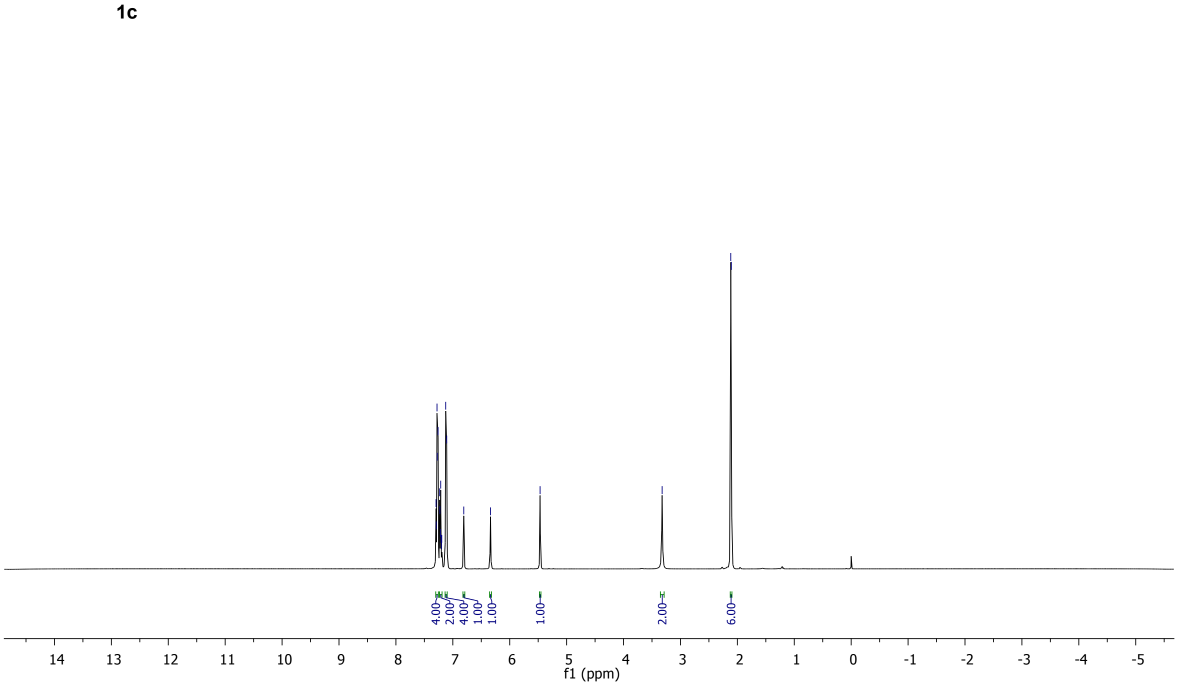

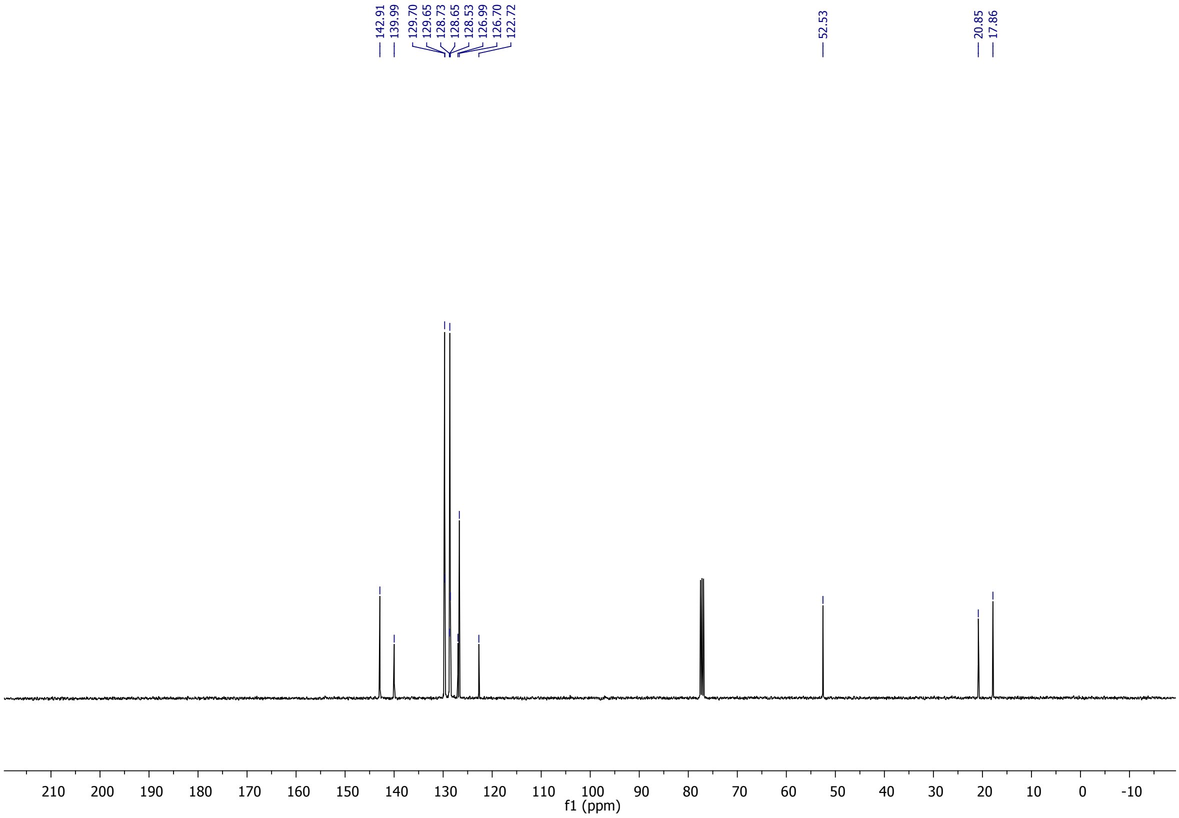

2a 1-(2,6-Dibenzhydryl-4-methylphenyl)-4,5-dimethyl-1H-imidazole

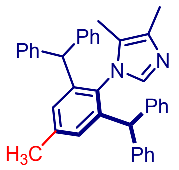

2a

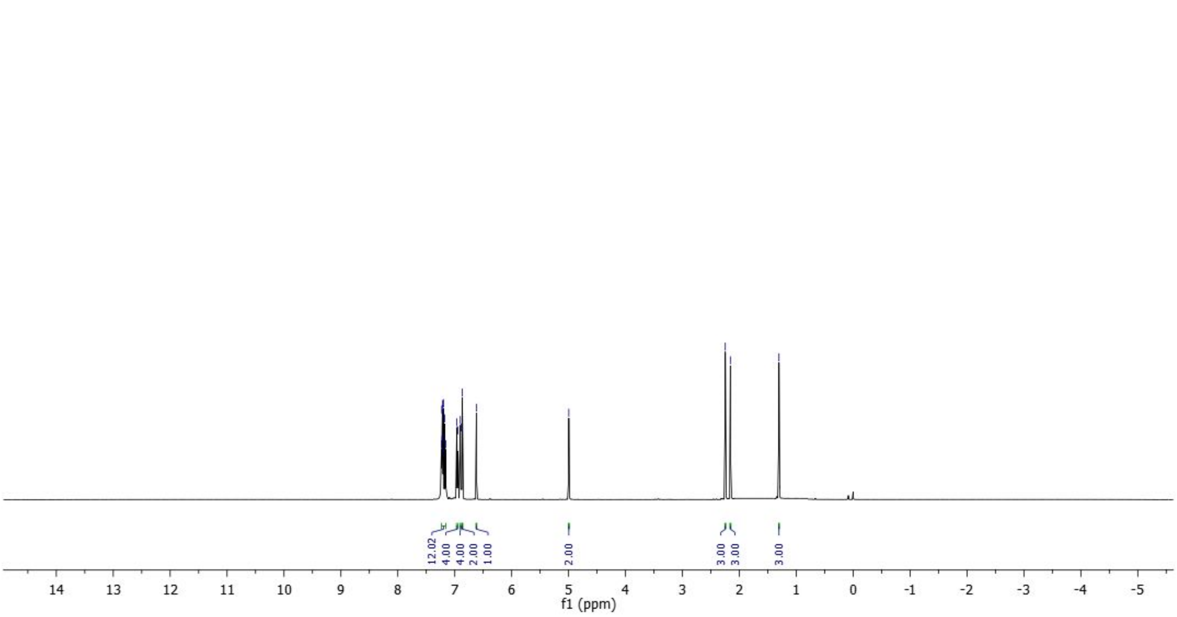

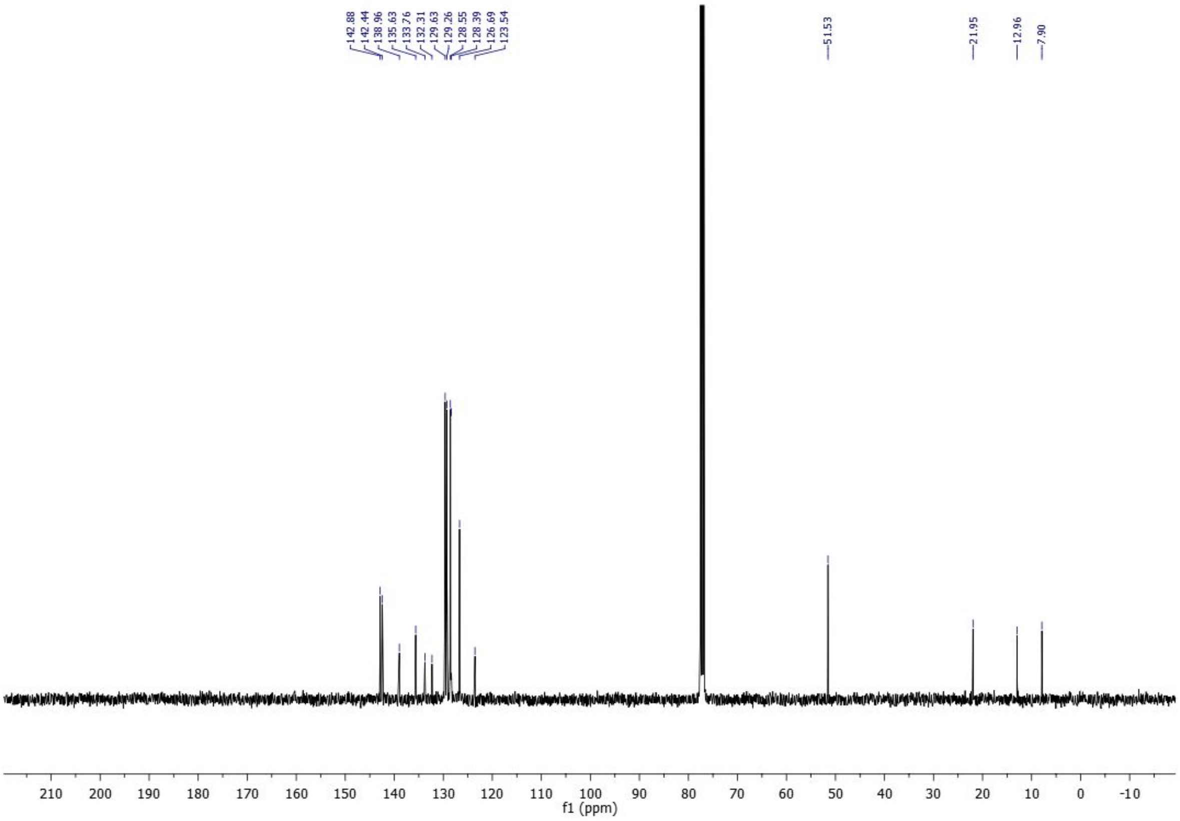

2b 1-(2,6-Dibenzhydryl-4-methoxyphenyl)-4,5-dimethyl-1H-imidazole

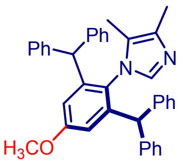

2b

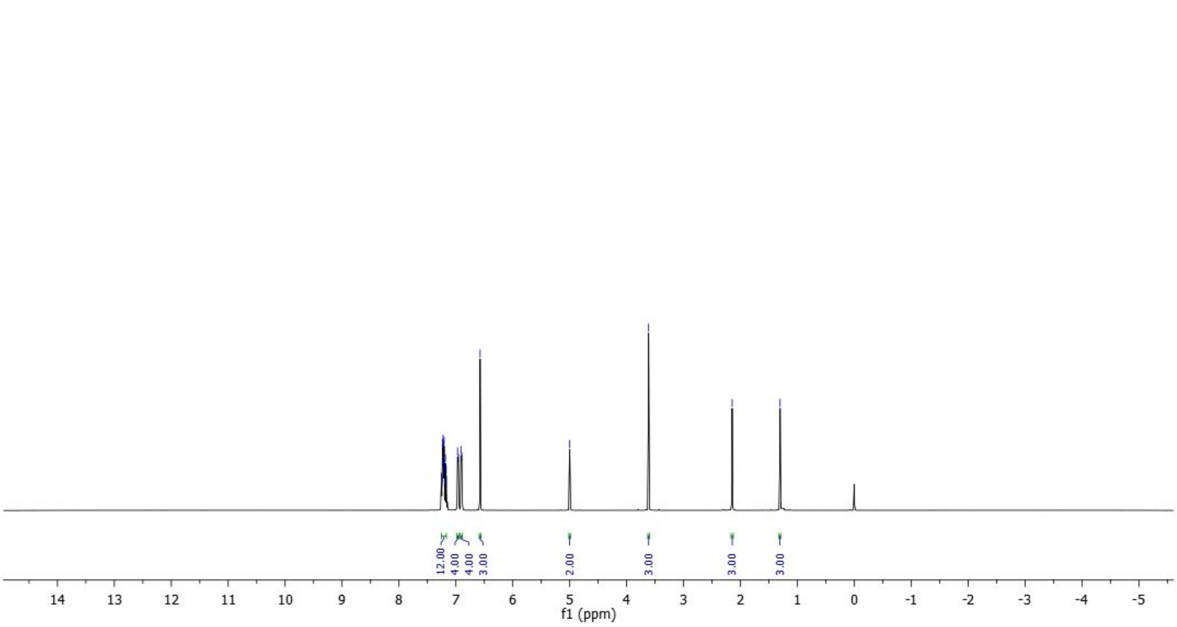

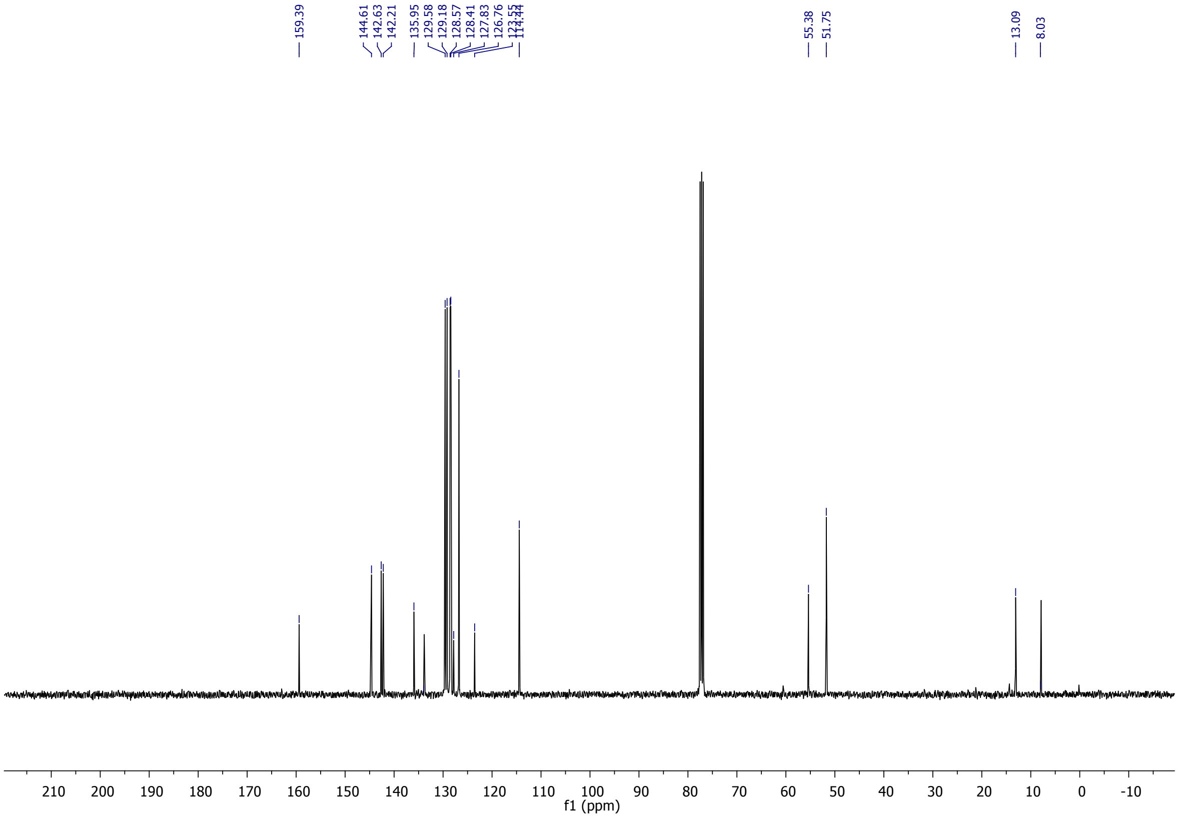

2c 1-(2-benzhydryl-4,6-dimethylphenyl)-4,5-dimethyl-1H-imidazole

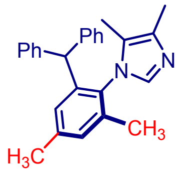

2c

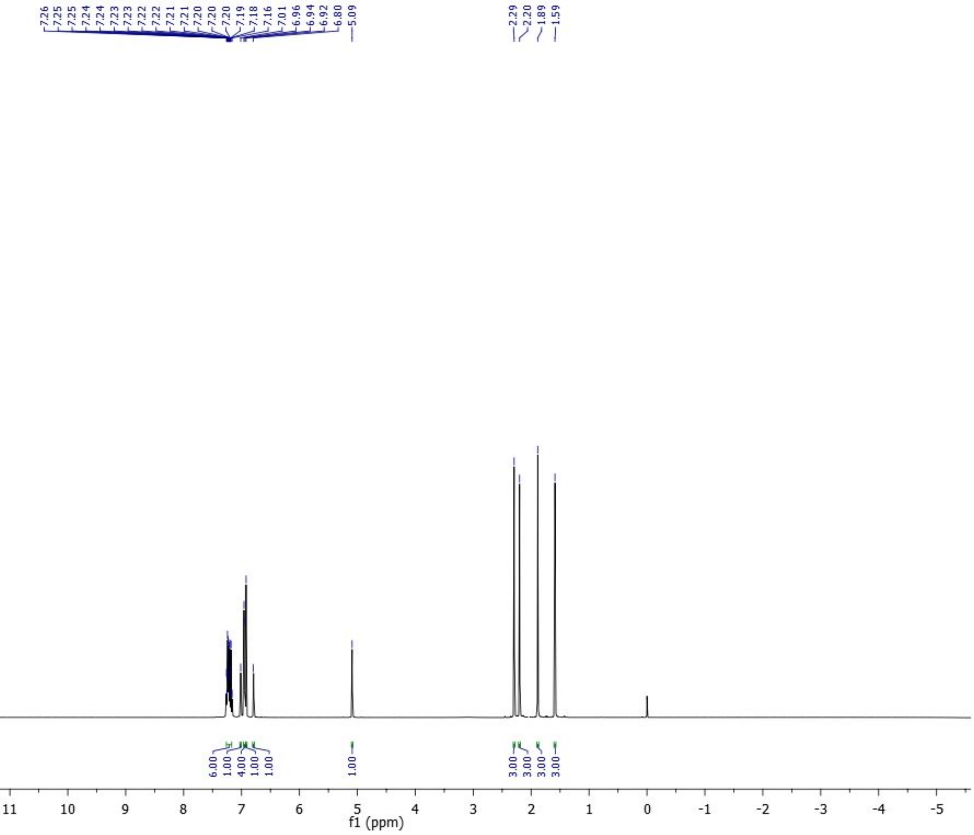

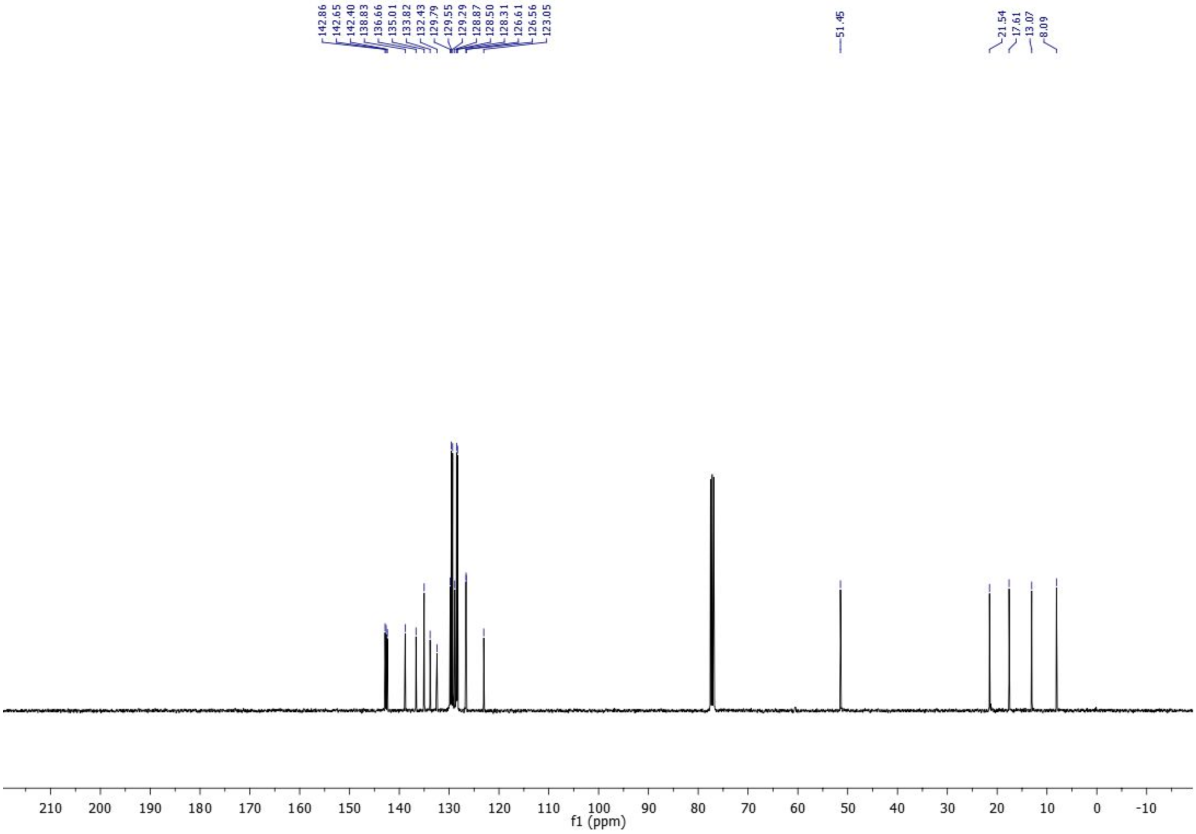

3a 3,3'-(Propane-1,3-diyl)bis(1-(2,6-dibenzhydryl-4-methylphenyl)-4,5-dimethyl-1H-imidazol-3-ium) Dichloride

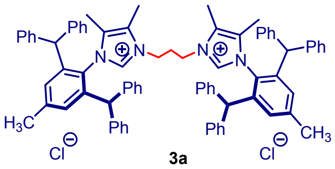

3a

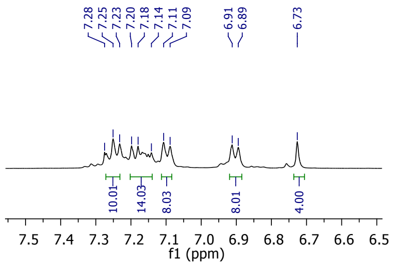

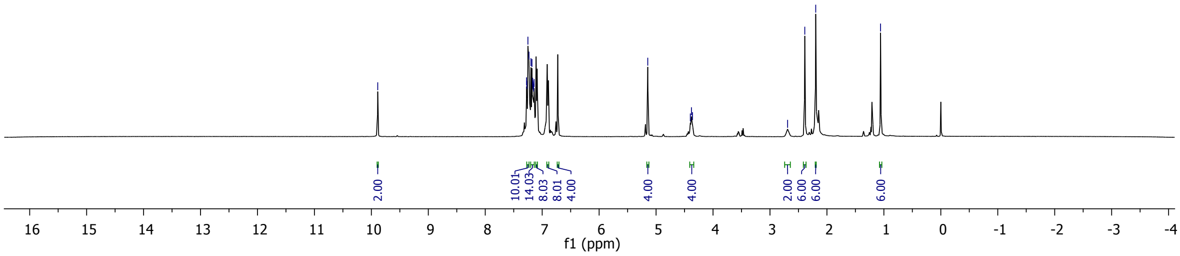

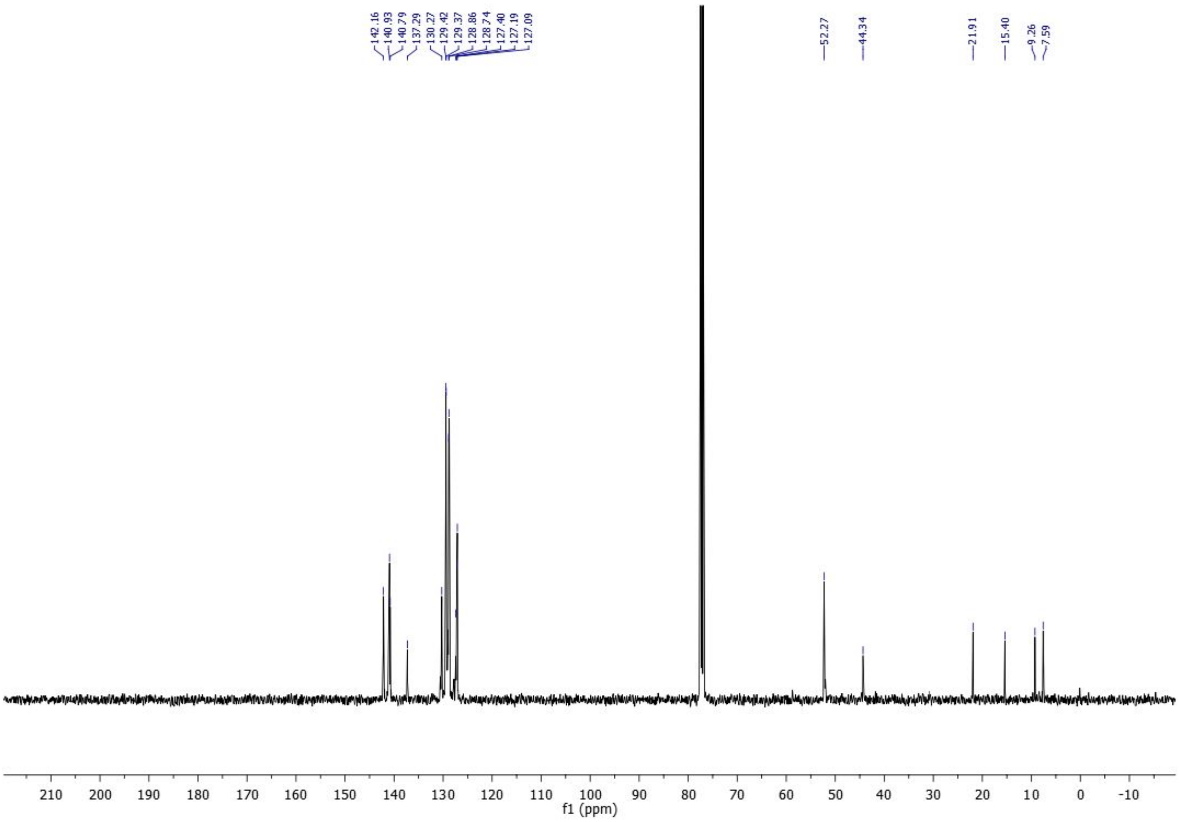

3b 3,3'-(Propane-1,3-diyl)bis(1-(2,6-dibenzhydryl-4-methoxyphenyl)-4,5-dimethyl-1H-imidazol-3-ium) Dichloride

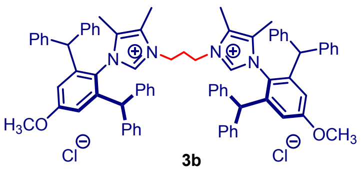

3b

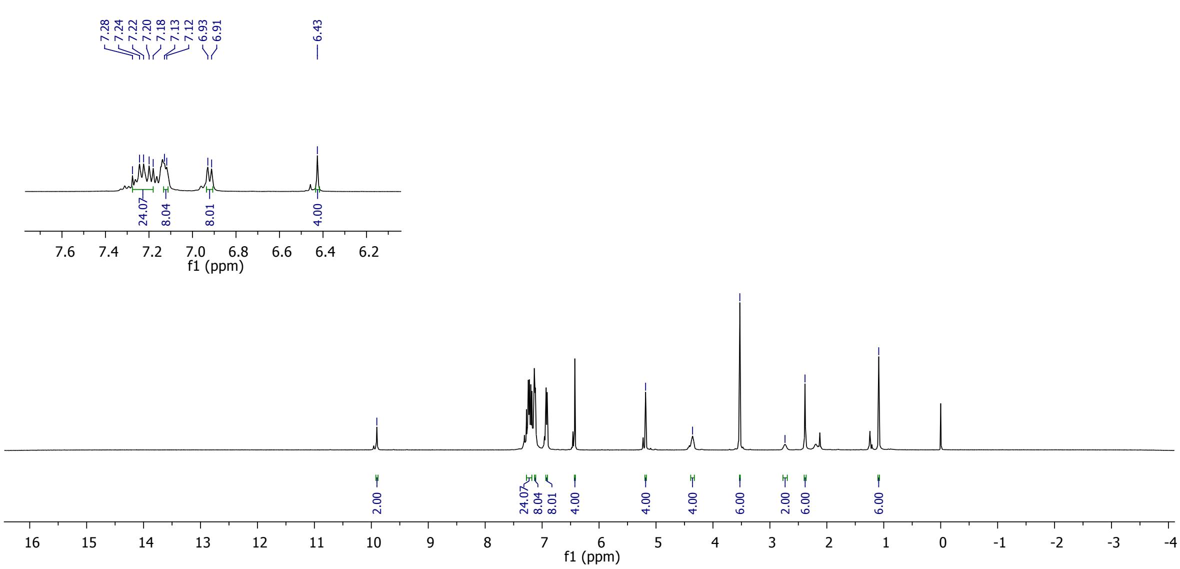

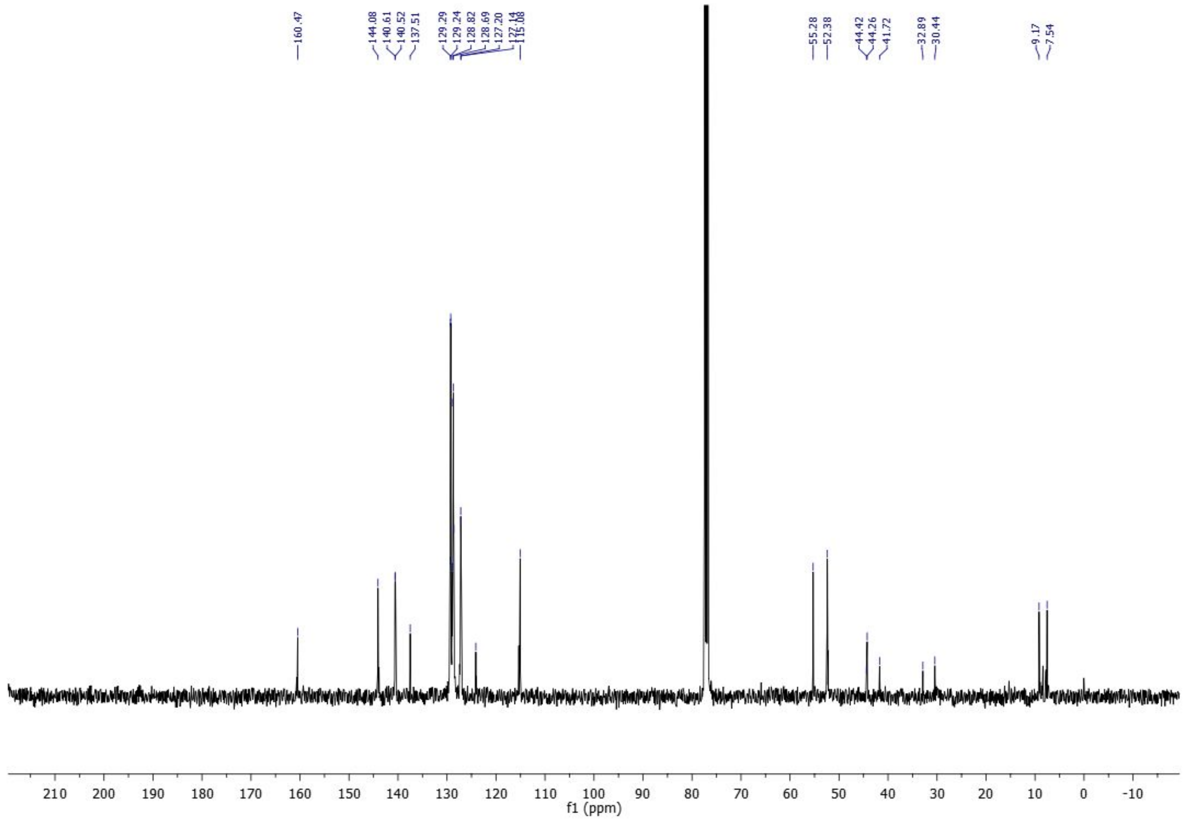

3c 3,3'-(Propane-1,3-diyl)bis(1-(2-benzhydryl-4,6-dimethylphenyl)-4,5-dimethyl-1H-imidazol-3-ium) Dichloride

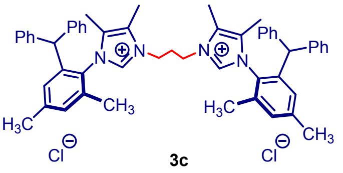

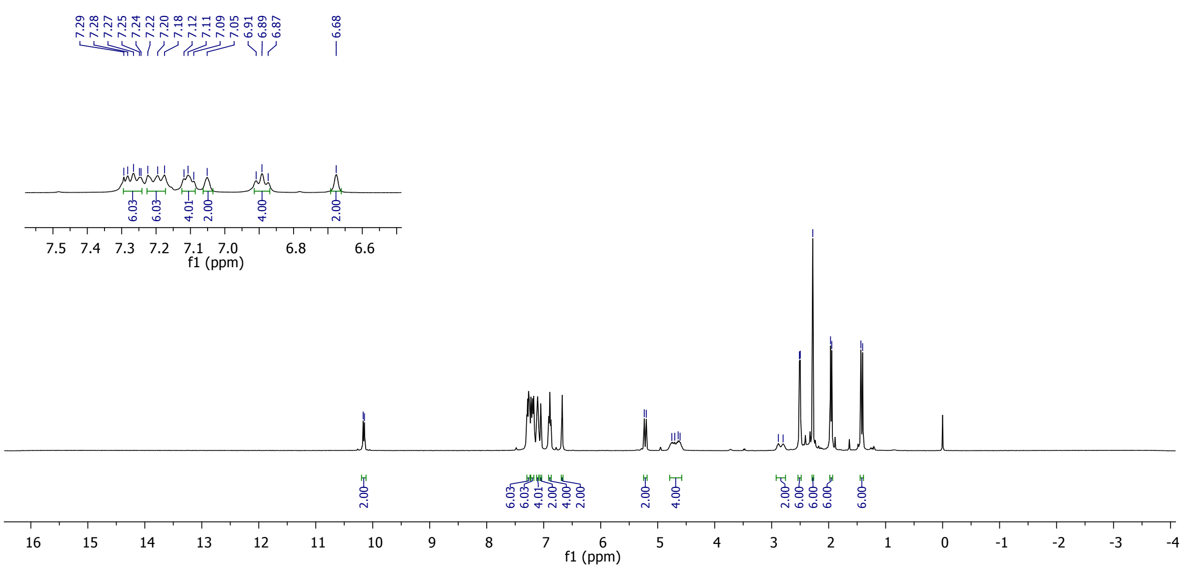

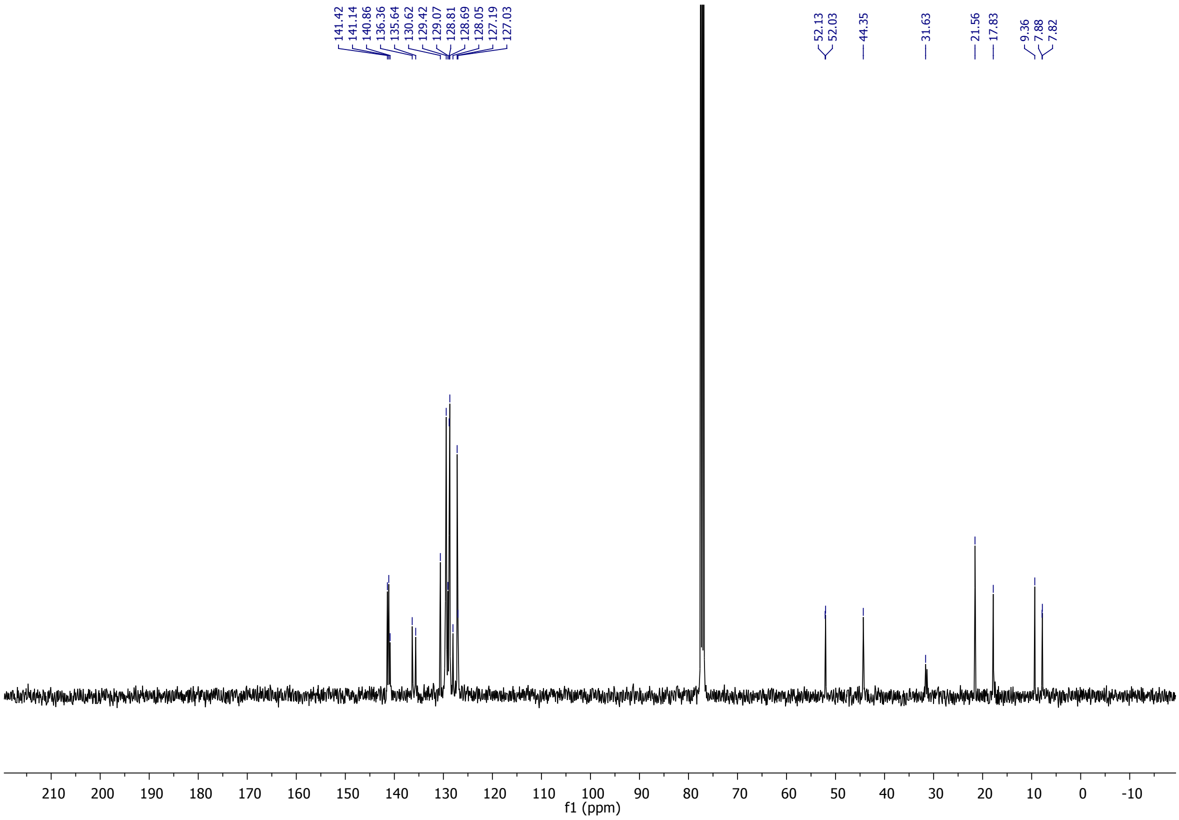

3d 3,3'-(Butane-1,4-diyl)bis(1-(2,6-dibenzhydryl-4-methylphenyl)-4,5-dimethyl-1H-imidazol-3-ium) Dichloride

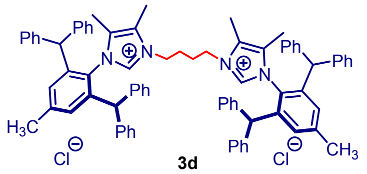

3d

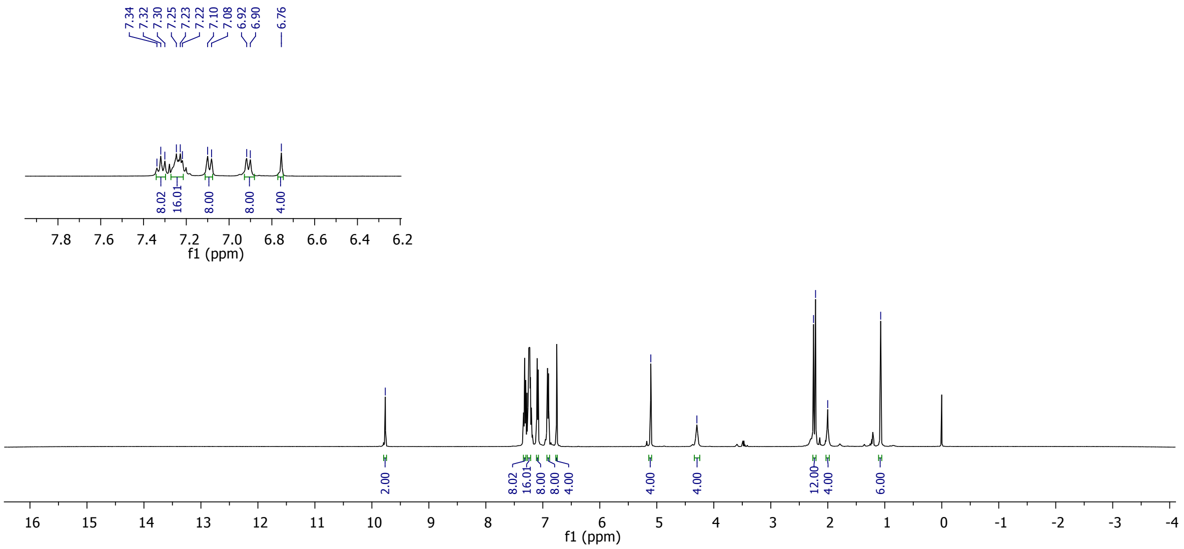

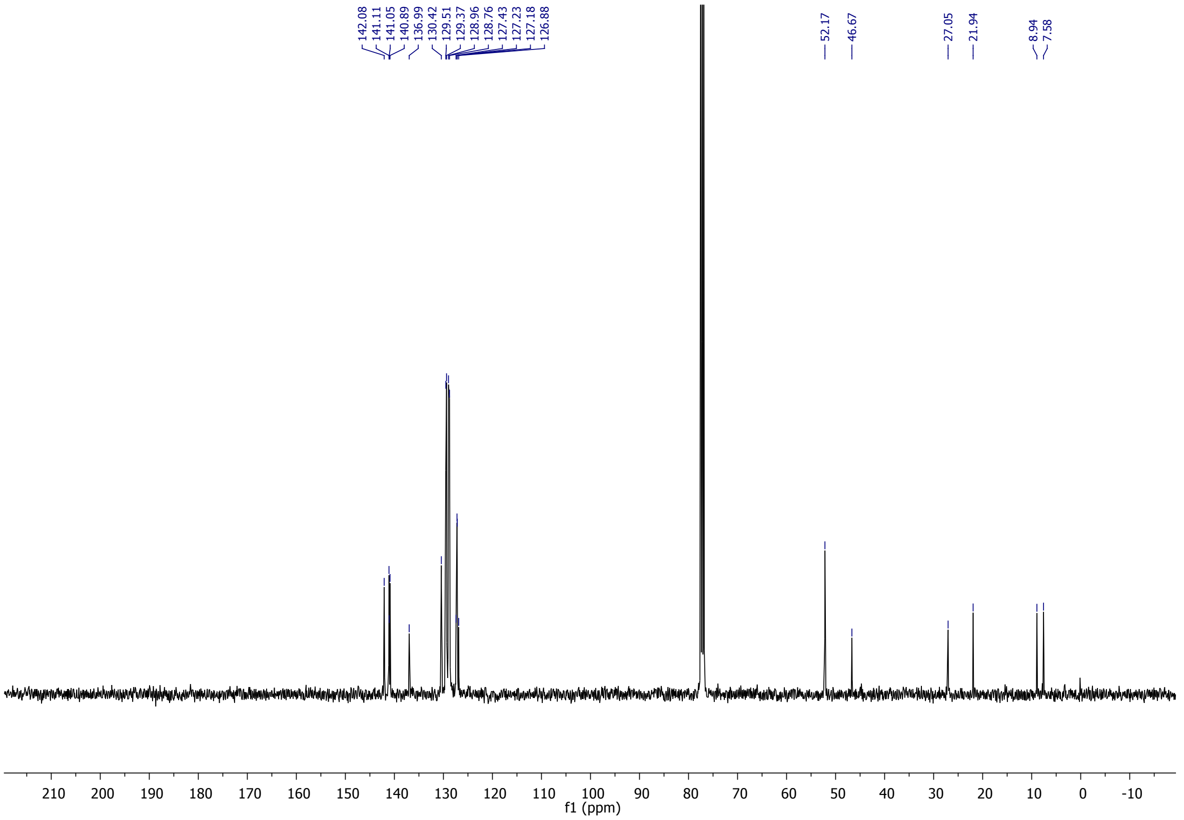

3e 3,3'-(Butane-1,4-diyl)bis(1-(2,6-dibenzhydryl-4-methoxyphenyl)-4,5-dimethyl-1H-imidazol-3-ium) Dichloride

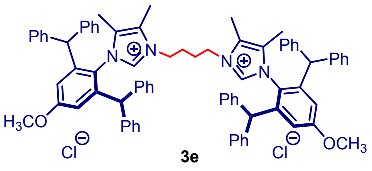

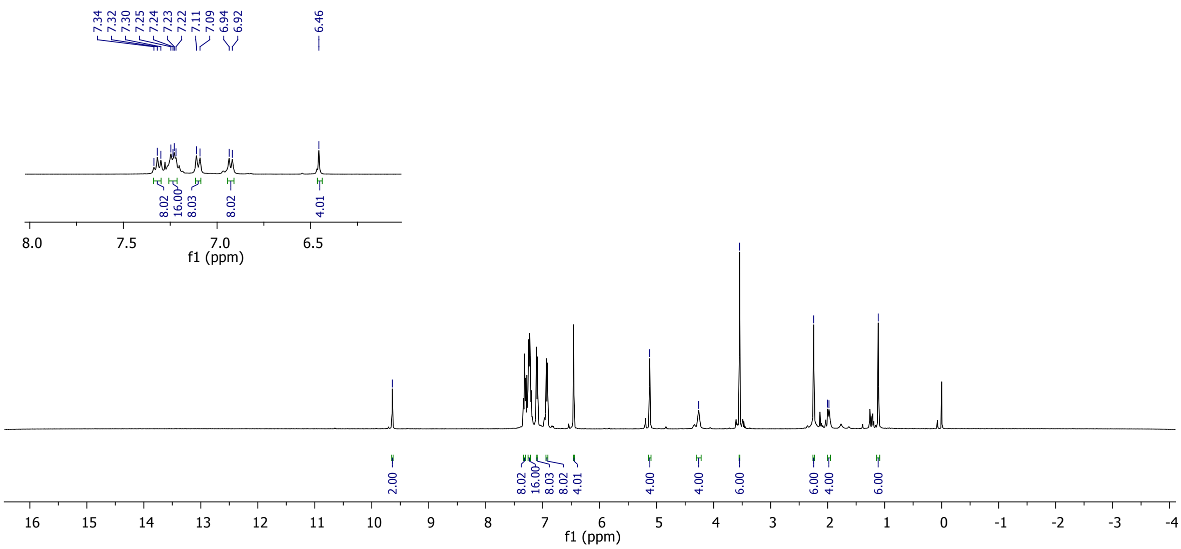

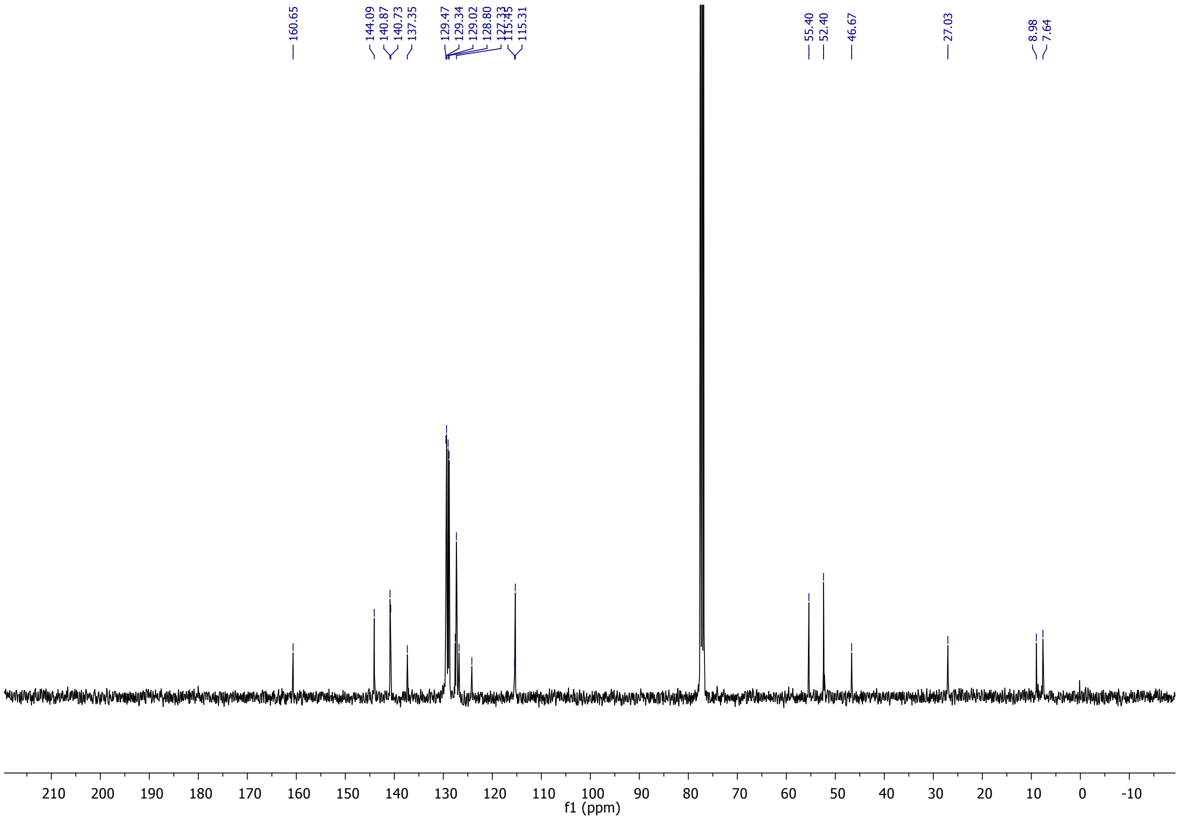

3f 3,3'-(Butane-1,4-diyl)bis(1-(2-benzhydryl-4,6-dimethylphenyl)-4,5-dimethyl-1H-imidazol-3-ium) Dichloride

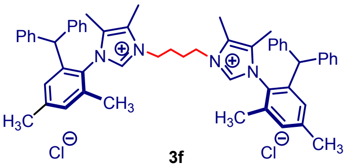

3f

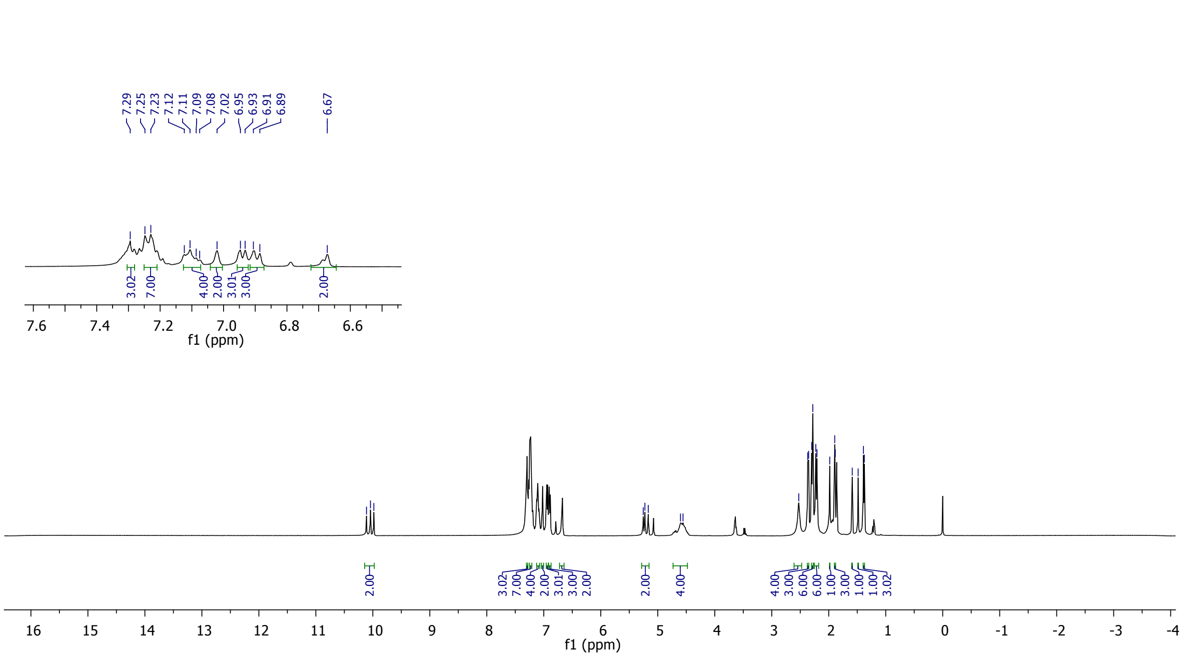

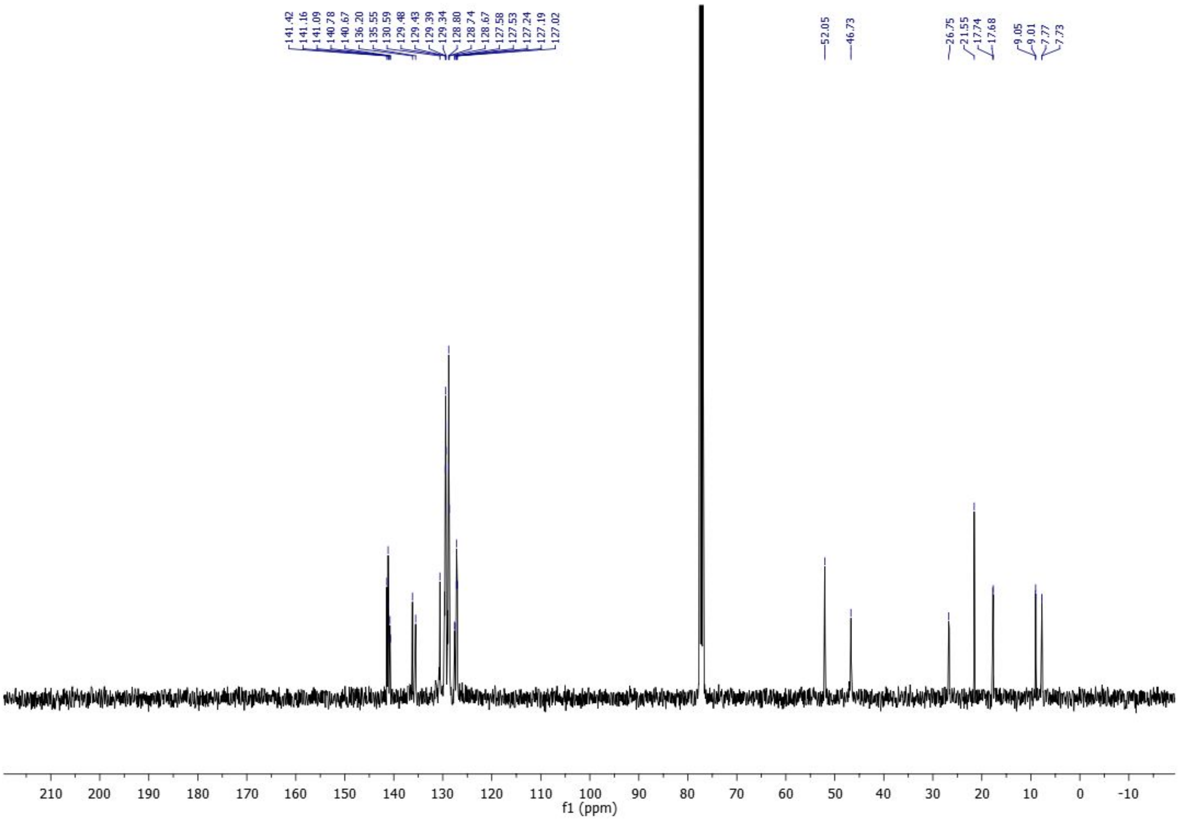

3g 3-Butyl-1-(2,6-dibenzhydryl-4-methylphenyl)-4,5-dimethyl-1H-imidazol-3-ium Bromide

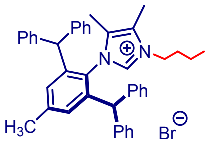

3g

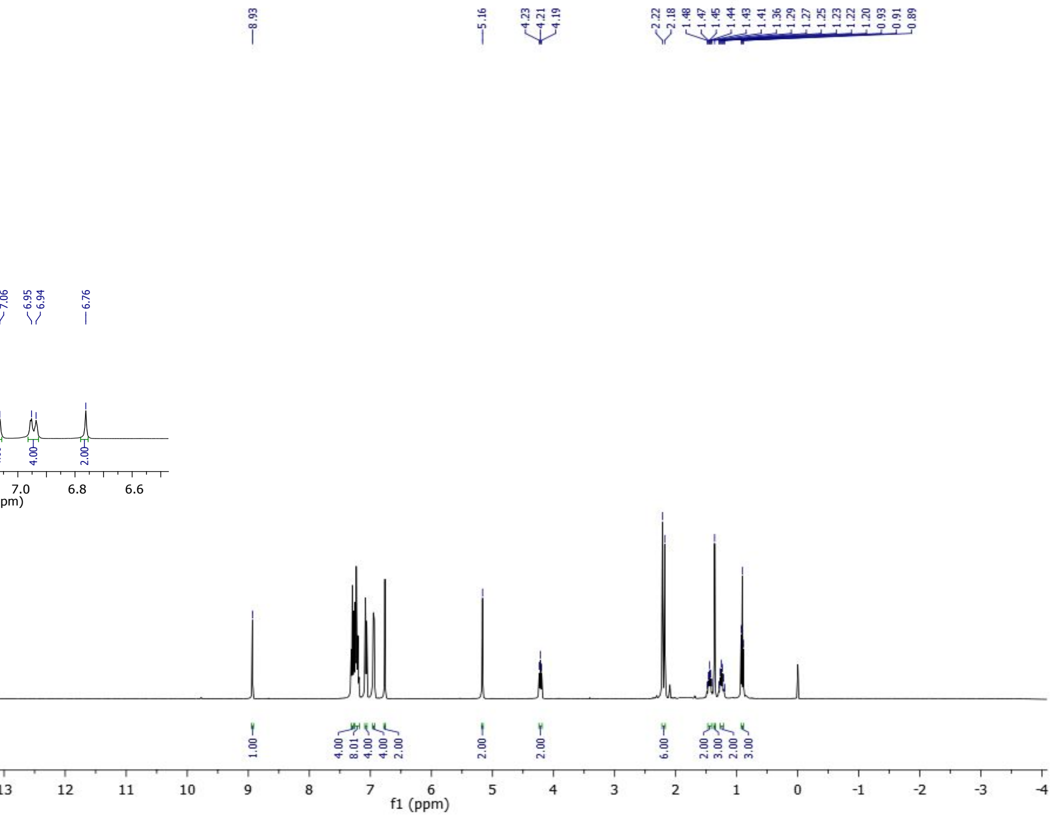

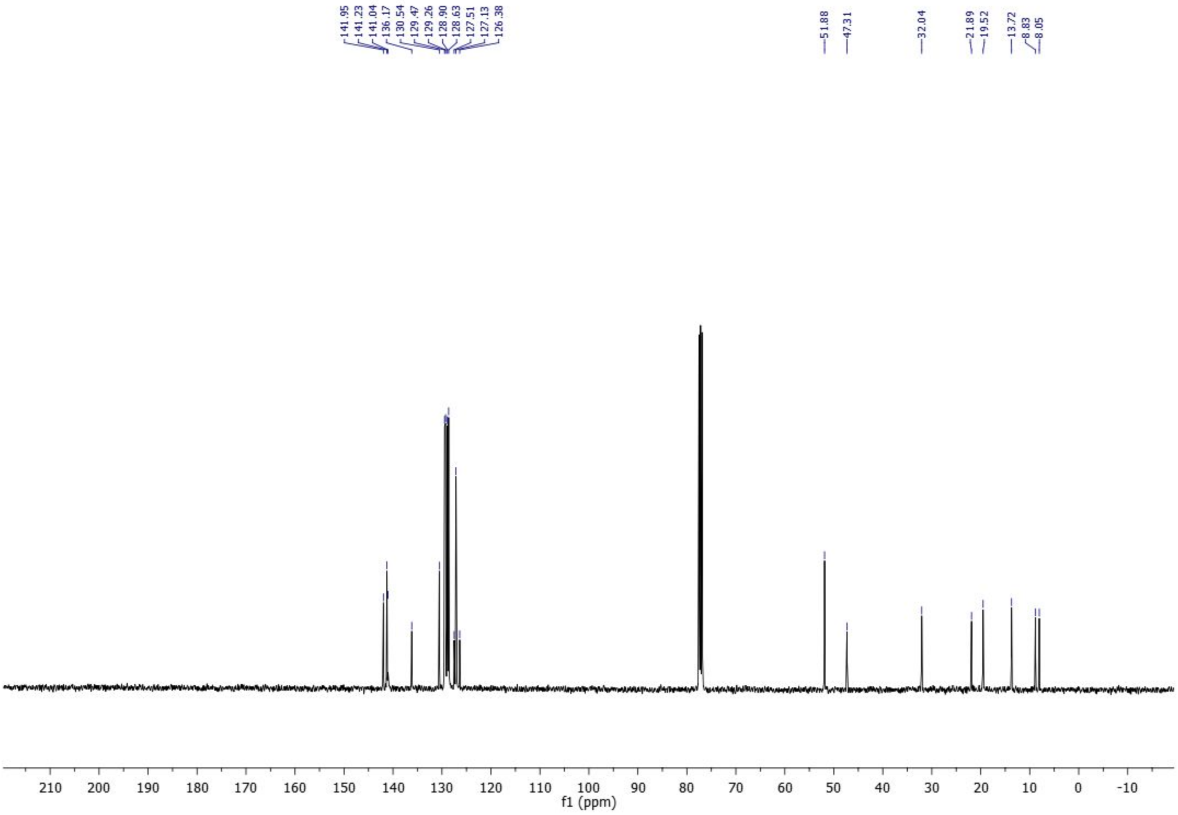

Au_2Br_24a

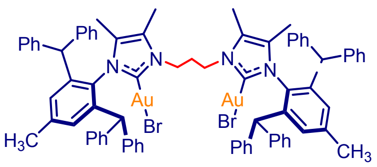

Au_2Br_24a

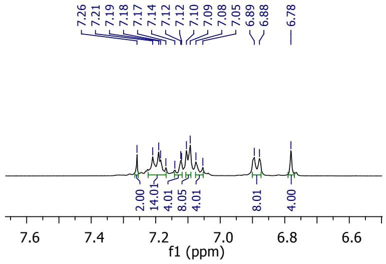

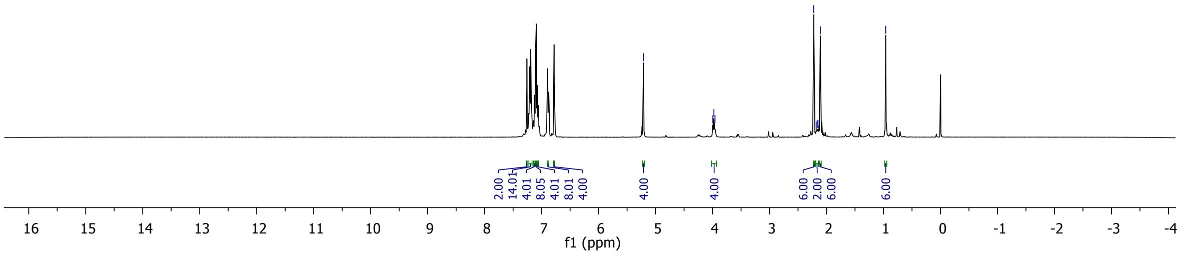

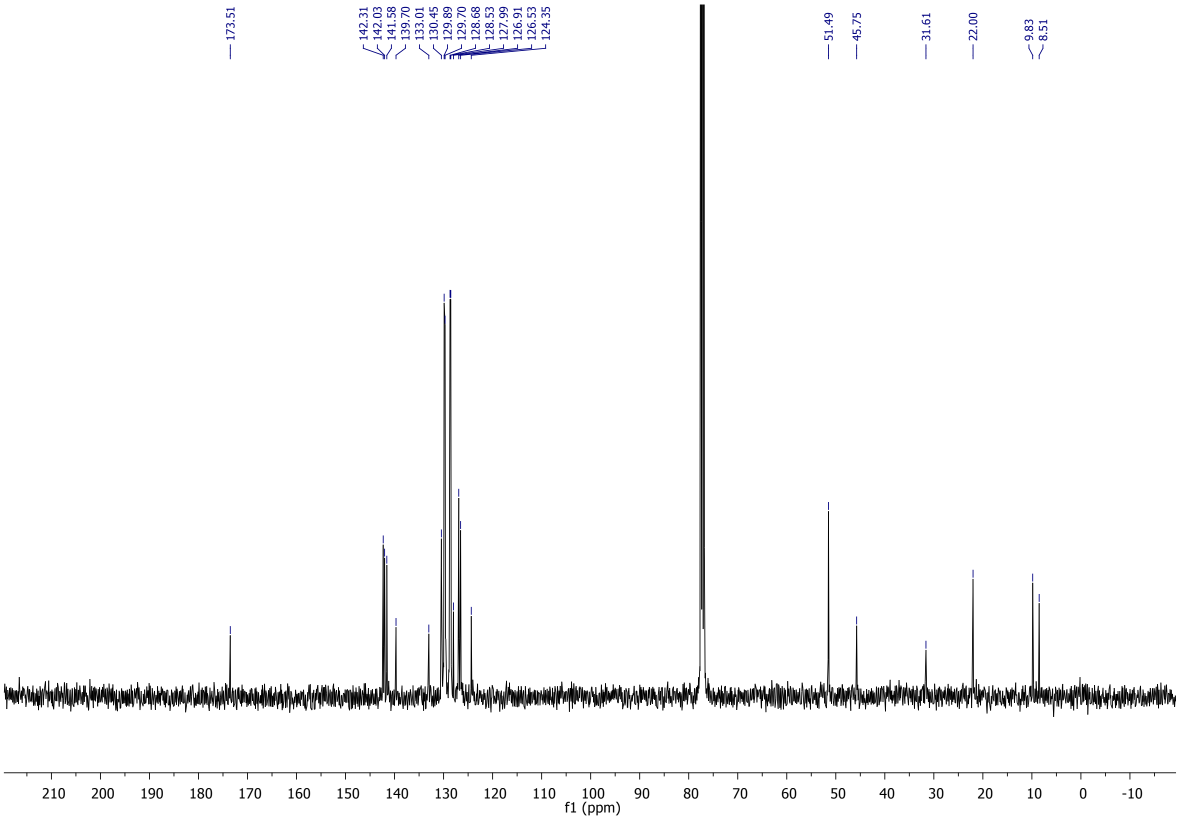

Au_2Br_24b

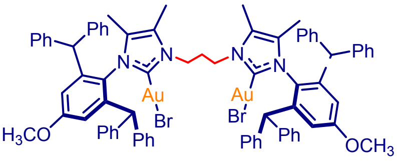

Au_2Br_24b

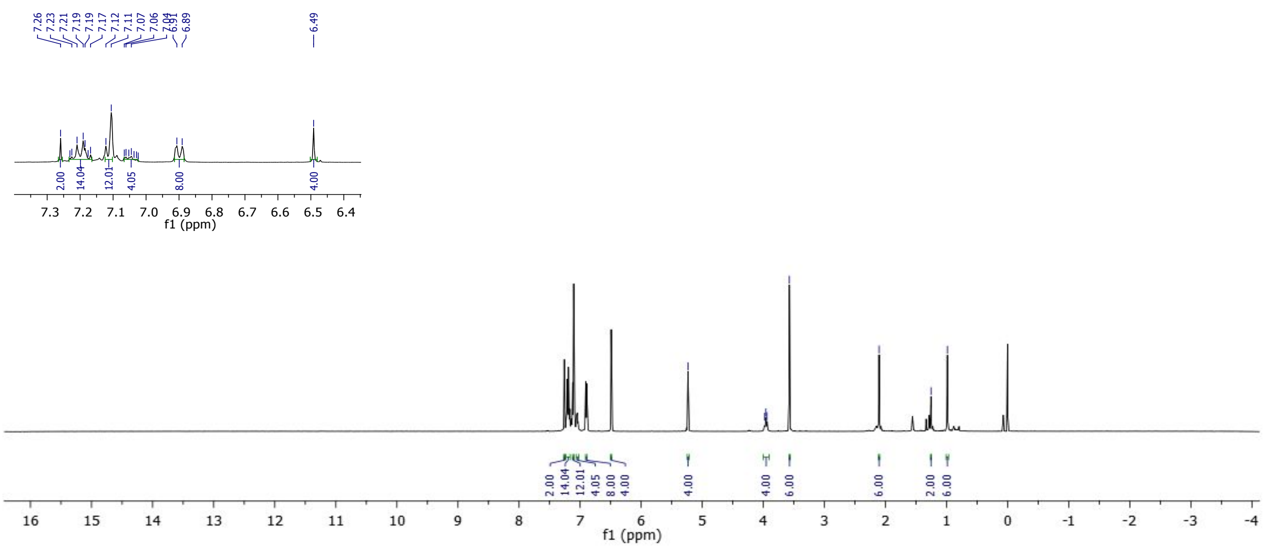

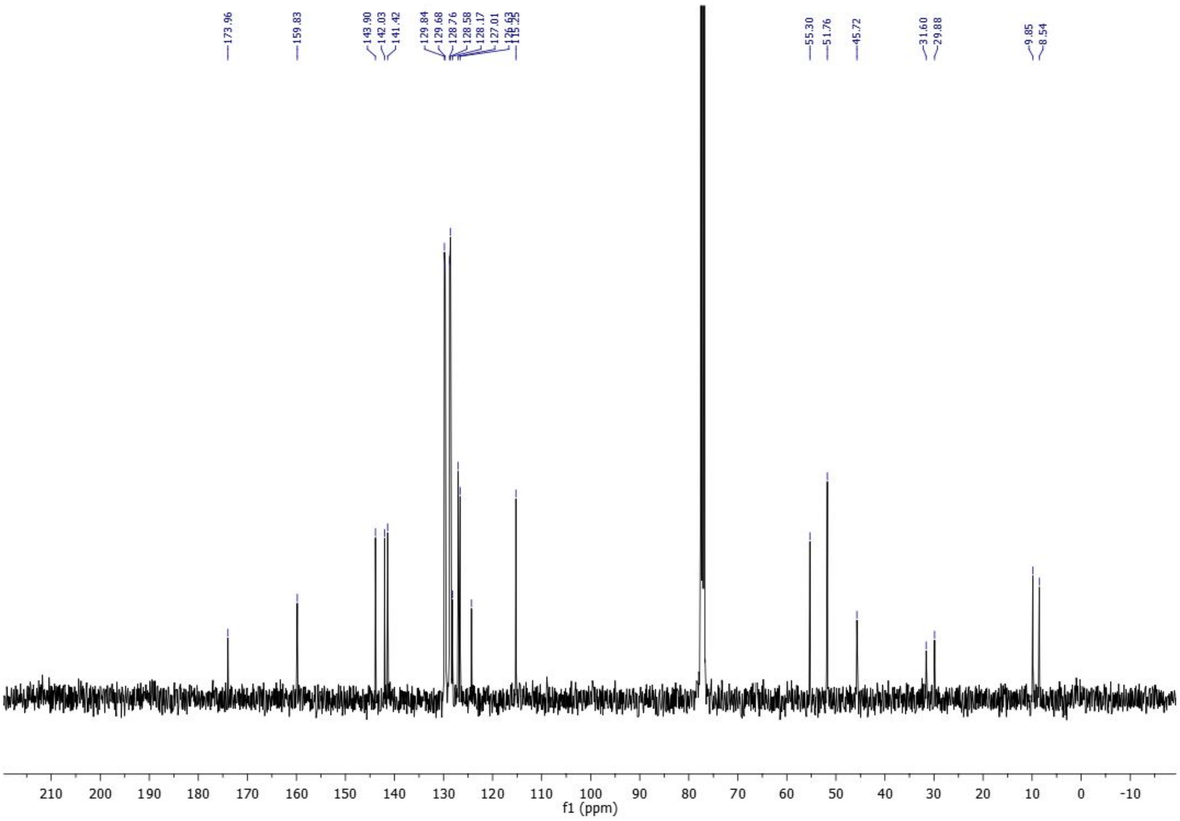

Au_2Br_24c

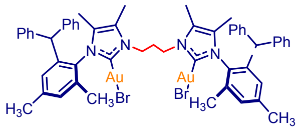

Au_2Br_24c

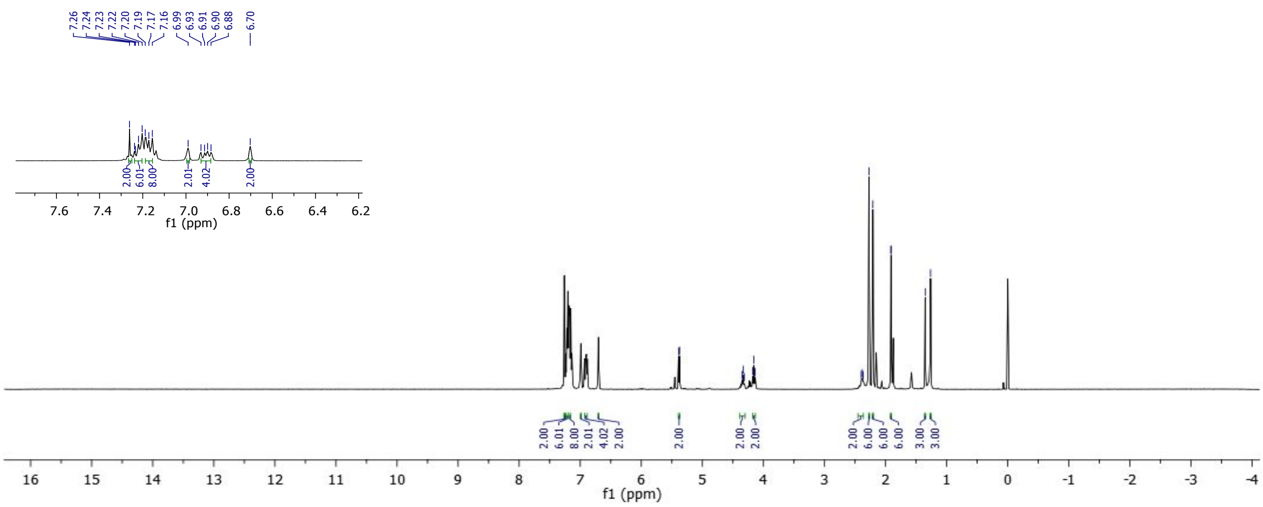

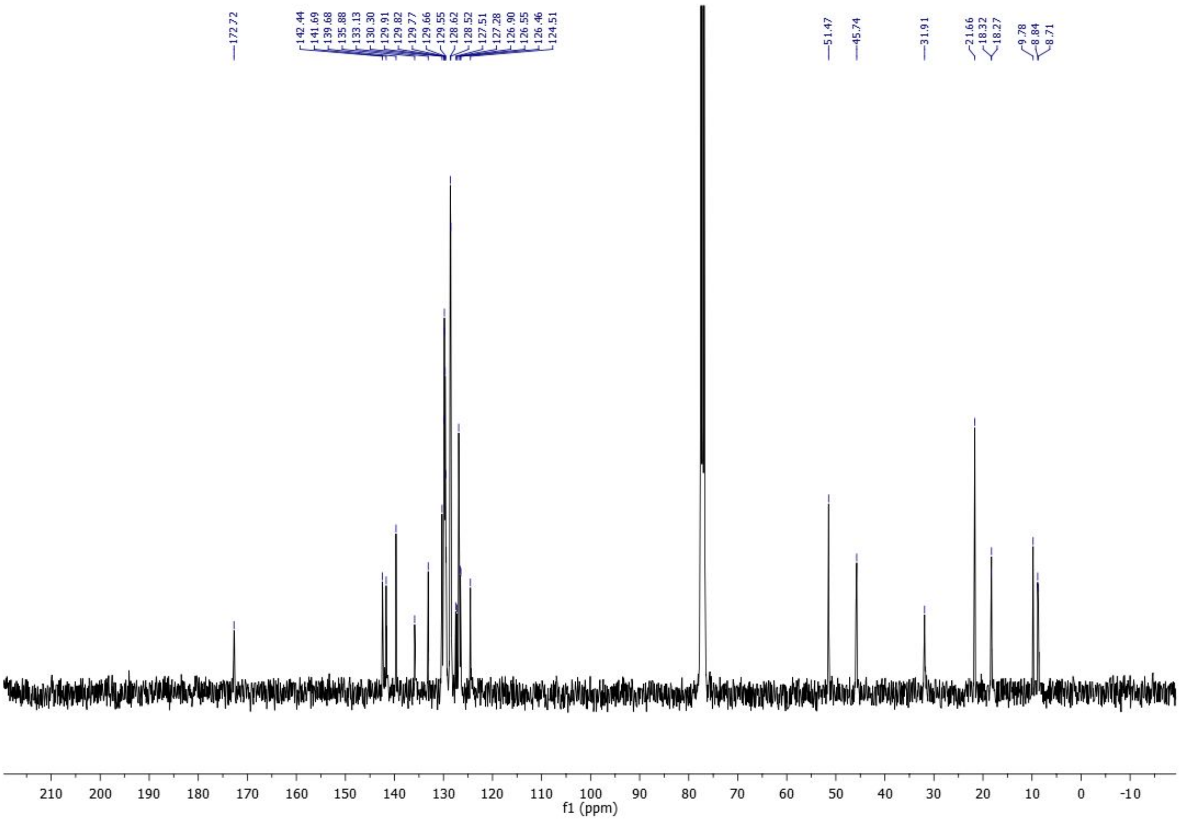

Au_2Br_24d

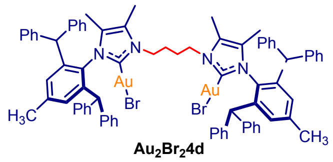
Au
Br
Au
Br
Ph
Ph
H3C
CH3

Au2Br24d

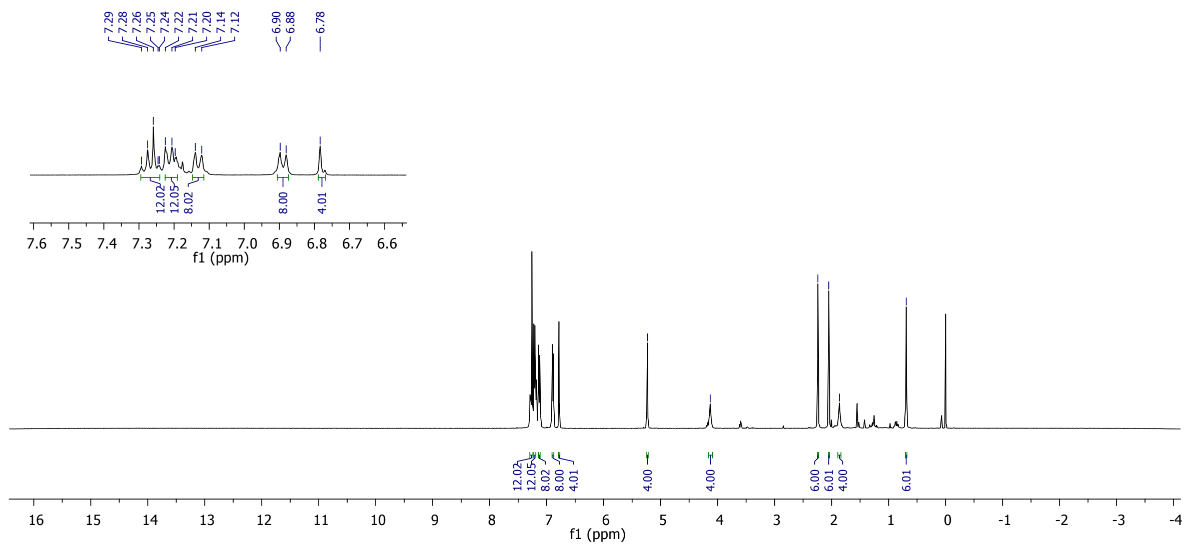
5.23
4.13
2.24
2.05
1.86
0.69
7.29
7.28
7.26
7.25
7.24
7.22
7.21
7.20
7.14
7.12
6.90
6.88
6.78
12.02
12.05
8.02
8.00
4.01
4.00
4.00
6.00
6.01
4.00
6.01
f1 (ppm)

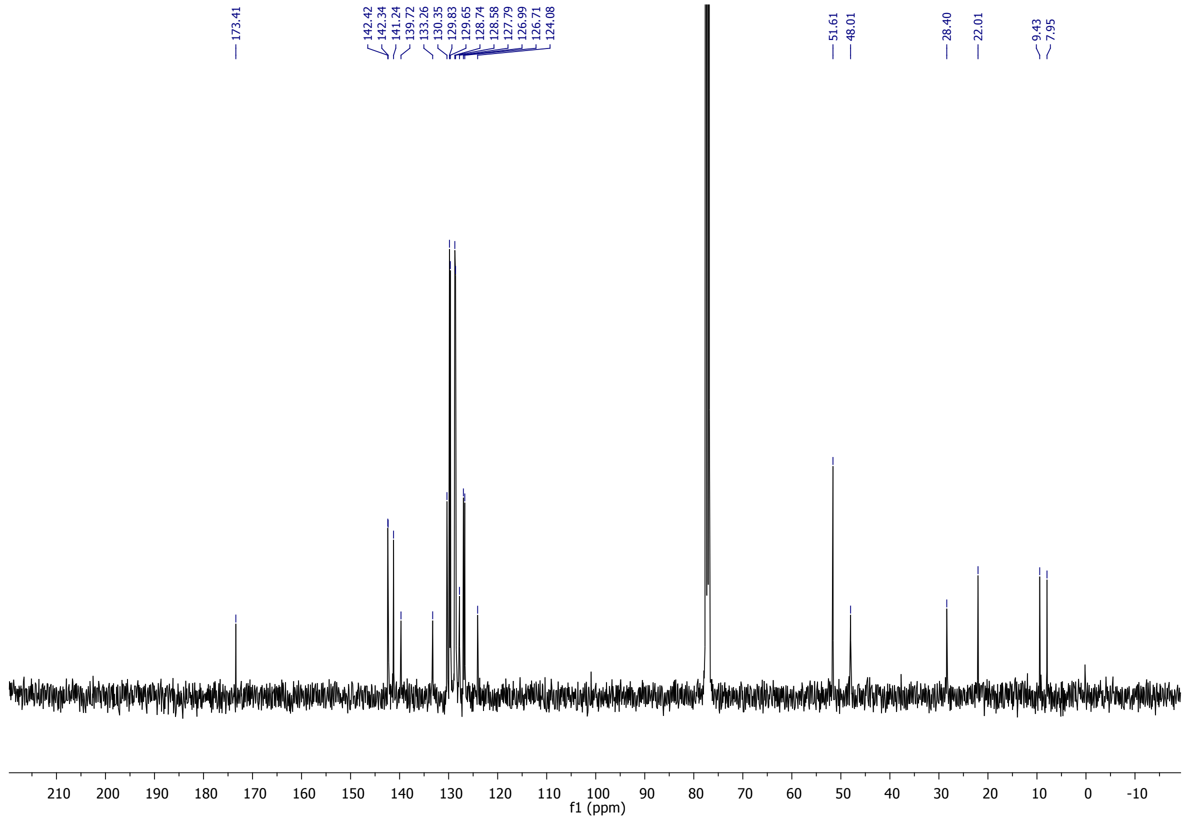
173.41
142.42
142.34
141.24
139.72
133.26
130.35
129.83
129.65
128.74
128.58
127.79
126.99
126.71
124.08
51.61
48.01
28.40
22.01
9.43
7.95
f1 (ppm)

Au_2Br_24e

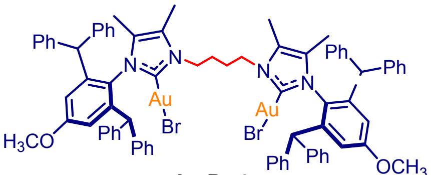

Au_2Br_24e

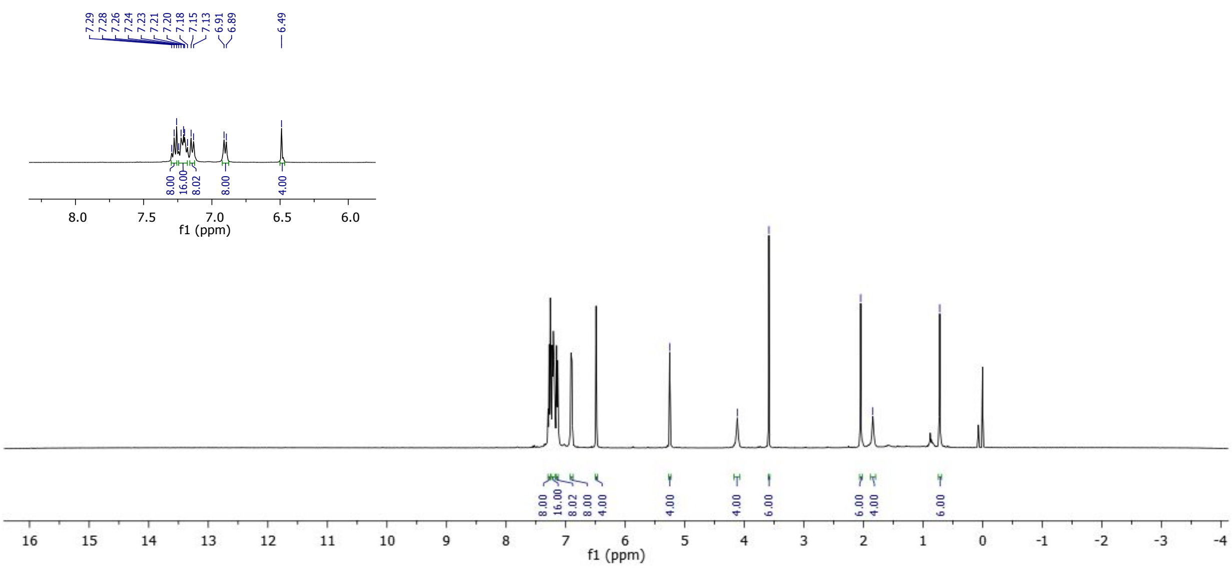

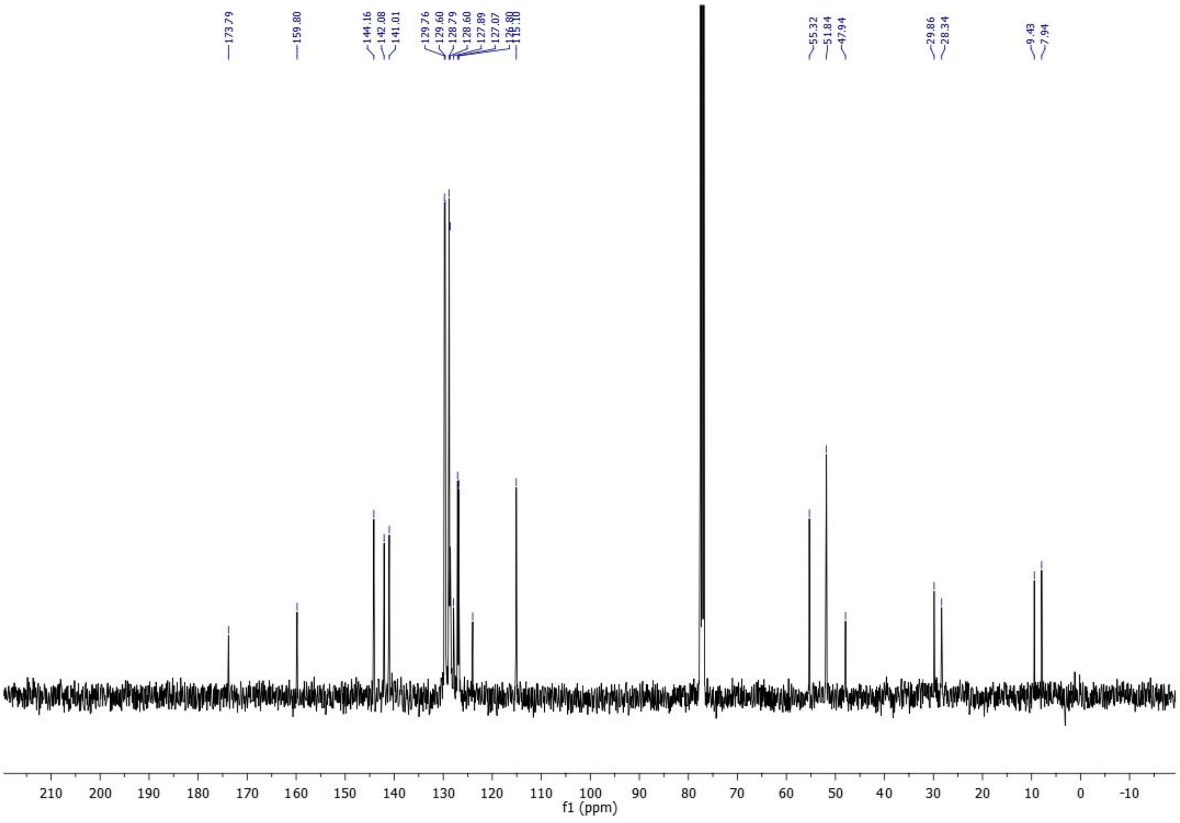

Au_2Br_24f

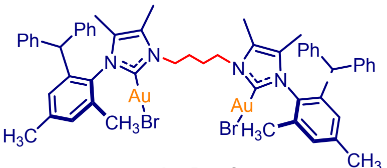

Au_2Br_24f

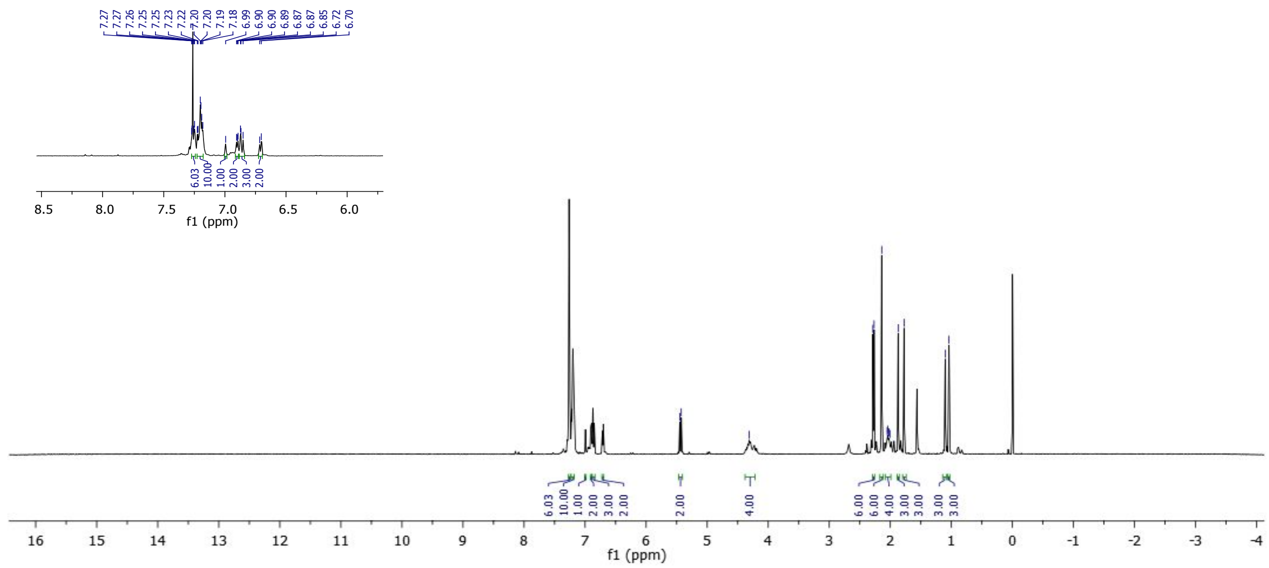

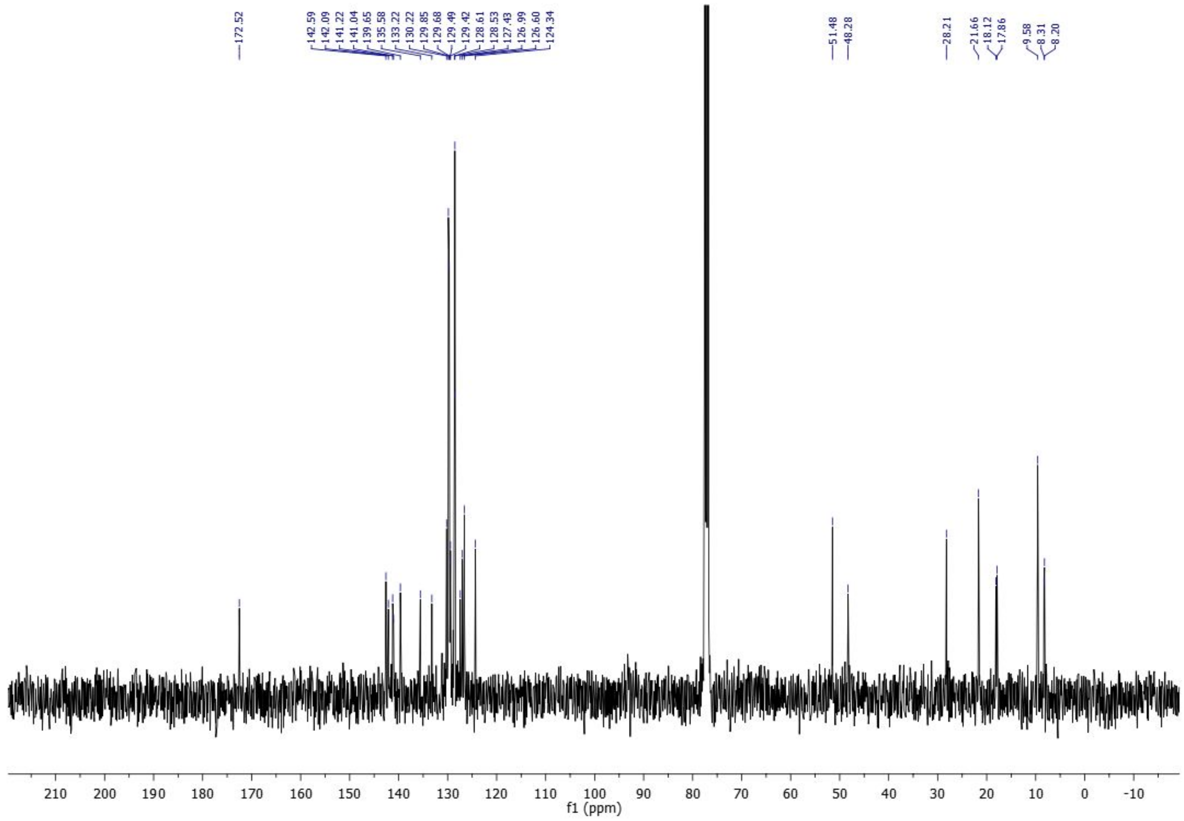

AuBr4g

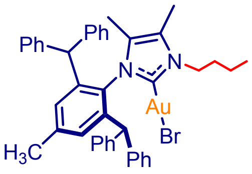
Ph
Ph
N
N
Au
Br
H3C
Ph
Ph

AuBr4g

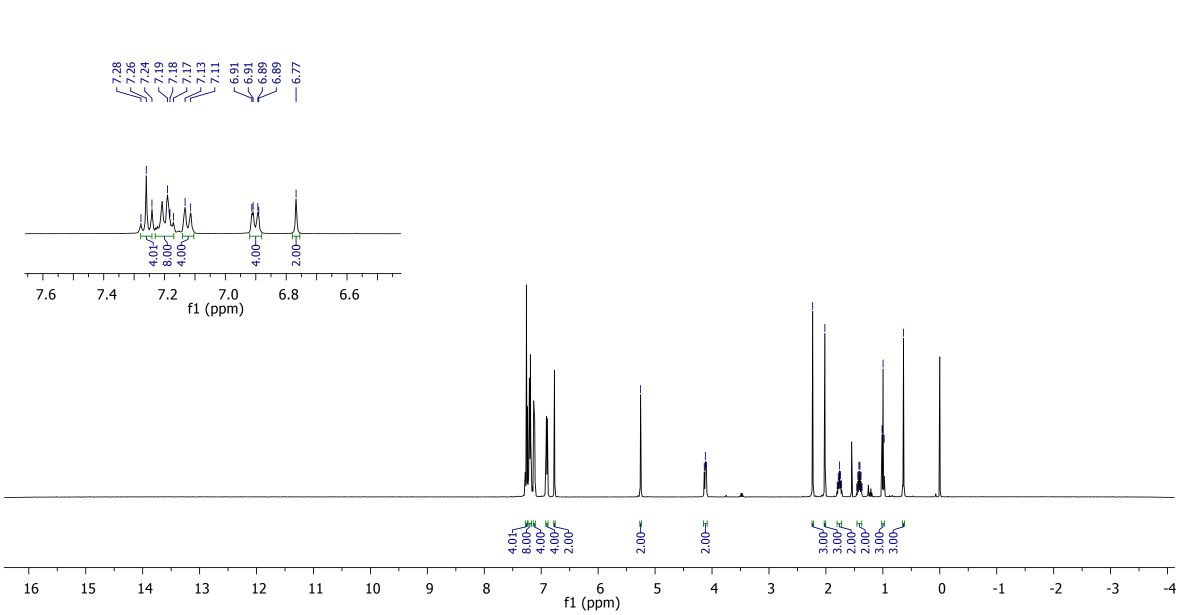
5.25
4.14
4.12
4.10
2.23
2.02
1.80
1.78
1.76
1.74
1.73
1.46
1.44
1.42
1.40
1.38
1.37
1.01
1.00
0.98
0.64
7.28
7.26
7.24
7.19
7.18
7.17
7.13
7.11
6.91
6.91
6.89
6.89
6.77
4.01
8.00
4.00
4.00
2.00
2.00
2.00
3.00
3.00
2.00
2.00
3.00
3.00
f1 (ppm)

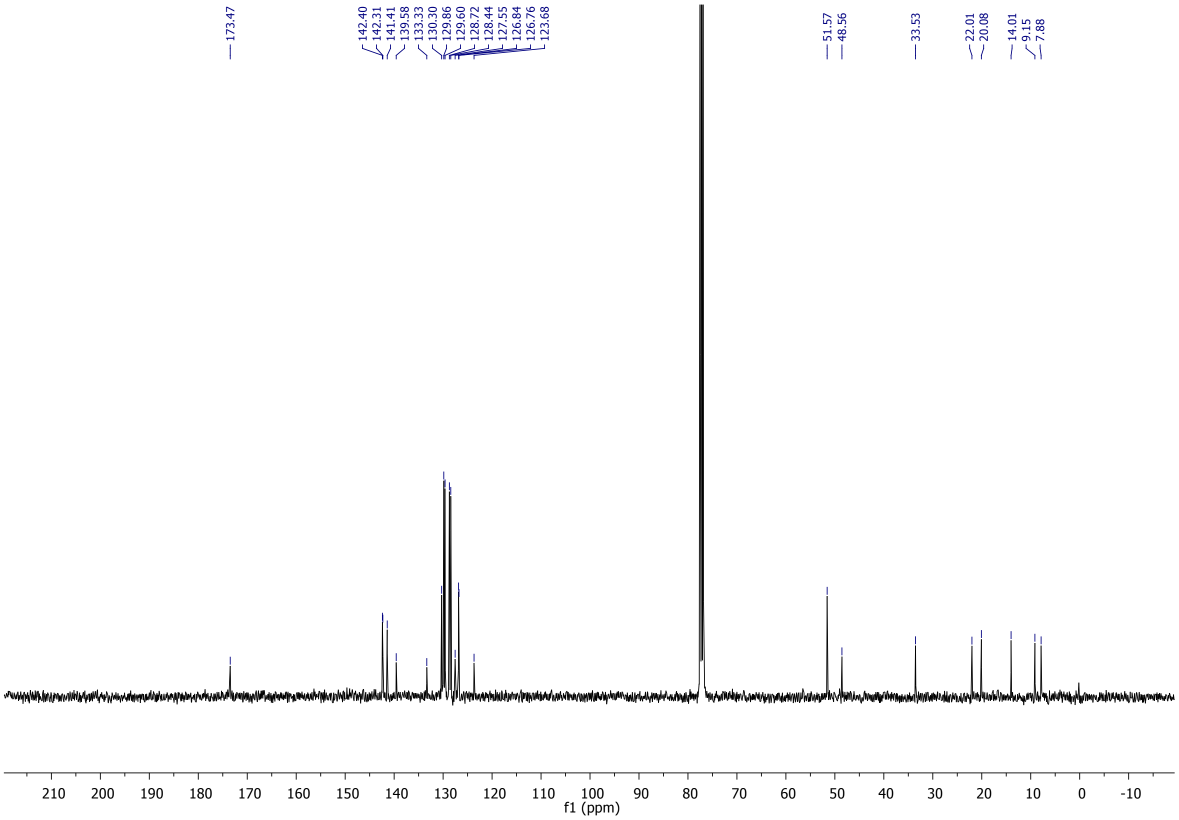
173.47
142.40
142.31
141.41
139.58
133.33
130.30
129.86
129.60
128.72
128.44
127.55
126.84
126.76
123.68
51.57
48.56
33.53
22.01
20.08
14.01
9.15
7.88
f1 (ppm)

6 3-Benzyl-5-vinyloxazolidin-2-one

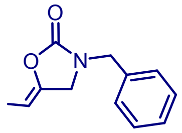

6

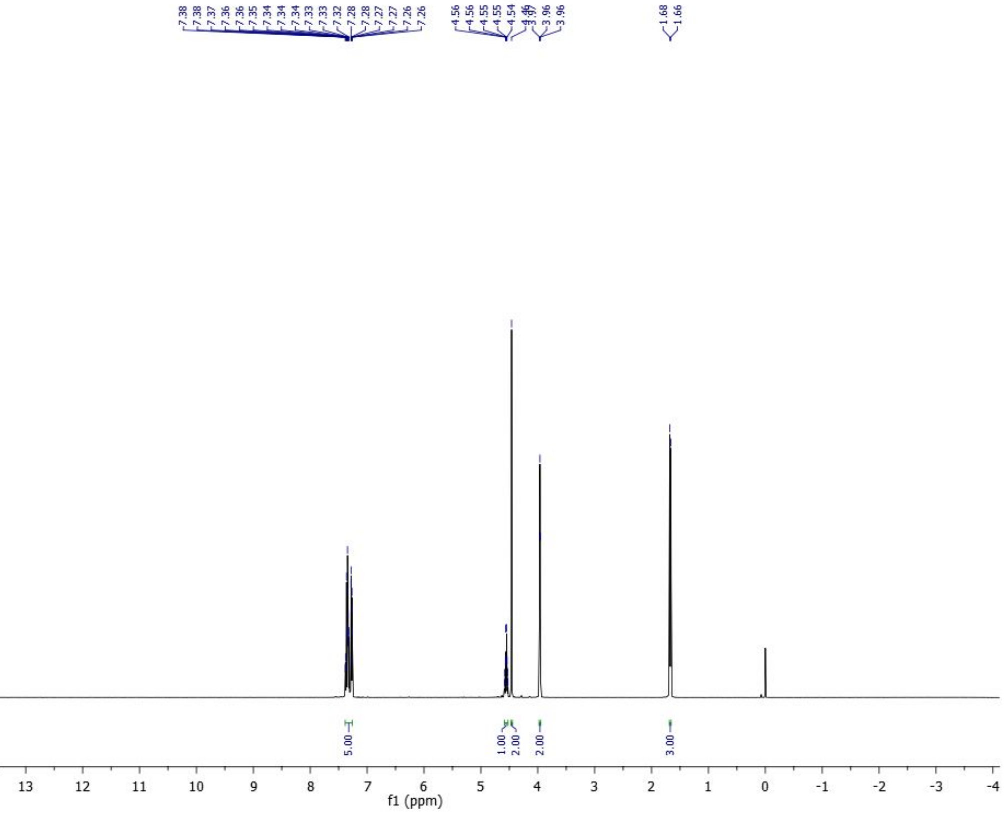

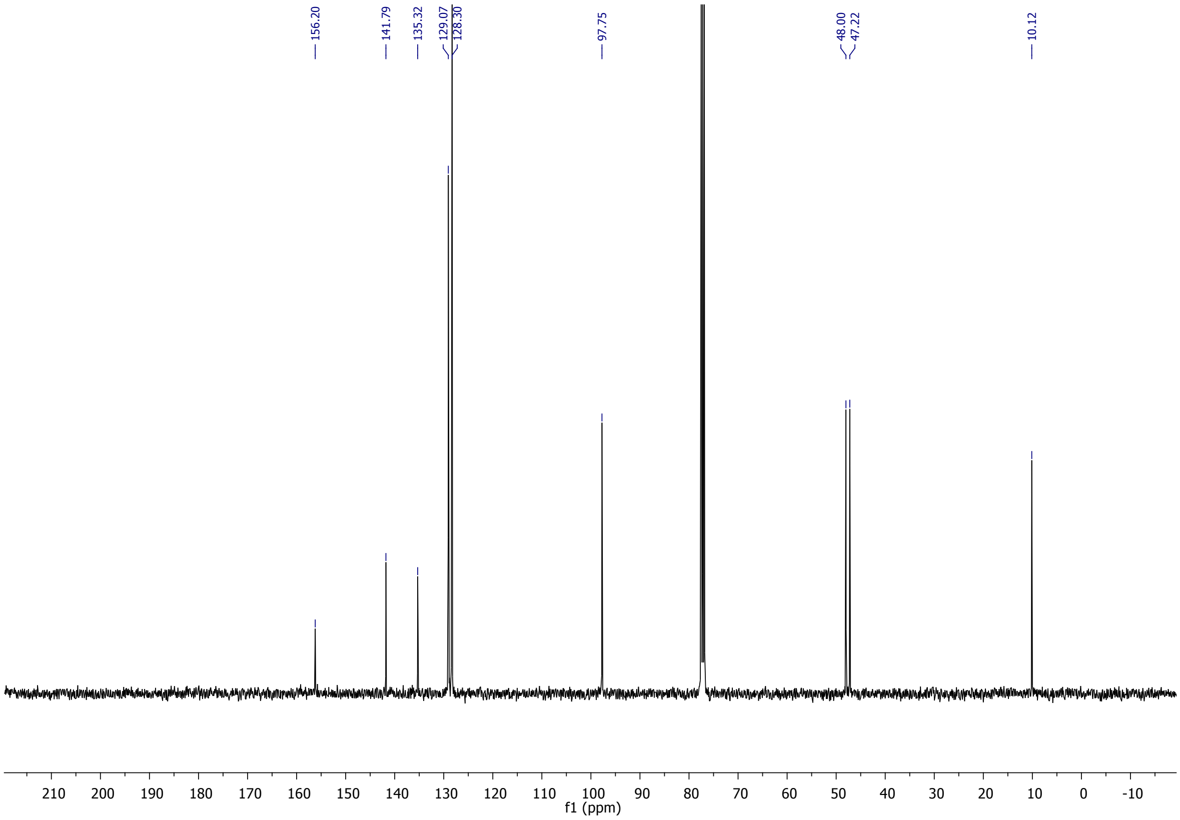

8 1,2-Diphenylethan-1-one

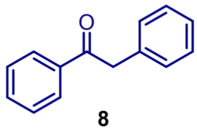

8

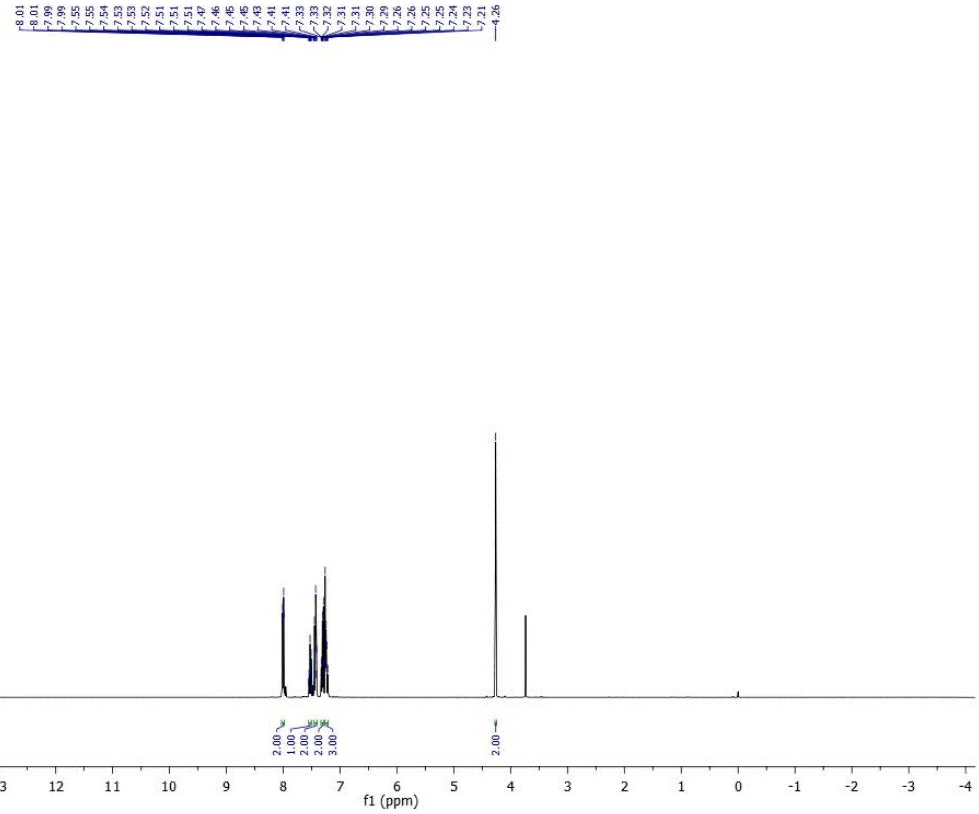

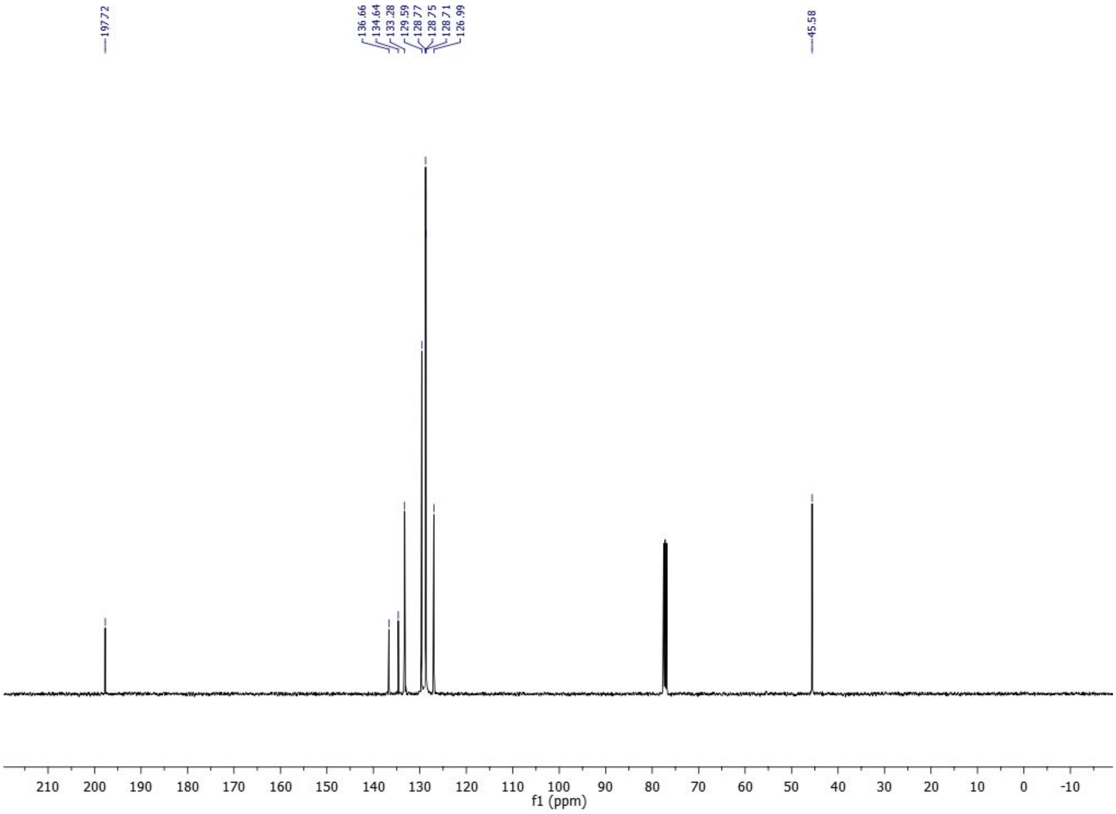

Cartesian Coordinates with Zero-Point Energies and Thermal Corrections

IPr*diNHC-C3

Energy: -3273.544662 au

Sum of electronic and thermal Energies: -3272.180078 au

Geometry:

C	-3.53431900	-1.97365100	-0.51231600
H	-2.83208100	-1.15385300	-0.35322300
N	-4.09359600	0.71385600	0.43746800
C	-7.35292100	2.83989600	-1.05385100
C	-3.02670800	-4.01690100	-2.00425900
H	-3.26849700	-4.67884000	-1.18113600
C	-3.09602300	-2.63333300	-1.82714400
C	-2.63621200	-4.56027400	-3.23041900
H	-2.59028400	-5.63763700	-3.34812700
N	-2.39442100	1.97313500	0.65715000
C	-7.21296200	-1.43961900	-1.47099100
C	-4.91686300	-1.32975400	-0.64842200
C	-3.38675700	-2.89275900	0.70264700
C	-5.16131800	-0.02533800	-0.18151800
C	-7.41430000	-0.13413900	-1.02356300
H	-8.36619400	0.34867500	-1.21678600
C	-3.23184500	1.48534800	-0.29822400
C	-2.75343700	-1.80263200	-2.90367800
H	-2.80366900	-0.72545900	-2.77709400
C	-7.07036300	3.32692300	2.17819200
H	-6.38137200	4.07170300	1.79263400
C	-2.30271200	-3.72692300	-4.29432200
H	-1.99895400	-4.14885600	-5.24604000
C	-8.83429900	1.43172800	3.17726600
H	-9.51909000	0.68337700	3.56139500
C	-4.43921200	-3.64683100	1.23028900
H	-5.42892700	-3.56927300	0.79673400

C	-1.23404500	2.79123700	0.31496500
H	-1.03011700	3.47880100	1.14062600
H	-1.51529700	3.38875600	-0.55397000
C	-8.69765900	3.21289700	-0.99571300
H	-9.29104600	2.95865600	-0.12517700
C	-6.60455100	3.20469100	-2.18157800
H	-5.55789000	2.92132800	-2.23621300
C	-4.58936700	-0.02738300	2.83425200
H	-5.62767300	0.31332600	2.86659900
H	-4.15851200	0.12265900	3.82537600
H	-4.59457300	-1.10173800	2.63313100
C	-1.94837800	1.92413000	3.17043500
H	-0.89082400	1.64787600	3.11005800
H	-2.36514700	1.43325700	4.05066200
H	-1.99732200	3.00475900	3.34406300
C	-2.12707200	-3.00498100	1.30534800
H	-1.29741800	-2.42465600	0.91486300
C	-7.33441400	2.18031300	1.41718500
C	-2.97708500	-4.59654000	2.90798400
H	-2.82123000	-5.25155400	3.75793700
C	-8.53513200	4.27858900	-3.16079700
H	-8.99094800	4.83487900	-3.97235100
C	-9.28587400	3.92452800	-2.04345300
H	-10.33152600	4.20581800	-1.97947100
C	-8.22298700	1.23489700	1.93746300
H	-8.43459100	0.33056400	1.37997200
C	-4.23622800	-4.49264100	2.32232400
H	-5.06775300	-5.06722200	2.71614200
C	-7.67580500	3.52641600	3.41550000
H	-7.45244600	4.42103400	3.98657500
C	-8.56451200	2.57722600	3.92103600
H	-9.03645000	2.72821000	4.88544000
C	-7.18844600	3.91721700	-3.22445100

H	-6.59199400	4.19302500	-4.08741800
C	-6.65449900	2.03916200	0.05319300
H	-5.66886000	2.49450100	0.16167400
C	-2.70687200	1.52099100	1.94940300
C	-5.95455100	-2.01199700	-1.28724800
H	-5.76160500	-3.00150100	-1.68761800
C	-6.41001300	0.59093100	-0.37847200
C	-3.79757500	0.71845800	1.81353900
C	-2.36057600	-2.34231200	-4.12447400
H	-2.09916100	-1.68290100	-4.94512000
C	-1.91895500	-3.84606200	2.39496300
H	-0.93321300	-3.91185800	2.84213000
C	6.65449900	2.03916200	-0.05320000
H	5.66886000	2.49449900	-0.16168700
N	4.09359700	0.71385500	-0.43747400
C	3.09602100	-2.63332300	1.82715000
C	8.69765400	3.21289800	0.99571500
H	9.29104600	2.95865200	0.12518400
C	7.35291500	2.83989900	1.05384600
C	9.28586300	3.92453100	2.04345700
H	10.33151600	4.20581900	1.97948100
N	2.39442200	1.97313300	-0.65716300
C	7.21296100	-1.43961400	1.47099400
C	6.41001300	0.59093200	0.37846800
C	7.33442100	2.18031000	-1.41719000
C	5.16131800	-0.02533700	0.18151500
C	5.95455000	-2.01199300	1.28725100
H	5.76160400	-3.00149600	1.68762400
C	3.23184600	1.48535000	0.29821300
C	6.60453800	3.20470000	2.18156600
H	5.55787700	2.92133900	2.23619500
C	2.12707100	-3.00499500	-1.30533700
H	1.29741700	-2.42467100	-0.91485300

C	8.53511400	4.27859800	3.16079400
H	8.99092600	4.83489000	3.97234900
C	4.23623000	-4.49265300	-2.32230900
H	5.06775700	-5.06723300	-2.71612700
C	8.22299200	1.23489200	-1.93746400
H	8.43459200	0.33055800	-1.37997300
C	0.00000000	1.94021900	-0.00000600
H	-0.23773500	1.29051400	-0.84742400
H	0.23773600	1.29051900	0.84741500
C	3.02671700	-4.01689000	2.00427500
H	3.26851400	-4.67883400	1.18115900
C	2.75342500	-1.80261600	2.90367600
H	2.80364900	-0.72544400	2.77708300
C	4.58937000	-0.02739400	-2.83425500
H	4.59457400	-1.10174900	-2.63313000
H	4.15851700	0.12264400	-3.82538000
H	5.62767700	0.31331300	-2.86660000
C	1.94837900	1.92411400	-3.17044800
H	1.99732000	3.00474200	-3.34408000
H	2.36515000	1.43323800	-4.05067300
H	0.89082500	1.64785600	-3.11007100
C	7.07037600	3.32692100	-2.17819700
H	6.38138500	4.07170300	-1.79264200
C	3.38675700	-2.89276500	-0.70264000
C	8.56452800	2.57722100	-3.92103600
H	9.03647000	2.72820300	-4.88543900
C	2.30270900	-3.72689900	4.29433400
H	1.99895100	-4.14882700	5.24605400
C	2.63622000	-4.56025600	3.23043800
H	2.59030000	-5.63761900	3.34815500
C	4.43921500	-3.64683600	-1.23027900
H	5.42893000	-3.56927200	-0.79672700
C	8.83430900	1.43172100	-3.17726500

H	9.51909900	0.68336800	-3.56139200
C	1.91895600	-3.84608200	-2.39494600
H	0.93321200	-3.91188400	-2.84211100
C	2.97708700	-4.59655900	-2.90796600
H	2.82123200	-5.25157900	-3.75791500
C	2.36056300	-2.34229000	4.12447400
H	2.09913900	-1.68287400	4.94511400
C	3.53431900	-1.97365000	0.51231800
H	2.83208100	-1.15385200	0.35321900
C	2.70687300	1.52098200	-1.94941400
C	7.41430000	-0.13413600	1.02356200
H	8.36619300	0.34867900	1.21678500
C	4.91686200	-1.32975300	0.64842200
C	3.79757700	0.71845000	-1.81354600
C	1.23404500	2.79123600	-0.31498300
H	1.51529700	3.38876000	0.55394900
H	1.03011700	3.47879500	-1.14064700
C	7.18842700	3.91722900	3.22444000
H	6.59197000	4.19304100	4.08740200
C	7.67582200	3.52641300	-3.41550300
H	7.45246800	4.42103200	-3.98657900
C	-8.32322100	-2.20724400	-2.14779700
H	-9.00128500	-1.53734800	-2.68152900
H	-7.92683800	-2.93165300	-2.86296100
H	-8.92041400	-2.76257000	-1.41585400
C	8.32322000	-2.20723800	2.14780200
H	8.92040900	-2.76257000	1.41586100
H	9.00128600	-1.53734000	2.68152800
H	7.92683600	-2.93164100	2.86297200

IPr*diNHC-C4

Energy: -3312.868738 au
Sum of electronic and thermal Energies: -3311.474490 au
Geometry:

C	-6.94669800	-2.13615700	-0.20917600

H	-5.86443700	-2.22014800	-0.09877100
N	-5.04189000	-0.05113100	0.48701800
C	-5.29899400	3.70363400	-1.27899200
C	-8.41598000	-3.80053200	-1.52173900
H	-9.07200600	-3.89768900	-0.66454500
C	-7.29514300	-2.97028100	-1.44915200
C	-8.69526900	-4.52076500	-2.68508500
H	-9.57020900	-5.16086400	-2.72195200
N	-3.01405600	-0.67585000	0.63878700
C	-8.71304100	1.08801000	-1.31301700
C	-7.23861400	-0.65107800	-0.43919100
C	-7.54554800	-2.70026400	1.08159100
C	-6.29978300	0.33767000	-0.09305000
C	-7.74986200	2.04131500	-0.98437500
H	-7.92495100	3.07965600	-1.24479700
C	-3.95747100	-0.36460800	-0.29142300
C	-6.45126000	-2.88443200	-2.56509800
H	-5.57633300	-2.24323800	-2.51871100
C	-4.58571800	4.02368100	1.90723400
H	-3.59859700	3.86544300	1.48466600
C	-7.85324100	-4.42559800	-3.78934800
H	-8.06787400	-4.98827200	-4.69111300
C	-7.10264500	4.43707900	3.00005600
H	-8.09027400	4.59015400	3.42156600
C	-8.71946500	-2.21401300	1.66427800
H	-9.23542700	-1.37372300	1.21584900
C	-0.71047600	0.19068200	0.21237900
H	-0.68485200	0.67457500	1.19553500
H	-1.13440200	0.91992600	-0.48641500
C	-5.75626900	5.02311200	-1.29832600
H	-6.25078500	5.43575700	-0.42674400
C	-4.64212100	3.20529900	-2.41283700
H	-4.28298300	2.18063800	-2.40852100
C	-5.79160000	0.11012600	2.92636500
H	-6.15769300	1.13900900	2.87745500

H	-5.34990200	-0.03868600	3.91294000
H	-6.65366900	-0.55694600	2.84306300
C	-2.64979800	-0.87187200	3.15853700
H	-2.31924700	-1.91619800	3.16628000
H	-3.22683000	-0.70574400	4.06901900
H	-1.75461700	-0.24446200	3.21772400
C	-6.89631400	-3.77529700	1.70328300
H	-5.98233600	-4.16608800	1.26739100
C	-5.70586200	3.51043100	1.23980500
C	-8.57846900	-3.85740100	3.43412400
H	-8.97533000	-4.30086800	4.34045700
C	-4.92170200	5.31964600	-3.54903700
H	-4.77558600	5.94347900	-4.42387000
C	-5.57168900	5.82494700	-2.42682800
H	-5.93355300	6.84750300	-2.42240700
C	-6.96546200	3.72562400	1.80648500
H	-7.84900100	3.32770000	1.32249000
C	-9.23291400	-2.78803100	2.82900300
H	-10.14467400	-2.39277300	3.26374500
C	-4.71733500	4.73200300	3.09811100
H	-3.83424600	5.11657800	3.59670200
C	-5.98083200	4.94365300	3.65043300
H	-6.08718100	5.49313100	4.57905400
C	-4.45458200	4.00429400	-3.53642900
H	-3.94269700	3.60112900	-4.40362900
C	-5.48905100	2.76521900	-0.07955100
H	-4.54006800	2.23756600	0.02812700
C	-3.48088100	-0.56025200	1.95804500
C	-8.43137400	-0.25148100	-1.04575200
H	-9.14150100	-1.01123000	-1.35399900
C	-6.54151500	1.69399900	-0.37620900
C	-4.77960000	-0.16342000	1.86506400
C	-1.64655700	-1.02220200	0.25952300
H	-1.70736300	-1.48439500	-0.72680600
H	-1.26390800	-1.77305800	0.95668200

C	-6.72626700	-3.60476900	-3.72344400
H	-6.05963500	-3.52753000	-4.57559100
C	-7.40328800	-4.34933500	2.86546700
H	-6.88044600	-5.17891700	3.32891600
C	6.94669800	2.13615700	0.20917300
H	5.86443700	2.22014800	0.09876800
N	5.04189000	0.05113100	-0.48701900
C	5.29899500	-3.70363300	1.27899600
C	8.41598000	3.80053300	1.52173700
H	9.07200600	3.89768900	0.66454300
C	7.29514200	2.97028200	1.44914900
C	8.69526800	4.52076600	2.68508200
H	9.57020900	5.16086500	2.72194900
N	3.01405600	0.67584900	-0.63878900
C	8.71304100	-1.08800900	1.31301500
C	7.23861400	0.65107900	0.43919000
C	7.54554900	2.70026400	-1.08159300
C	6.29978200	-0.33767000	0.09305000
C	7.74986200	-2.04131500	0.98437500
H	7.92495200	-3.07965500	1.24479800
C	3.95747100	0.36460800	0.29142100
C	6.45125900	2.88443300	2.56509500
H	5.57633200	2.24324000	2.51870800
C	4.58571300	-4.02368200	-1.90722800
H	3.59859200	-3.86544500	-1.48465700
C	7.85324000	4.42560000	3.78934500
H	8.06787300	4.98827400	4.69110900
C	7.10263800	-4.43708000	-3.00005400
H	8.09026600	-4.59015400	-3.42156600
C	8.71946500	2.21401200	-1.66428000
H	9.23542600	1.37372100	-1.21585200
C	0.71047700	-0.19068300	-0.21237900
H	0.68485300	-0.67457900	-1.19553400
H	1.13440300	-0.91992600	0.48641700
C	5.75627000	-5.02311200	1.29832900

H	6.25078300	-5.43575700	0.42674400
C	4.64212700	-3.20529800	2.41284300
H	4.28298900	-2.18063700	2.40852800
C	5.79160000	-0.11012900	-2.92636600
H	6.15768500	-1.13901400	-2.87746100
H	5.34990600	0.03869400	-3.91294100
H	6.65367500	0.55693700	-2.84305900
C	2.64979900	0.87187200	-3.15853900
H	2.31926100	1.91620100	-3.16629000
H	3.22682600	0.70572700	-4.06902100
H	1.75460900	0.24447300	-3.21771700
C	6.89631600	3.77529800	-1.70328500
H	5.98233900	4.16608900	-1.26739300
C	5.70585800	-3.51043200	-1.23980100
C	8.57847100	3.85740100	-3.43412600
H	8.97533300	4.30086700	-4.34045900
C	4.92171100	-5.31964500	3.54904200
H	4.77559700	-5.94347800	4.42387600
C	5.57169400	-5.82494600	2.42683100
H	5.93355700	-6.84750300	2.42240900
C	6.96545700	-3.72562400	-1.80648300
H	7.84899600	-3.32769900	-1.32249000
C	9.23291500	2.78802900	-2.82900500
H	10.14467500	2.39277100	-3.26374700
C	4.71732800	-4.73200500	-3.09810400
H	3.83423800	-5.11658100	-3.59669400
C	5.98082400	-4.94365400	-3.65042900
H	6.08717200	-5.49313300	-4.57905000
C	4.45459100	-4.00429300	3.53643600
H	3.94270900	-3.60112800	4.40363700
C	5.48905000	-2.76521900	0.07955400
H	4.54006600	-2.23756600	-0.02812100
C	3.48088100	0.56025100	-1.95804700
C	8.43137400	0.25148100	1.04575000
H	9.14150100	1.01123100	1.35399600

C	6.54151400	-1.69399900	0.37621000
C	4.77960100	0.16342000	-1.86506600
C	1.64655700	1.02220100	-0.25952600
H	1.70736100	1.48439700	0.72680200
H	1.26390700	1.77305500	-0.95668800
C	6.72626600	3.60477100	3.72344100
H	6.05963300	3.52753400	4.57558700
C	7.40329000	4.34933600	-2.86546900
H	6.88044900	5.17891800	-3.32891800
C	-10.01920500	1.49327400	-1.95306000
H	-10.40520400	0.70398000	-2.60218800
H	-10.78316300	1.69592800	-1.19405400
H	-9.90406900	2.40028900	-2.55089100
C	10.01920600	-1.49327300	1.95305600
H	10.78316500	-1.69591900	1.19404900
H	10.40520300	-0.70398100	2.60218900
H	9.90407400	-2.40029200	2.55088100

IPr*$^{n\text{-Bu}}$

Energy: -1735.684900 au
Sum of electronic and thermal Energies: -1734.919220 au
Geometry:

N	-0.18905400	-2.84316700	1.08534600
C	-0.09695700	-1.50748200	1.33610800
C	-0.10426300	-3.15195300	-0.28260100
C	1.61400700	2.37935900	-0.25312300
H	2.59825800	2.82824800	-0.17367400
C	-2.92424200	0.34140300	-1.67790100
C	-3.84128500	-0.71049500	-1.80364500
H	-4.10183800	-1.29430000	-0.92658800
C	-2.34440000	0.64194100	-0.29362000
H	-2.25559900	-0.32262600	0.20893900
C	-0.13329700	-4.54340100	-0.82365300
H	0.66723200	-5.16060400	-0.40188300
H	0.00317000	-4.52813100	-1.90566100
H	-1.08077400	-5.05152800	-0.61867000
C	2.72399700	0.13749000	0.18835300
H	2.35693200	-0.78427400	0.64225800

C	3.19802200	0.75850200	2.58515700
H	2.25493300	0.27976800	2.83044900
C	0.50838200	3.20280300	-0.46436600
N	0.04255900	-0.98200500	0.07645200
C	3.62466800	0.78257800	1.25014900
C	3.96509400	1.34204000	3.58884100
H	3.61959200	1.31045200	4.61652600
C	-2.80812300	-3.22237900	2.80211800
H	-2.81790500	-2.40853800	2.07026200
H	-3.81179100	-3.66199400	2.78726600
C	3.48769100	-0.28827400	-1.06811900
C	-4.30450300	2.27948800	0.06918500
H	-4.47513800	2.31329600	-1.00043700
C	1.48884800	0.99825300	-0.08932500
C	-4.42340100	-1.01713300	-3.03030200
H	-5.12845400	-1.83820400	-3.10176700
C	-0.75596900	2.61514100	-0.48026300
H	-1.63104800	3.24851000	-0.57852700
C	-0.36384700	-3.82410800	2.15662000
H	0.01062600	-3.34915000	3.06308500
H	0.27886600	-4.68427400	1.94848500
C	-3.28519800	1.47676300	0.58607000
C	4.21034500	-1.48846000	-1.03336400
H	4.19580000	-2.08949200	-0.12970000
C	4.84096600	1.39953500	0.94861900
H	5.20054300	1.41307100	-0.07371900
C	0.67555800	4.69102100	-0.65727200
H	1.54266100	5.06749600	-0.10966800
H	-0.20655400	5.23748100	-0.31598200
H	0.82455300	4.93647400	-1.71479100
C	4.97432900	-1.15450500	-3.29985100
H	5.54456100	-1.48818800	-4.15956700
C	-3.18699100	0.77366800	-4.05537200
H	-2.92209900	1.35700700	-4.93062600
C	0.20181700	0.43173900	-0.13855100

C	-3.10383800	1.43652700	1.97559600
H	-2.31461300	0.81618200	2.38901200
C	-2.52916900	-2.64612700	4.19388400
H	-2.48710300	-3.43750000	4.95026300
H	-3.31760400	-1.94841200	4.48899100
H	-1.58414300	-2.09833100	4.21859200
C	-0.93523700	1.23914900	-0.32089900
C	-1.81192500	-4.30101600	2.35203900
H	-2.16916600	-4.75713500	1.42261700
H	-1.78916300	-5.10655100	3.09713000
C	-5.11850900	3.03272500	0.91809100
H	-5.90524100	3.64971000	0.49738900
C	4.25914100	0.03876000	-3.34990400
H	4.26912700	0.64069000	-4.25219800
C	-4.09868700	-0.27286300	-4.16464300
H	-4.54851100	-0.51006800	-5.12224400
C	-2.60429500	1.07632300	-2.82323100
H	-1.88677500	1.88539500	-2.76092400
C	0.04522100	-1.96421500	-0.93048200
C	5.17533800	1.96334900	3.27666600
H	5.77442600	2.41736900	4.05821800
C	5.60967400	1.98902800	1.95446500
H	6.55189800	2.46277300	1.70068100
C	4.94557400	-1.91971500	-2.13358500
H	5.49448300	-2.85377000	-2.08218300
C	3.52133900	0.46647200	-2.24419700
H	2.96048800	1.39104400	-2.30698500
C	0.20057600	-1.66177400	-2.38306300
H	1.15020600	-1.16169200	-2.59101100
H	-0.60402300	-1.01871500	-2.74908500
H	0.17702200	-2.58556200	-2.96324700
C	-3.91647100	2.18352100	2.82265200
H	-3.76172100	2.13704400	3.89519900
C	-4.92800000	2.98845200	2.29604400
H	-5.56251900	3.57044100	2.95521400
